# A single arm phase Ib/II trial of first-line pembrolizumab, trastuzumab and chemotherapy for advanced HER2-positive gastric cancer

**DOI:** 10.1038/s41467-022-33267-z

**Published:** 2022-10-12

**Authors:** Choong-kun Lee, Sun Young Rha, Hyo Song Kim, Minkyu Jung, Beodeul Kang, Jingmin Che, Woo Sun Kwon, Sejung Park, Woo Kyun Bae, Dong-Hoe Koo, Su-Jin Shin, Hyunki Kim, Hei-Cheul Jeung, Dae Young Zang, Sang Kil Lee, Chung Mo Nam, Hyun Cheol Chung

**Affiliations:** 1 Division of Medical Oncology, Department of Internal Medicine, Yonsei Cancer Center, Yonsei University College of Medicine, Seoul, South Korea; 2 Sondang Institute for Cancer Research, Yonsei University College of Medicine, Seoul, South Korea; 3 Department of Medical Oncology, CHA Bundang Medical Center, CHA University, Seongnam, South Korea; 4 Department of Biostatistics and Computing, Yonsei University College of Medicine, Seoul, South Korea; 5 Division of Hematology-Oncology, Department of Internal Medicine, Chonnam National University Medical School and Hwasun Hospital, Jeollanam-do, South Korea; 6 Division of Hematology/Oncology, Department of Internal Medicine, Kangbuk Samsung Hospital, Sungkyunkwan University School of Medicine, Seoul, South Korea; 7 Department of Pathology, Yonsei University College of Medicine, Seoul, South Korea; 8 Department of Internal Medicine, Gangnam Severance Hospital, Yonsei University College of Medicine, Seoul, South Korea; 9 Division of Hematology-Oncology, Department of Internal Medicine, Hallym University Medical Center, Hallym University College of Medicine, Anyang, South Korea; 10 Division of Gastroenterology, Department of Internal Medicine, Yonsei University College of Medicine, Seoul, South Korea; 11 Department of Preventive Medicine, Yonsei University College of Medicine, Seoul, South Korea

**Keywords:** Gastric cancer, Cancer immunotherapy, Gastric cancer, Cancer genomics

## Abstract

In this multi-center phase II trial, we evaluated the efficacy and safety of a quadruplet regimen (pembrolizumab, trastuzumab, and doublet chemotherapy) as first-line therapy for unresectable or metastatic human epidermal growth factor receptor 2 (HER2)-positive advanced gastric cancer (AGC) (NCT02901301). The primary endpoints were recommended phase 2 dose (RP2D) for phase Ib and objective response rate (ORR) for phase II. The secondary endpoints included progression-free survival (PFS), overall survival (OS), duration of response, time to response and safety. Without dose-limiting or unexpected toxicities, the starting dose in the phase Ib trial was selected as RP2D. In 43 patients, the primary endpoint was achieved: the objective response rate was 76.7% (95% confidence interval [CI]: 61.4–88.2), with complete and partial responses in 14% and 62.8% of patients, respectively. The median progression-free survival, overall survival, and duration of response were 8.6 months, 19.3 months, and 10.8 months, respectively. No patients discontinued pembrolizumab because of immune-related adverse events. Programmed death ligand-1 status was not related to survival. *Post hoc* analyses of pretreatment tumor specimens via targeted sequencing indicated that *ERBB2* amplification, RTK/RAS pathway alterations, and high neoantigen load corrected by HLA-B were positively related to survival. The current quadruplet regimen shows durable efficacy and safety for patients with HER2-positive AGC.

## Abstract (web summary)

In patients with advanced gastric cancer (AGC), resistance to treatment with trastuzumab and cytotoxic chemotherapy remains high. Here, the authors report the results of a phase Ib/II clinical trial assessing the safety and clinical response to a quadruplet regimen of pembrolizumab, trastuzumab, capecitabine, and cisplatin as a first-line therapy for HER2-positive AGC.

## Introduction

Gastric cancer is the third leading cause of cancer-related deaths and the fifth most common cancer worldwide^1^. Human epidermal growth factor receptor 2 (HER2) is a transmembrane tyrosine kinase receptor overexpressed or amplified in 10–20% of patients with gastric cancer^2^. The addition of the anti-HER2 antibody trastuzumab to cytotoxic chemotherapy for HER2-positive gastric cancer as a first-line therapy showed an overall survival benefit in the Trastuzumab for Gastric Cancer (ToGA) study^3^ and became the standard-of-care. However, as most patients show progression within a year, elucidating the molecular mechanisms underlying trastuzumab resistance is imperative. Preclinical and clinical evidence supports combining anti-HER2 treatment with immune checkpoint inhibitors, such as anti-programmed cell death protein 1 (PD-1) antibodies, which may help to overcome intrinsic or acquired resistance by synergistic anti-tumor effect^4–9^. In HER2-negative patients with gastric cancer, first-line chemotherapy plus anti-PD-1 inhibitor treatment showed a survival benefit in the ATTRACTION-4 (progression-free survival, PFS) and CheckMate-649 (PFS and overall survival, OS) trials^10,11^ when compared with chemotherapy alone.

Here, we report the results of a single-arm, multi-institutional phase Ib/II trial evaluating a quadruplet regimen of pembrolizumab, trastuzumab, capecitabine, and cisplatin as a first-line therapy for HER2-positive advanced gastric cancer (AGC) with *post hoc* genomic studies (PANTHERA trial).

## Results

### Patients

**Fig. 1:**
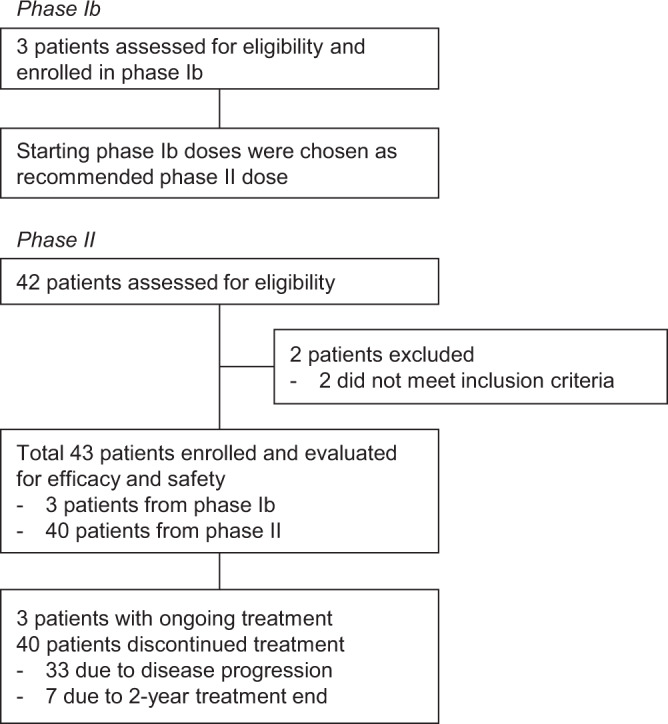
Trial profile. Diagram indicating participant numbers and disposition through the course of the trial.

Forty-three patients were enrolled in the phase Ib (*n* = 3) or phase II (*n* = 40) trial between February 2017 and March 2019. Of the 42 patients screened for phase II, two were excluded based on the eligibility criteria (Fig. 1).

Table 1 shows the baseline patient characteristics. The median patient age was 63 (range, 34–82) years, and most patients were male (*n* = 33, 76.7%). Twelve patients (27.9%) had previously undergone curative gastrectomy before recurrence. The highest frequency of metastasis was observed in the lymph nodes (*n* = 36, 83.7%) and the liver (*n* = 21, 48.8%). All patients were HER2-positive based on immunohistochemistry (IHC) 3 + (*n* = 30, 69.8%) or IHC 2+ with silver in situ hybridization (SISH) positivity (*n* = 13, 30.2%; median amplification index = 2.74; range, 2.05–4.07). Pretreatment programmed death-ligand 1(PD-L1) status was evaluated in 38 patients using the 22C3 antibody; 21 (48.8%) patients had a PD-L1 combined positive score (CPS) ≥ 1, and five (11.6%) had a CPS ≥ 10. No patients had Epstein–Barr virus positivity or mismatch repair deficiency.

**Table 1:** Baseline patient characteristics (*n* = 43)

	*N* (%)
Median Age (years, range)	63 (34–82)
**Sex**	
Male	33 (76.7%)
Female	10 (23.3%)
**Previous gastrectomy performed with curative intent**	
Yes	12 (27.9%)
No	31 (72.1%)
**ECOG performance status**	
0	26 (60.5%)
1	17 (39.5%)
**Pathology**	
AWD	2 (4.7%)
AMD	27 (62.8%)
APD	13 (30.2%)
SRC	1 (2.3%)
**Metastasized organs**	
Lymph node	36 (83.7%)
Liver	21 (48.8%)
Peritoneum	11 (25.6%)
Lung	7 (16.3%)
Bone	3 (7.0%)
Adrenal gland	1 (2.3%)
**HER2 positivity**	
IHC 3+	30 (69.8%)
IHC 2+ and SISH +	13 (30.2%)
**Pretreatment PD-L1 status**	
CPS < 1 (negative)	17 (39.5%)
CPS ≥ 1 (positive)	21 (48.8%)
CPS ≥ 10	5 (11.6%)
Not assessed	5 (11.6%)
**Baseline CEA**^a^	
<5 ng/mL	15 (34.9%)
≥5 ng/mL	28 (65.1%)
**Baseline CA 19-9**^a,b^	
<34 U/mL	19 (46.3%)
≥34 U/mL	22 (53.7%)
**EBV positivity**^c^	0 (0%)
**Deficient MMR**^d^	0 (0%)

*ECOG* Eastern Cooperative Oncology Group; *AWD* adenocarcinoma well differentiated; *AMD* adenocarcinoma moderately differentiated; *APD* adenocarcinoma poorly differentiated; *SRC* signet ring cell; *HER2* human epidermal receptor 2; *IHC* immunohistochemistry; *SISH* silver in situ hybridization; *CPS* combined positive score; *CEA* carcinoembryonic antigen; *CA* carbohydrate antigen; *EBV* Epstein–Barr virus; *MMR* mismatch repair.

^a^Tumor markers are grouped by the upper limit of the normal range.

^b^Data available for 41 of 43 patients.

^c^EBV positivity was determined based on EBV-encoded small RNA in in situ hybridization.

^d^Deficient MMR by immunohistochemistry for MLH1, MSH2, PMS2, and MSH6.

### Primary endpoints

No dose-limiting or unexpected toxicity was observed for the starting dose in phase Ib. Thus, the recommended phase II dose (RP2D), primary endpoint for phase Ib part, was selected as the starting dose for phase II, and all three patients in the phase Ib cohort were included in the final analyses for phase II. At the time of data cutoff on August 31, 2020, the median follow-up duration was 18.2 months (95% confidence interval [CI]: 16.5–23.1). Three patients continued the treatment, and seven (16.3%) completed the 2-year treatment without progression. The confirmed objective response rate (ORR) was 76.7% (95% CI: 61.4–88.2), achieving the primary endpoint for the phase II part, with six (14.0%) patients exhibiting complete response (CR) and 27 (62.8%) exhibiting partial response (PR) (Supplementary Table 1).

### Secondary endpoints: efficacy

**Fig. 2:**
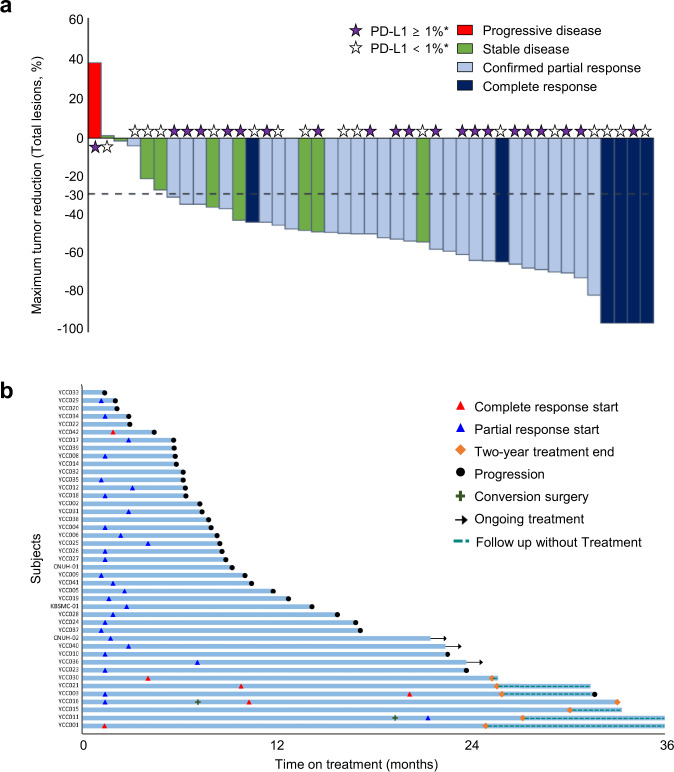
Tumor reduction in total lesions and tumor response. **a** Maximum percentage change from baseline in the size of total (target + non-target) tumor lesions with the corresponding best responses based on RECIST 1.1 criteria and the PD-L1 CPS from baseline tissue in all patients. The lower dotted line represents a 30% tumor reduction (*n* = 43). **b** A swimmer plot showing outcomes in all patients from the start of treatment to either disease progression or the last follow-up (*n* = 43). Note that seven patients received treatment for 2 years. CPS, combined positive score; PD-L1, programmed death-ligand 1; RECIST, Response Evaluation Criteria in Solid Tumors.

The disease control rate (DCR) was 97.7%, and nine (20.9%) patients had stable disease. Thirty-seven (86.0%) patients achieved a 30% reduction in total tumor burden (sum of target lesions and non-target lesions), while 26 (56.6%) patients achieved a 50% reduction (Fig. 2a). The duration and timing of objective responses are shown in Fig. 2b. Among responders, the median time to response was 1.7 (range, 1.2–5.8) months, and the median duration of response was 10.8 months (95% CI: 7.2–21.8). Two patients who achieved CR and PR as the best response underwent curative surgery following remarkable tumor shrinkage. Both patients completed 2-year treatment without progression, and only one patient showed new lesion progression approximately 6 months after finishing 2 years of treatment. An initial decrease in tumor burden from baseline was observed in all but one patient, suggesting a tumor shrinkage rate of 98% (Supplementary Fig. 1). Eight patients developed progression by the appearance of new lesions that were not seen before.

**Fig. 3:**
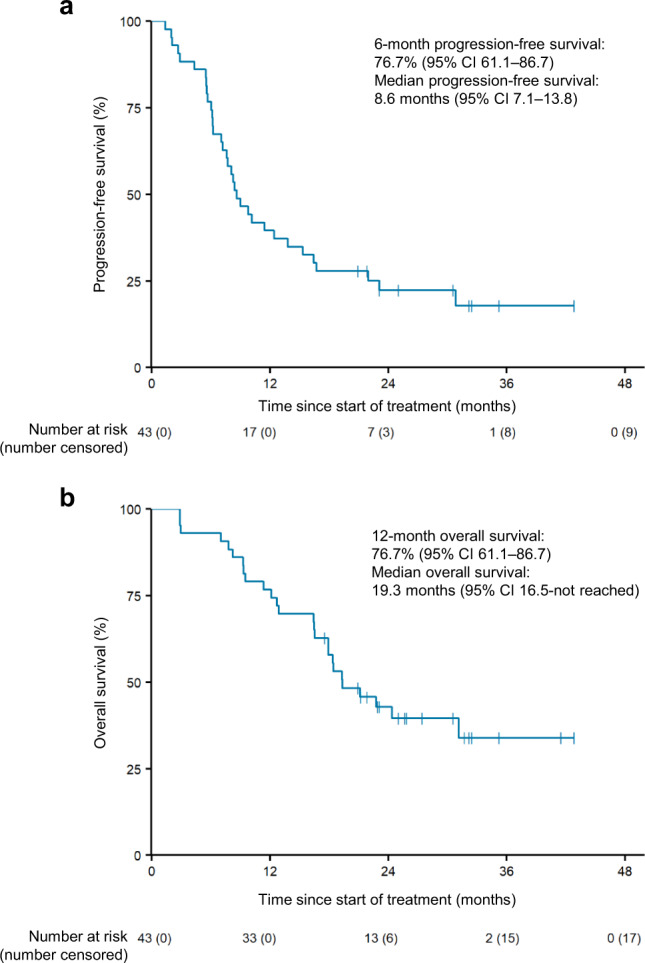
Progression-free survival and overall survival. **a** Kaplan–Meier curves of progression-free survival for all patients. **b** Kaplan–Meier curves of overall survival for all patients. Crosses denote censored observation, and number at risk is indicated below the plots.

The median PFS was 8.6 (95% CI: 7.2–16.4) months, with a 6-month PFS rate of 79.1% and a 1-year PFS rate of 41.9% (Fig. 3a). The median OS was 19.3 months (95% CI: 16.5 months–not reached [NR]), with a 1-year OS rate of 80.1% (Fig. 3b). The median number of treatment cycles was 12 (interquartile range [IQR], 8–24). The median numbers of capecitabine and cisplatin cycles were 8 (IQR, 6–12) and 6 (IQR, 4–7), respectively. Thirty-five patients continued treatment beyond the sixth cycle, and the median number of maintenance cycles was six (IQR, 2–12), with a median duration of 3.9 (95% CI: 2.13–11.57) months (Supplementary Table 2, Supplementary Fig. 2). No patients underwent platinum re-challenge after withdrawal or discontinuation. The maximum degree of tumor shrinkage was observed in the liver and peritoneum (mean, 62.5%), and one-third of liver lesions eventually progressed (*n* = 11 of 32, 34.4%) (Supplementary Fig. 3). There was no association between survival (PFS or OS) and pretreatment PD-L1 status, pretreatment neutrophil-to-lymphocyte ratio, or baseline tumor size (Supplementary Fig. 4).

### Secondary endpoints: safety

Treatment-related adverse events occurred in 42 patients (97.7%) (Table 2). Non-hematologic adverse events were mostly graded 1 or 2, and the commonly reported grade 3 adverse events were mostly hematologic, including neutropenia (39.5%), anemia (16.3%), febrile neutropenia (9.3%), and thrombocytopenia (7%). Grade 4 adverse events occurred in two patients (neutropenia and thromboembolic event). One patient died from grade 5 pneumonia related to disease progression. Thirty-nine (90.7%) patients required one or more reductions in the dose of capecitabine or cisplatin. Nine (20.9%) patients discontinued capecitabine due to adverse events, although treatment was resumed in three after the resolution of toxicity. Seven (16.3%) patients discontinued cisplatin due to adverse events, two of whom resumed treatment after the resolution of toxicity. None of the treatment-related adverse events led to the discontinuation of trastuzumab. Immune-related adverse events occurred in 16 patients (37.2%), but none required pembrolizumab discontinuation. Grade 3 immune-related adverse events included colitis (3/43, 7%) and allergic reaction (1/43, 2.3%).

**Table 2:** Treatment-related adverse events (TRAEs)

	Any Grade		Grade 3		Grade 4	
Event	*N*	%	*N*	%	*N*	%
Any TRAEs	42	97.7	33	76.7	2	4.7
**Hematologic**						
Neutrophil count decreased	20	46.5	17	39.5	1	2.3
Anemia	14	32.6	7	16.3	0	0
Platelet count decreased	7	16.3	3	7	0	0
Febrile neutropenia	5	11.6	4	9.3	0	0
**Non-hematologic**						
Anorexia	17	39.5	2	4.7	0	0
Nausea	14	32.6	0	0	0	0
Creatinine increased	13	30.2	2	4.7	0	0
Diarrhea	13	30.2	1	2.3	0	0
Hand-foot syndrome	10	23.3	0	0	0	0
Oral mucositis	10	23.3	1	2.3	0	0
General weakness	8	18.6	1	2.3	0	0
Peripheral neuropathy	8	18.6	0	0	0	0
Abdominal pain	6	14	0	0	0	0
Fever	6	14	0	0	0	0
Hypoalbuminemia	2	4.7	1	2.3	0	0
Hyperkalemia	2	4.7	2	4.7	0	0
Tinnitus	2	4.7	0	0	0	0
Thromboembolic event	2	4.7	0	0	1	2.3
**Immune-related**						
Hypothyroidism	5	11.6	0	0	0	0
Allergic reaction	4	9.3	1	2.3	0	0
Pruritus	4	9.3	0	0	0	0
Colitis	3	7	3	7	0	0
Adrenal insufficiency	2	4.7	0	0	0	0
Hyperglycemia	2	4.7	0	0	0	0
Skin rash	2	4.7	0	0	0	0

One patient died from Grade 5 pneumonia related to disease progression.

### Post hoc genomic analyses

**Fig. 4:**
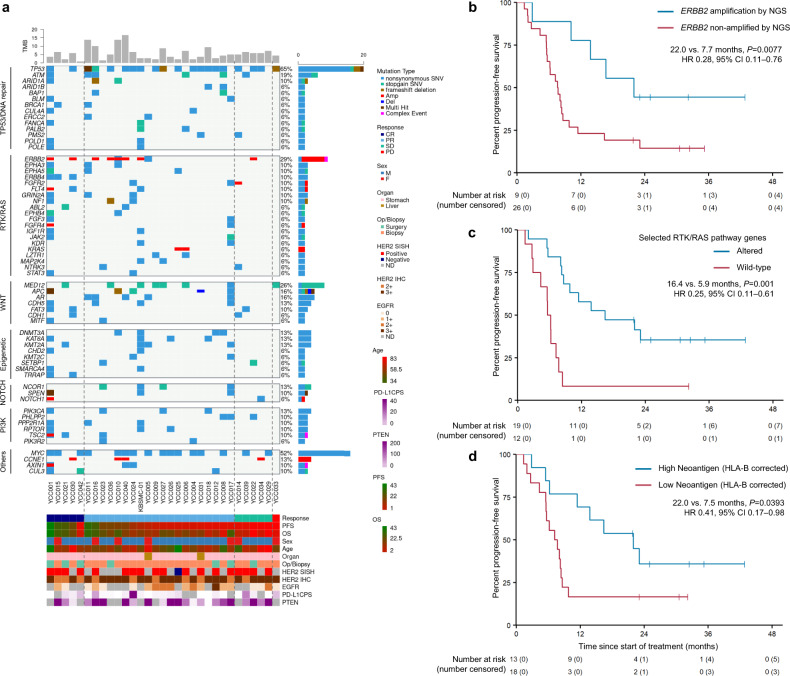
Baseline genomic landscape in the enrolled patients with HER2-positive gastric cancer treated with the first-line quadruplet regimen. **a** Baseline (pretreatment) tumor tissue-targeted DNA sequencing results grouped by the best response and related clinicopathologic features (*n* = 31). Curated pathways and selected genes altered in 6% or more of the patients are shown. Other pathways included those related to the cell cycle and the Hippo, MYC, and NRF2 pathways. Vertical dashed lines indicate groups by the best response. **b**–**d** Kaplan–Meier survival curves with PFS stratified by pretreatment *ERBB2* amplification as determined via NGS (**b** *n* = 35), RTK-RAS pathway gene alterations (**c** *n* = 31), and HLA-B-corrected neoantigen load (**d** *n* = 31). In (**b**–**d**), two-sided *P*-values for survival associations were calculated using the log-rank tests. Hazard ratios and corresponding 95% CIs were estimated using Cox proportional hazard regression model. No adjustments for multiple comparisons were made. Crosses denote censored observation, and the number at risk is indicated below the plots. HER2, human epidermal receptor 2; ERBB2, Erb-B2 receptor tyrosine kinase 2; HLA, human leukocyte antigen; NGS, next-generation sequencing; PFS, progression-free survival; RTK, receptor tyrosine kinase.

**Fig. 5:**
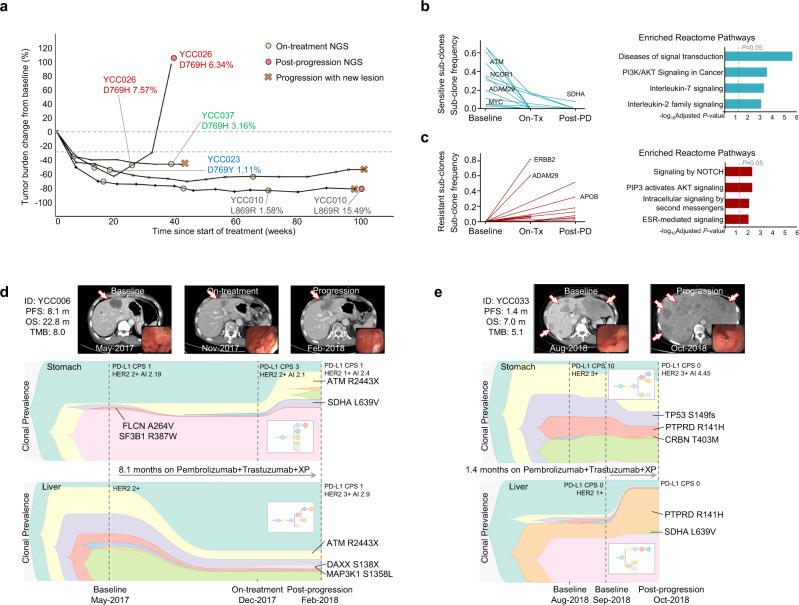
Genomic analyses from serial biopsy samples of HER2-positive gastric cancer patients treated with the first-line quadruplet regimen. **a** Spider plot showing patients with HER2 mutations found in serial NGS analyses of primary tumor. Only cases with HER2 mutations and variant allele frequencies (VAFs) with the corresponding patient IDs are shown. **b**, **c** Sensitive (**b**) or resistant (**c**) sub-clones in which the sub-clone frequency changed over 2-fold in post-progression (Post-PD) samples (*n* = 10) or on-treatment (On-Tx) samples (*n* = 9) compared to paired baseline samples (*n* = 14) are selected per patient. Statistically significant enriched Reactome pathways with the multiple test correction (Benjamini-Hochberg procedure) were retained, and selected enriched Reactome pathways from sensitive or resistant sub-clone genes are also shown. All paired tissue samples are only from the primary tumor (stomach). Only hotspot gene mutations that appeared in more than two samples are shown in the sub-clone frequency graph. X-axis indicated –log10 adjusted *P*-values and statistical significance is indicated by the vertical dashed line. **d**, **e** Representative cases showing sub-clonal evolution by fish plot and corresponding clinicopathologic features and computed tomography or endoscopic images from paired tissue NGS from both primary tumor (stomach) and metastatic liver. Selected hotspot mutations are labeled. Representative case from good responders (*n* = 11, **d**) and poor responder (*n* = 1, **e**). HER2, human epidermal receptor 2; ERBB2, Erb-B2 receptor tyrosine kinase 2; NGS, next generation sequencing.

Primary or metastatic tissues from 39 of 43 patients were subjected to *post hoc* analyses via targeted next-generation sequencing (NGS) (Supplementary Table 3). The NGS results of 72 samples (38 patients) were included in the final analyses (Supplementary Fig. 5). Baseline (pretreatment) tissues from 31 patients were available for NGS (primary tumors, 29; liver metastases, 2) (Fig. 4a). No patients exhibited altered c-*MET* expression, and PD-L1 expression was not related to the genomic alteration pattern. Erb-B2 Receptor Tyrosine Kinase 2 (*ERBB2*) amplification was detected in 10 patients (23.3%), nine of whom were HER2-positive based on IHC 3+ (Supplementary Table 4). Statistically significant differences were not observed between PFS and OS in patients with IHC 3+ (*n* = 30) and those with IHC 2+ and SISH positivity *(n* = 13). However, patients with *ERBB2* amplification (*n* = 10) exhibited longer PFS (22.0 vs. 7.6 months, hazard ratio [HR]: 0.28, 95% CI: 0.11–0.76, *P* = 0.012) and OS (NR vs. 17.9 months, HR: 0.18, 95% CI: 0.04–0.79, *P* = 0.0228) than those without *ERBB2* amplification (*n* = 25) (Fig. 4b). Interestingly, patients with pretreatment *ERBB2* amplification were mostly long-term responders (PFS over 12 months) (Supplementary Fig. 6). De novo genetic alterations in the RTK/RAS pathway (excluding *ERBB2* amplification/alterations and deletion of *RTK*) were observed in 61.3% of patients (*n* = 19, Supplementary Fig. 7, Supplementary Table 5). Patients with these genetic alterations exhibited longer PFS (16.4 vs. 5.9 months, HR: 0.25, 95% CI: 0.11–0.61, *P* = 0.001) and OS (31.2 vs. 11.8 months, HR: 0.24, 95% CI: 0.10–0.61, *P* = 0.001) than those without alterations (Fig. 4c, Supplementary Fig. 7). In contrast, among patients with HER2-positive AGC treated with first-line trastuzumab, capecitabine, and cisplatin in our retrospective cohort (*n* = 18), no statistically significant differences were observed between the prognosis of patients with RTK/RAS pathway alterations (*n* = 7) and those without alterations (Supplementary Fig. 7, Supplementary Table 6). Patients with a high human leukocyte antigen B (HLA-B)-corrected neoantigen load (>median) exhibited better PFS (22.0 vs. 7.5 months, HR: 0.41, 95% CI: 0.17–0.99, *P* = 0.0393) and OS (NR vs. 17.4 months, HR: 0.32, 95% CI: 0.11–0.92, *P* = 0.0263) than those with a low neoantigen load (Fig. 4d, Supplementary Fig. 8). In addition, HLA homozygosity, subtle HLA supertypes, and alleles (HLA-B44 and HLA-B*15:01) predictive of immunotherapy response in a previous study^12^ were also analyzed; however, statistical significance was not observed for survival (Supplementary Fig. 9). A high tumor mutational burden (TMB) (defined as >10 mut/Mb) was detected in only four patients (TMB high vs. low; median PFS, NR vs. 8.1, *P* = 0.0409; median OS, NR vs. 18.4, *P* = 0.0973; Supplementary Fig. 10). Alterations in genes encoding proteins involved in the response to DNA damage were not related to survival (Supplementary Fig. 11). Multivariable analysis indicated that RTK/RAS pathway alterations were correlated with survival (PFS, HR: 0.24, 95% CI: 0.08–0.71, *P* = 0.01; OS, HR: 0.3, 95% CI: 0.1–0.93, *P* = 0.037) independent of HLA-B-corrected neoantigen load and *ERBB2* amplification (Supplementary Table 7). Biopsy samples obtained during treatment (*n* = 21 from 16 patients) and post-progression (*n* = 19 from 17 patients) were subjected to additional genomic studies. No patients exhibited significant *ERBB2* mutations at baseline NGS. In four patients, *ERBB2* mutations were detected during on-treatment or post-progression NGS (Fig. 5a). These mutations included D769H, D769Y, and L869R *ERBB2* alterations, which have been shown to impart resistance^13^. These *ERBB2* mutations suggest a possible mechanism of acquired resistance based on an increase in sub-clones (patients YCC010, YCC026, and YCC037), which supports the implementation of serial genomic studies upon re-biopsy. To analyze sub-clonal evolution within the primary tumor, paired NGS data for the stomach tissues of 14 patients were compared using either on-treatment samples (*n* = 9) or post-progression samples (*n* = 10). More than 20 genes were selected to identify hotspot mutations from potentially sensitive or resistant sub-clones, with no dominant gene alteration (Supplementary Table 8). Pathways associated with PI3K/AKT signaling, interleukin-7 signaling, and interleukin-2 signaling were related to sensitive sub-clones (Fig. 5b and Supplementary Table 9a), whereas NOTCH signaling and ESR-mediated signaling were related to resistant sub-clones (Fig. 5c and Supplementary Table 9b). Some patients had serial-biopsied tissues available from both the primary tumor (stomach) and metastatic site (liver). Serial comparison analyses among these patients showed spatial tumor heterogeneity between the primary and metastatic sites, along with longitudinal tumor heterogeneity throughout the treatment. The first representative case (best response: PR, Fig. 5d) showed that major sub-clones were decreased in the metastatic liver, and minor sub-clones appeared as progression occurred at the primary tumor site, which indicated acquired resistance. The second representative case (best response: PD, Fig. 5e) showed an increase in major sub-clones in the metastatic liver as the disease progressed, which indicated a rather primary resistance. Both cases showed clonal expansion as the disease progressed but without actionable hotspot mutation.

## Discussion

In this study, patients with HER2-positive AGC were treated with a first-line quadruplet regimen of pembrolizumab, trastuzumab, capecitabine, and cisplatin. Absolute survival (median PFS: 8.6 months; median OS: 19.3 months) was longer than that in the ToGA trial^3^ (median PFS: 6.7 months; median OS: 13.8 months for the primary population and 16.0 months for IHC 2 + /FISH positive or IHC 3 + ), possibly due to the addition of pembrolizumab. The quadruplet regimen was tolerable and exhibited comparable safety to that previously reported for first-line trastuzumab plus chemotherapy^3^ or a combination of pembrolizumab, trastuzumab, and chemotherapy^8^. Our results suggest that adding pembrolizumab to trastuzumab plus chemotherapy, an existing first-line standard therapy, is safe and effective in patients with advanced HER2-positive gastric cancer.

Recently reported trials have also demonstrated the benefits and safety of adding pembrolizumab to trastuzumab and chemotherapy as a first-line treatment for patients with HER2-positive gastric cancer. Our study, conducted in Asian patients only, indicates that the quadruplet regimen exhibits overall efficacy and safety concordant with the results of a recently reported phase II trial^8^. However, the previous trial^8^ differed from ours in that it included patients with esophageal adenocarcinoma (38%) and non-Asian patients (95%), and more than half of the patients were treated with an induction phase (1 cycle) of pembrolizumab and trastuzumab alone without cytotoxic chemotherapy. In addition, most patients were treated with cytotoxic chemotherapy consisting of oxaliplatin (97%), whereas we used the same chemotherapy backbone (capecitabine plus cisplatin) and dose administration schedule as the ToGA trial. Treatment-related adverse events leading to permanent drug discontinuation occurred in only 5% of patients in the previously reported study, compared with 20.9% discontinuation of capecitabine and 16.3% discontinuation of cisplatin in the present study. The ongoing randomized phase III Keynote-811 study^9,14^ follows the same dosing schedule with a capecitabine/oxaliplatin or 5-FU/cisplatin chemotherapy backbone in the same patient population as those in the PANTHERA trial. The interim ORR and DCR results achieved in the Keynote-811 study were similar (74.4% vs. 76.7% and 96.2% vs. 97.7%, respectively) to those in the PANTHERA trial. Thus, we expect that Keynote-811 study will also report improved survival when comparing the treatment with the trastuzumab plus doublet chemotherapy regimen used in the ToGA trial.

Although only patients with HER2 overexpression were enrolled, not all patients demonstrated *ERBB2* amplification. Reports regarding concordance between *ERBB2* amplification in NGS and HER2 expression in IHC/SISH remain controversial^15^. Patients with *ERBB2* amplification exhibited a better response to the quadruplet regimen and improved survival, whereas HER2 IHC status (2+ or 3+) was not related to survival. The pretreatment *HER2* SISH amplification index was correlated with *HER2* copy number (Supplementary Fig. 6), implying that the extent of *ERBB2* amplification is important for survival rather than the method of detection^16^. Each patient with *ERBB2* amplification determined via NGS was HER2 3+ based on IHC results. However, the *ERBB2* amplification index was analyzed via SISH in only three patients. Although not mandatory, determining *ERBB2* amplification status via NGS or SISH even for HER2 IHC 3+ cases may help to identify a subset of patients likely to exhibit improved response and survival outcomes, as demonstrated in another study^8^.

Patients with RTK/RAS pathway alterations exhibited a favorable prognosis, in contrast to previous findings indicating that RTK/RAS pathway genes may be involved in a bypass mechanism or induce resistance to anti-HER2 treatments^8,17^. One technical difference was that we used NGS to assess mutations with variance allele frequencies (VAFs) >1.0%, compared to VAF > 5.0% in the previous studies^8,17^. In addition, preclinical and clinical studies have reported that the RTK/RAS pathway is related to an enhanced response to immunotherapy via stabilization or overexpression of PD-L1^18,19^.

Although it is controversial whether PD-L1 status can be used to adequately predict response after combined chemo-immunotherapy^20^, TMB and HLA have been suggested as important predictors of response^12,21^. However, few patients in this study had high TMB (*n* = 4, 12.9%). As HLA is also considered important for predicting immunotherapy response^12,22–24^, we estimated neoantigen load by predicting the binding of each patient’s somatic mutation peptides to major histocompatibility complex (MHC) class I molecules using a deep learning algorithm. HLA-B-corrected neoantigen load, which may be important in the HLA-B-restricted T-cell response^25,26^, was related to PFS and OS. Thus, HLA-B-corrected neoantigen load may be important for predicting response to immunotherapy in patients with AGC.

We analyzed a relatively large number of paired biopsy samples in AGC patients treated with an immunotherapy-containing regimen. Sensitive sub-clones were characterized by genetic alterations in pathways associated with PI3K/AKT, interleukin-7, and interleukin-2. The mutation sites in these genes were mostly in regions encoding non-kinase domains, suggesting that these mutations act by increasing antigenicity for immunotherapy rather than inducing direct resistance to HER2-targeted treatment. Furthermore, in the sensitive sub-clones, signaling pathways associated with cytokines were enriched, indicating their role in cancer immunotherapy (Supplementary Table 9). Pathways associated with NOTCH, PIP3, and ESR were enriched in resistant sub-clones, and further investigations regarding their roles in the mechanisms of treatment resistance are warranted. When patients were grouped according to the mechanism of acquired resistance (acquired *HER2* mutations and emergence of resistant sub-clones), none exhibited all two features, suggesting that mechanisms of acquired resistance to the quadruplet regimen vary from patient to patient. Further larger studies are required to support the clinical utility of serial genomic studies upon re-biopsy.

This study had some limitations, including its single-arm design, the small sample size, lack of immune cell profiling due to scarcity of available tissues, lack of statistical consideration for exploratory molecular analyses, and an inadequate number of tumors for baseline NGS. The lack of statistically significant associations of biomarkers can be due to the small sample size. Despite these limitations, our biomarker study results can provide avenues for further research and insights that can be used to develop biomarker or functional studies and to stratify factor design in future confirmatory phase III trials. Further studies are required to verify our findings and address these issues.

In conclusion, first-line therapy using a quadruplet regimen led to tumor shrinkage in HER2-positive AGC. The proposed correlative biomarkers and molecular mechanisms possibly underlying the response to the quadruplet regimen observed in our study can be validated in the ongoing phase III Keynote-811 study.

## Methods

The trial was conducted in accordance with the Declaration of Helsinki and the Guidelines for Good Clinical Practice (ClinicalTrials.gov identifier: NCT02901301). The trial protocol was approved by the Institutional Review Board of Severance Hospital (Seoul, South Korea), Chonnam National University Hwasun Hospital (Jeollanam-do, South Korea), Kangbuk Samsung Hospital (Seoul, South Korea), Gangnam Severance Hospital (Seoul, South Korea), and Hallym University Medical Center (Anyang, South Korea). All participants provided written informed consent before the enrollment.

### Design and participants

The PANTHERA trial was an open-label, phase Ib/II trial performed at five academic cancer centers in South Korea to evaluate the efficacy and safety of a quadruplet regimen (pembrolizumab, trastuzumab, capecitabine, and cisplatin) as a first-line therapy for HER2-positive advanced gastric cancer.

Eligible patients were men and women aged at least 19 years or older and had HER2-positive (defined as either IHC 3+ or IHC 2+ in combination with ISH + , as assessed by a local laboratory on primary or metastatic tumor tissues) advanced gastroesophageal junction or gastric adenocarcinoma. Other major inclusion criteria included previously untreated advanced metastatic gastric cancer (patients treated with adjuvant therapy were allowed if therapy had been completed more than six months before study enrollment) with measurable disease based on RECIST (Response Evaluation Criteria In Solid Tumors) version 1.1, performance status of 0 or 1 based on the Eastern Cooperative Oncology Group (ECOG) Performance status, adequate organ function including cardiac function, and for female subjects of childbearing age, negative urine or serum pregnancy test result or the willingness to use birth control during the study. Key exclusion criteria were currently participating and receiving study therapy or having participated in a study of an investigational agent within four weeks of the first dose of treatment, immunodeficiency or receiving systemic steroid therapy or any other form of immunosuppressive therapy within seven days prior to the first dose of trial treatment (physiologic dose of systemic steroid was permitted), known history of active tuberculosis, hypersensitivity to pembrolizumab or any of its excipients, non-recovery (i.e., ≤Grade 1 or at baseline) from adverse events due to a previously administered agent, known additional malignancy that is progressing or requires active treatment within 3 years, except basal cell carcinoma or squamous cell carcinoma of the skin, in situ cervical cancer, thyroid cancer subjected to curative therapy, active central nervous system metastases and/or carcinomatous meningitis, active autoimmune disease subjected to systemic treatment in the past two years, known history or any evidence of active, non-infectious pneumonitis, active infection requiring systemic therapy, known psychiatric or substance abuse disorders that would interfere with cooperation during the trial, pregnant or breastfeeding state, or expecting to conceive or father children within the projected duration of the trial, starting with the pre-screening or screening visit through 120 days after the last dose of trial treatment, prior therapy with an anti-PD-1, anti-PD-L1, or anti-PD-L2 agent, known history of Human Immunodeficiency Virus (HIV) (HIV 1/2 antibodies), Hepatitis B (HBsAg reactive and HBV DNA detected) or Hepatitis C (anti-HCV reactive and HCV RNA [qualitative] detected), and a live vaccine recipient within 30 days of the planned start of the trial.

Conversion surgery—defined as surgical treatment with a curative intent performed after tumors initially deemed technically or oncologically unresectable respond to therapy—was permitted, and PFS was censored at the time of conversion surgery. Between February 6, 2017 (first patient enrolled) and March 27, 2019 (the last patient enrolled), 43 patients were enrolled.

For the retrospective database cohort (Supplementary Table 6), HER2-positive AGC patients treated with first-line trastuzumab plus capecitabine and cisplatin who performed in-house panel sequencing (CancerMaster Panel V2) with pretreatment tumor tissues were retrospectively reviewed and analyzed.

### Procedures

For phase Ib (safety run-in period), we employed a 3 + 3 design with a starting dose (dose level 0) and the dose level −1. The definition of dose-limiting toxicity is described in Supplementary Methods. Dose level 0 was as follows: 200 mg intravenous pembrolizumab on day 1, 6 mg/kg intravenous trastuzumab for maintenance (Herzuma® [CT-P6], Celltrion Inc.) after an 8 mg/kg load on day 1, 1,000 mg/m^2^ oral capecitabine twice daily on days 1–14, and 80 mg/m^2^ intravenous cisplatin on day 1 every 3 weeks. For dose level −1, the cisplatin dose was reduced to 60 mg/m^2^. The RP2D from phase Ib was used in phase II.

All patients were treated until disease progression or unacceptable toxicity occurred, or until up to six cycles of capecitabine and cisplatin and up to 24 months of pembrolizumab and trastuzumab were received (chemotherapy beyond six cycles was permitted at the investigator’s discretion). Computed tomography or magnetic resonance imaging was performed within 28 days before the first dose (baseline). Tumor responses were evaluated by independent radiologists every 6 weeks using the RECIST criteria for 1 year and every 9 weeks thereafter. The left ventricular ejection fraction was measured using multi-gated acquisition scans or echocardiography every 9 weeks during the first eight cycles and every 12 weeks thereafter. Toxicity was graded based on the National Cancer Institute Common Terminology Criteria for Adverse Events 4.0. Dose reductions were allowed for capecitabine and cisplatin according to the type and severity of adverse events, but not for pembrolizumab or trastuzumab.

Tumor tissues obtained from either primary or metastatic tumors were subjected to in-house panel sequencing using CancerMaster Panel V2^27^ (Supplementary Table 3).

### Outcomes

The primary endpoint of the phase Ib study was to assess the recommended phase II dose (RP2D). Dose-limiting toxicity (DLT) was observed during the first cycle. DLT included hematologic DLTs (grade 4 neutropenia lasting ≥7 days; grade 4 neutropenia with fever >38.5°C, and/or infection requiring antibiotic or anti-fungal treatment; grade 4 thrombocytopenia) or non-hematologic DLTs (grade 3 or greater toxicity lasting >48 h other than anorexia, nausea, diarrhea, and alopecia despite appropriate supportive care). The primary efficacy endpoint of the phase II study was to evaluate the anti-tumor activity of the quadruplet regimen in subjects with AGC. Objective response rates per RECIST 1.1 were to be used as the primary response rate efficacy endpoint. The secondary endpoints included PFS, OS, duration of response, time to response, and safety. ORR was calculated as the percentage of patients who achieved a confirmed CR or PR. DCR was defined as the proportion of subjects with confirmed CR, PR, and stable disease, for at least 4 weeks. PFS was defined as the time from treatment initiation until the date of disease progression or death from any cause. For patients who had no disease progression or who did not die, the censoring date was defined as the last date at the nearest time of their last response evaluation. OS was defined as the time from treatment initiation to the date of death from any cause. OS censoring date was defined as the last date that the subject was known to be alive as of the data cut-off date for the analysis. Time to response was defined as the time from treatment initiation to the date of first response (CR or PR).

### Clinicopathologic analyses

We collected data related to the following variables: age at the beginning of treatment; sex; Eastern Cooperative Oncology Group performance status; pathology; plasma levels of albumin, lactate dehydrogenase (LDH), carcinoembryonic antigen, and carbohydrate antigen 19-9; neutrophil and lymphocyte counts; neutrophil-to-lymphocyte ratio (NLR); and metastatic organs. Parameters previously reported to be related to immunotherapy response – LDH (LDH > upper limit of normal), albumin (albumin <3.5 mg/dL), NLR (NLR > 6), and baseline tumor size (baseline tumor size > median) – were post-hoc analyzed^28,29^.

### Histological analyses

Tumor tissues were fixed in 10% formalin, embedded in paraffin, and cut into 4 μm-thick tissue sections for further analyses. Immunohistochemistry (IHC) staining was performed using the Ventana Benchmark XT automated staining system (Ventana Medical Systems, Tucson, AZ, USA) according to the manufacturer’s protocol.

For assessing human epidermal receptor 2 (HER2) status, anti-HER2/neu antibody (Clone 4B5; ready to use; Ventana Medical Systems) was used for IHC, and HER2 expression scoring system was applied according to the guideline^30^. In addition, HER2 DNA amplification was evaluated using silver in situ hybridization (SISH). SISH has been introduced as an alternative to FISH and combines the accuracy of FISH with the use of light opaque silver instead of fluorescent spot-like signals. As SISH can be performed more rapidly than FISH and requires only a conventional light microscope, it is preferred for routine use in pathology laboratories^31,32^. SISH was performed with INFORM® *HER2* DNA and chromosome 17 (*CEP17*) probes (Ventana Medical Systems) using a Ventana Benchmark XT automated staining system, in accordance with the manufacturer’s instructions. *HER2* DNA amplification was defined as HER2/CEP17 ratio of ≥2.0.

Epstein–Barr virus (EBV) status was assessed using EBV-encoded small RNA in situ hybridization using standard protocols. The INFORM® EBER Probe (Ventana Medical Systems, Tucson, USA) was used to perform automated staining, according to the manufacturer’s instructions. Positive staining was defined by diffuse staining of tumor cells.

Tumor tissue microsatellite instability status was determined using IHC for MutL homolog 1 (MLH1; Clone M1; ready to use; Ventana Medical Systems), MutS protein homolog 2 (MSH2; clone G219-1129; ready to use; Cell Marque, Rocklin, CA, USA), postmeiotic segregation increased 2 (PMS2; clone MRQ-28; 1:40; Cell Marque), and MutS homolog 6 (MSH6; Clone 44; 1:100; Cell Marque) in formalin-fixed paraffin-embedded (FFPE) tissue sections. Loss of staining was defined as complete loss of nuclear staining in all the tumor nuclei with preserved staining of lymphocytes and/or non-neoplastic gastric foveolar epithelium.

For programmed death-ligand 1 (PD-L1) status, IHC staining was carried out using Dako PD-L1 IHC 22C3 pharmDx kit (Agilent, Santa Clara, CA, USA) with EnVision FLEX visualization system and counterstained with hematoxylin according to the manufacturer’s instructions. PD-L1 protein expression was determined using a combined positive score defined as the number of PD-L1 staining cells (tumor cells, lymphocytes, and macrophages) divided by the total number of viable tumor cells.

Antibodies recognizing epidermal growth factor receptor (1:100, EP38Y, Abcam, Cambridge, UK), PTEN (1:100, clone 138G6, Cell Signaling Technology, Danvers, MA, USA) were also used for IHC, as previously described^33^.

### Tumor sample collection and in-house next-generation sequencing (NGS)

Tumor tissues, either from primary or metastatic tumors, were obtained. Of the quality-controlled (QC) samples, 98 were subjected to in-house panel sequencing using CancerMaster Panel V2^27^. The CancerMaster Panel V2 covers 524 genes for single nucleotide variants (SNVs), 143 for copy-number variations, and 18 for fusions. Tumor DNA was extracted from freshly obtained tissues or FFPE archival tissues using a QIAamp DNA FFPE Tissue Kit (Qiagen, Germany) according to the manufacturer’s instructions. We determined DNA Integrity Number (DIN), measured using 4200 Tapestation (Agilent), as <3.5, or sheared DNA ratio (ratio of total amount of DNA after shearing, divided by initial sample DNA amount) as <0.4 for the QC cutoff. Sequencing libraries were prepared using the Celemics Library Preparation Kit (Celemics). To capture all target regions, NGS libraries and capture probes were hybridized using Celemics Targeted Sequencing Kit (Celemics). Pooled libraries containing captured DNA fragments were subsequently sequenced on Illumina NextSeq 500 Sequencing System as 2 × 100 bp paired-end reads.

Sequencing reads were processed by our in-house bioinformatics pipeline following the Genome Analysis Toolkit (GATK) best practices workflows for somatic short variant discovery or the recommended best practices for the Illumina system. Paired-end reads were mapped to the reference genome (GRCh37/hg19) using BWA (v0.7.10). Removal of duplicate reads and base quality recalibration were processed using Picard (v1.115). SNVs and small insertion and deletions (INDELs) were detected using VarScan 2.4.0 and GATK IndelRealigner 2.3.9 with default parameters. The following quality criteria were applied to the detected variant: total depth ≥200, alter read depth ≥2 and strandRatio ≤0.9, variant allele frequency (VAF) of SNV ≥ 1%, VAF of InDel ≥10%, and no strand bias. Manual curations from the annotation databases COSMIC v91, OncoKB, ClinVar, ANNOVAR, and literature analysis were also performed by expert reviewers, and clinically relevant mutations were additionally included. Copy number alterations were identified by comparing the depth of coverages over targeted regions in a tumor sample, relative to a reconstructed baseline. For internal normalization of sequencing depth variation, regional depth was divided by the median depth of each sample. For evaluating copy number alteration, we used segmental copy number, which was calculated using a circular binary segmentation (CBS) algorithm with R package ‘DNAcopy’. Copy gain or loss was defined if the segmental relative value was higher than 3.5 or lower than 0.5, genic fold change over 4 or lower than 0.5, and also at least more than three regions had the same events. Fusion genes were identified with TopHat-Fusion 2.0.13, and fusions with more than 5 spanning reads and a fusion score higher than 0 were reported. We used SOAP-HLA (v2.2) for typing of human leukocyte antigen (HLA) and reported types with scores higher than 50. For patients in whom in-house NGS was not performed, but pre-treatment FoundationOne CDx assay was done, *ERBB2* (Erb-B2 receptor tyrosine kinase 2) amplification status from the reports was used.

### Tissue genomic analyses

The tumor mutation burden (TMB; mutations [mut]/megabase [Mb]) was estimated as the total number of detected nonsynonymous mutations (SNP VAF > 5% and indel VAF > 10%; putative germline mutations reported in population databases [Korean Variant Archive^34^, Korean Reference Genome Database^35^, The Exome Aggregation Consortium (East Asian)^36^, 1,000 Genomes Phase 3 (East Asian)]^37^ were removed) divided by the length of the covered coding regions. A TMB value over 10 mut/Mb was considered as TMB-high^21^. HLA-corrected neoantigen load was calculated according to the previous report^24^. Briefly, a pre-constructed convolutional neural network (CNN) model was used to predict the binding of HLA-A or HLA-B molecules and mutated genes detected from in-house panel sequencing. The interactions between 9-mer peptide sequences containing altered amino acids of mutated genes and HLA sequence were projected into a 365 ×9 input matrix and the kernel of the CNN model detected a specific binding pattern having high interaction preference^38^ in amino acid level with positional effect. Patients having HLA-corrected neoantigen load more than the median value were predicted to have a high neoantigen (13 for HLA-A and 4 for HLA-B). The genomic landscape plot (oncoplots, Fig. 4a) was generated using the R package ‘maftools’^39^. Signaling pathways were annotated manually according to previously reported curated oncogenic signaling pathways^40^. For the receptor tyrosine kinase (RTK)/RAS pathway alteration group, *ERBB2* gene alteration (amplification and mutations) and *RTK* gene deletions were excluded. The functional effects of mutations were reported from the Catalogue of Somatic Mutations in Cancer (COSMIC)^41^ database and were predicted by Functional Analysis Through Hidden Markov Models - Multiple Kernel Learning (FATHMM-MKL) algorithm^42^; clinical significances were defined according to ClinVar or American College of Medical Genetics (ACMG) criteria using tool for assessment and prioritization in exome studies (TAPES)^43^ (Supplementary Table 5). For patients in whom in-house next-generation sequencing (NGS) was not performed, but pretreatment FoundationOne CDx assay was done, *ERBB2* amplification status from the reports was used.

A DNA damage response (DDR)-related gene list was assembled^44–46^. Patients were classified into DDR alteration group or wild-type group according to the results of targeted sequencing. A patient was assigned to DDR alteration group if a homozygous deletion or deleterious mutation was detected in DDR-related genes.

For the analyses of sub-clonal evolution in serial-biopsied samples, PyClone^47^ and clonality inference in tumors using phylogeny (CITUP) were used^48^. We used PyClone to obtain the cellular prevalence of serial-biopsied samples (mutations located on the X or Y chromosomes were removed), and the information of cellular prevalence was used as an input for CITUP to infer phylogenetic trees. R packages ‘timescape’ and ‘fishplot’ were used for visualization. To determine resistant or sensitive sub-clones, NGS results from baseline tissues were compared with those from on-treatment or post-progression samples. Sub-clone frequencies that changed over 2-fold at post-progression or on-treatment samples compared to baseline samples were regarded as sensitive (decrement) or resistant (increment) sub-clones (when both on-treatment and post-progression samples were available, only the sub-clones with continuous decreases or increases during treatment were selected). Biological pathways, enriched by chosen genes as sensitive or resistant sub-clones, were annotated from the Reactome database (https://reactome.org) using the R package ‘ReactomePA’^49^.

### Statistics & reproducibility

The size of the dose de-escalation cohort was determined using a conventional 3 + 3 design. According to Simon’s two-stage minimax design^50^, a minimum sample size of 38 patients was required to accept the hypothesis that the true response rate was 60% with 80% power and to reject the hypothesis that the response rate was less than 45% (adopted from ORR of the experimental arm of ToGA trial; 47.3%), with a type I error of 0.2. The study was to be stopped if fewer than six responses were observed among the initial 13 patients in the first stage. Patients who completed at least one treatment cycle were considered evaluable for the primary outcome and safety assessments. The duration of response was analyzed using *post hoc* tests for patients who exhibited the best response in terms of either CR or PR and was defined as the time of best response (whichever occurred first) until the date of progression. OS, PFS, and duration of response were estimated using the Kaplan–Meier method. The proportion of patients who achieved ORR was estimated using binomial proportions and 95% CIs.

All analyses were performed using R 4.0.3 (R project; The R Foundation for Statistical Computing, Vienna, Austria) or GraphPad Prism version 8 (GraphPad Software, San Diego, CA, USA). Survival curves were plotted and compared using the log-rank test. Cox proportional hazards regression models were used for univariate and multivariable analyses. Data were compared using the two-tailed, unpaired *t*-test for two groups or the one-way analysis of variance followed by Tukey’s multiple comparison test for multiple groups unless otherwise noted. The Benjamini–Hochberg procedure was used to control the false discovery rate. *P* < 0.05 was considered statistically significant.

## Supplementary information

Supplementary Information File

## Data Availability

The raw clinical and imaging data are protected due to patient privacy laws. The datasets generated during and/or analyzed during the current study are available from the corresponding author Hyun Cheol Chung on request for 10 years; de-identified clinical data and experimental data are available on request sharing, which will need the approval of the institutional ethical committees. De-identified data will then be transferred to the inquiring investigator over secure file transfer. Targeted panel sequencing data generated in this study have been deposited in the NCBI database under SRA accession codes PRJNA822021 and PRJNA873211. The functional effects of mutations were reported from the Catalogue of Somatic Mutations in Cancer (COSMIC) database^51^. The study protocol is available as Supplementary Note in the Supplementary Information file. The remaining data are available within the Article, Supplementary Information, or Source Data file. Source data are provided with this paper.

## Source data

Source Data
