# Supplementary material for: A single arm phase Ib/II trial of first-line pembrolizumab, trastuzumab and chemotherapy for advanced HER2-positive gastric cancer
Source: Nat Commun. 2022 Oct 12;13:6002. doi: 10.1038/s41467-022-33267-z (PMC9556512; doi:10.1038/s41467-022-33267-z)
Supplement: Supplementary file 1 — Supplementary Information File [file 41467_2022_33267_MOESM1_ESM.pdf]

Supplementary Information File

A single arm phase Ib/II trial of first-line pembrolizumab, trastuzumab and chemotherapy for advanced HER2-positive gastric cancer

Lee *et al*.

Supplementary Figures & Tables

Supplementary Note

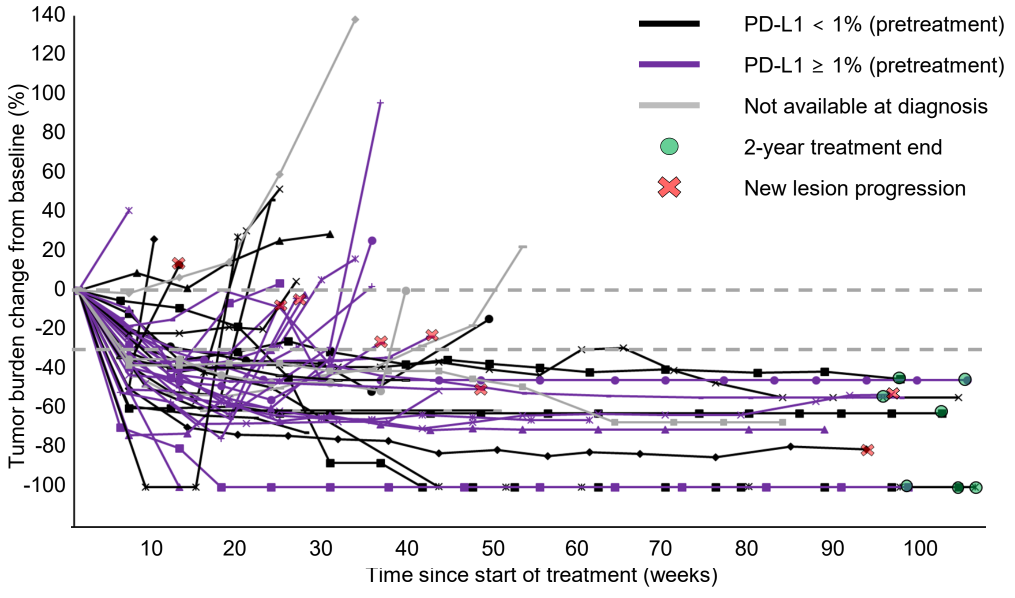

Supplementary Figure 1: **Changes in tumor burden over time.**
Spider plot of tumor burden changes from baseline in percentages (n=43).

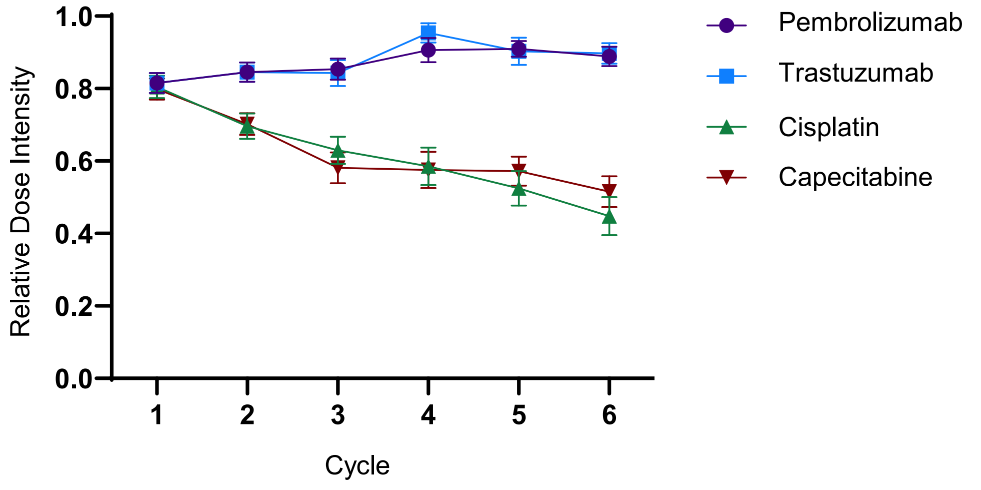

Supplementary Figure 2: **Relative dose intensity (RDI) per cycle, n=43**
RDI is defined as (administered dose per planned dose) divided by (actual duration per planned duration of therapy). Note that no dose reduction was performed for pembrolizumab and trastuzumab, but the RDI decreased due to the treatment delays.
A number for each point is provided in the source data.
Data are presented as mean values +/- SEM.

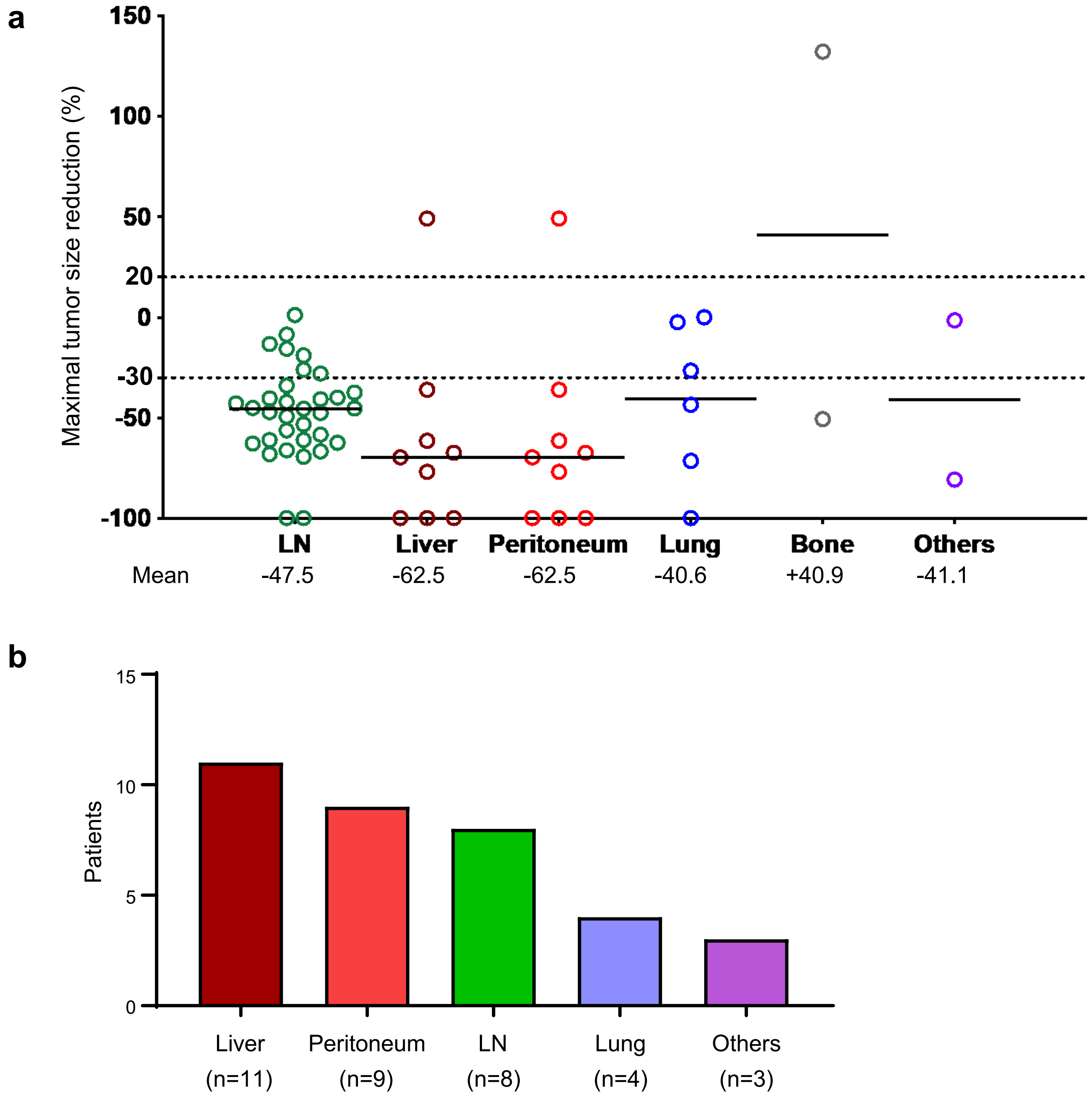

***Supplementary Figure 3*. Responses per metastatic organs**

a Maximal metastatic tumor size reduction per organ in the 41 evaluable patients. Others included adrenal gland (n=1) and esophageal (n=1) metastatic lesions.

b Bar graph showing metastatic organs of progression in the 32 evaluable patients. Others included adrenal gland (n=2) and brain (n=1) metastatic lesions.

*Only measurable lesions were analyzed.

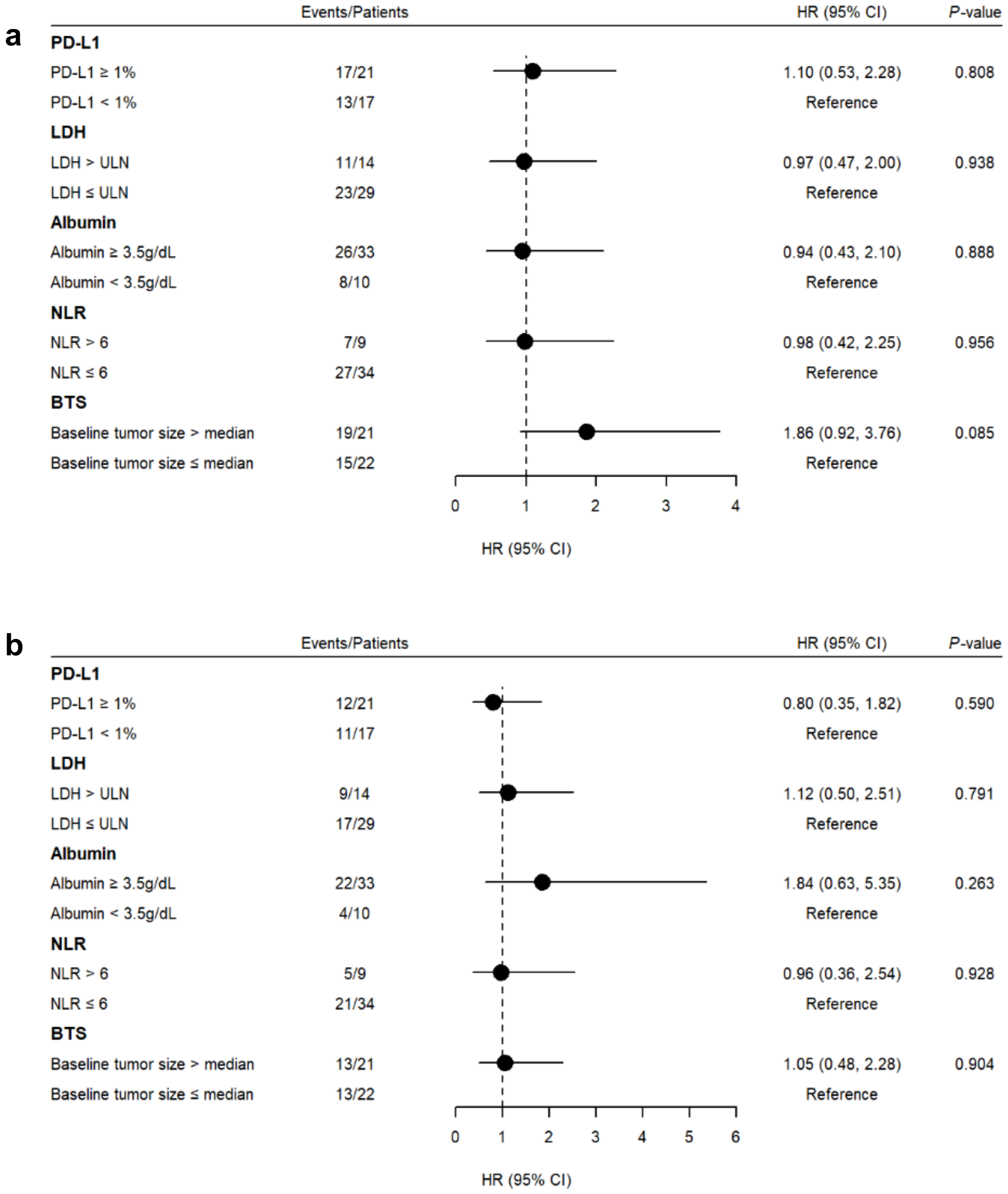

***Supplementary Figure 4*. Forest plot of hazard ratios for progression-free survival (a) and overall survival (b) according to patient characteristics at baseline**

Data are presented as the event rate, estimated hazard ratio values (black circle) with the corresponding 95% CI (error bars), two-sided P-values based on the Cox proportional hazard regression model.

LDH, lactate dehydrogenase level; NLR, neutrophil-to-lymophocyte ratio; BTS, baseline tumor size (median baseline tumor size=100.74mm); ULN, upper limit of normal (247 U/L for LDH).

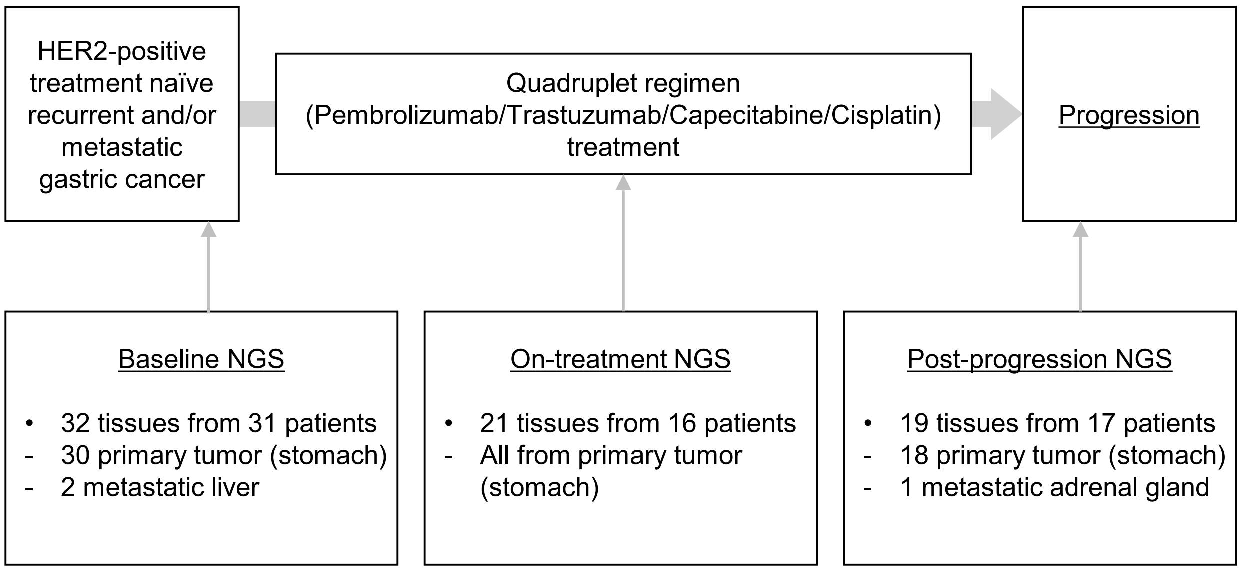

Supplementary Figure 5. **Schematic diagram showing sequential genomic analysis in available patient samples, n=38**

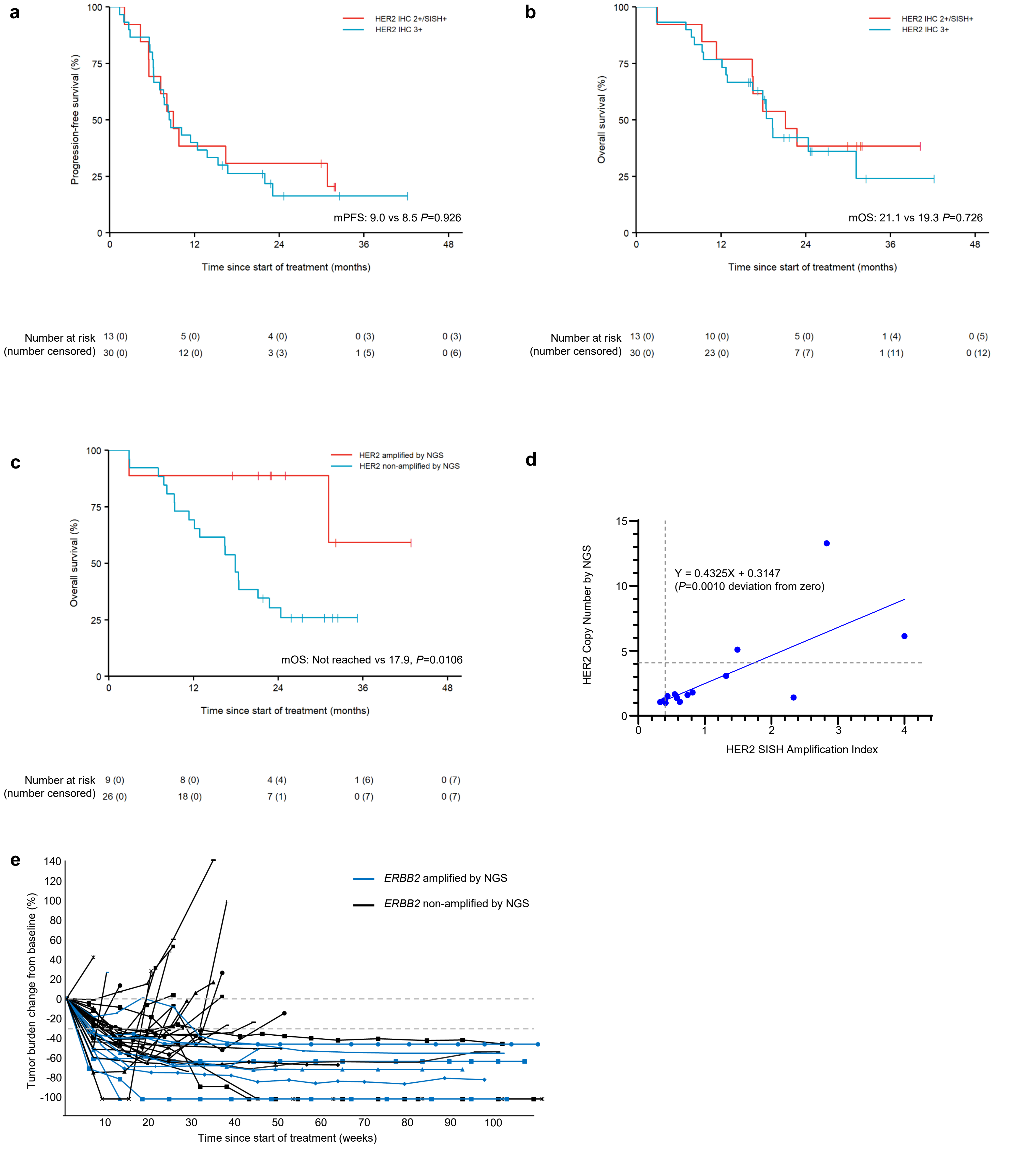

***Supplementary Figure 6*. Correlation between baseline HER2 status and survival**

a,b Kaplan Meier survival curves for progression-free survival (PFS) and overall survival (OS) in months stratified by baseline HER2 IHC status (n=43). **c** Kaplan Meier survival curves with OS in months according to baseline *ERBB2* amplification by NGS (n=35). In (**a**-**c**), two-sided P-values for survival associations were calculated using the log-rank tests. Hazard ratios and corresponding 95% CIs were estimated using Cox proportional hazard regression model. No adjustments for multiple comparisons were made. Crosses denote censored observation and number at risk is indicated below the plots. **d** Correlation between *HER2* copy number by NGS and HER2 SISH amplification index. Linear regression (blue line) analysis demonstrates significant correlation between HER2 copy number by NGS and HER2 SISH amplification index (n=17, two-sided Spearman correlation coefficient=0.7919, P-value=0.0002 and R-square=0.5246). Linear regression coefficients and significant P-value for the differences of the slope from 0 (derived from the t-statistic) were shown in the figure. **e** Percentage change from baseline with total tumor lesions of measurable target and non-target lesions over time, according to baseline *ERBB2* amplification by NGS (n=31). All the patients were HER2-positive by IHC or SISH.

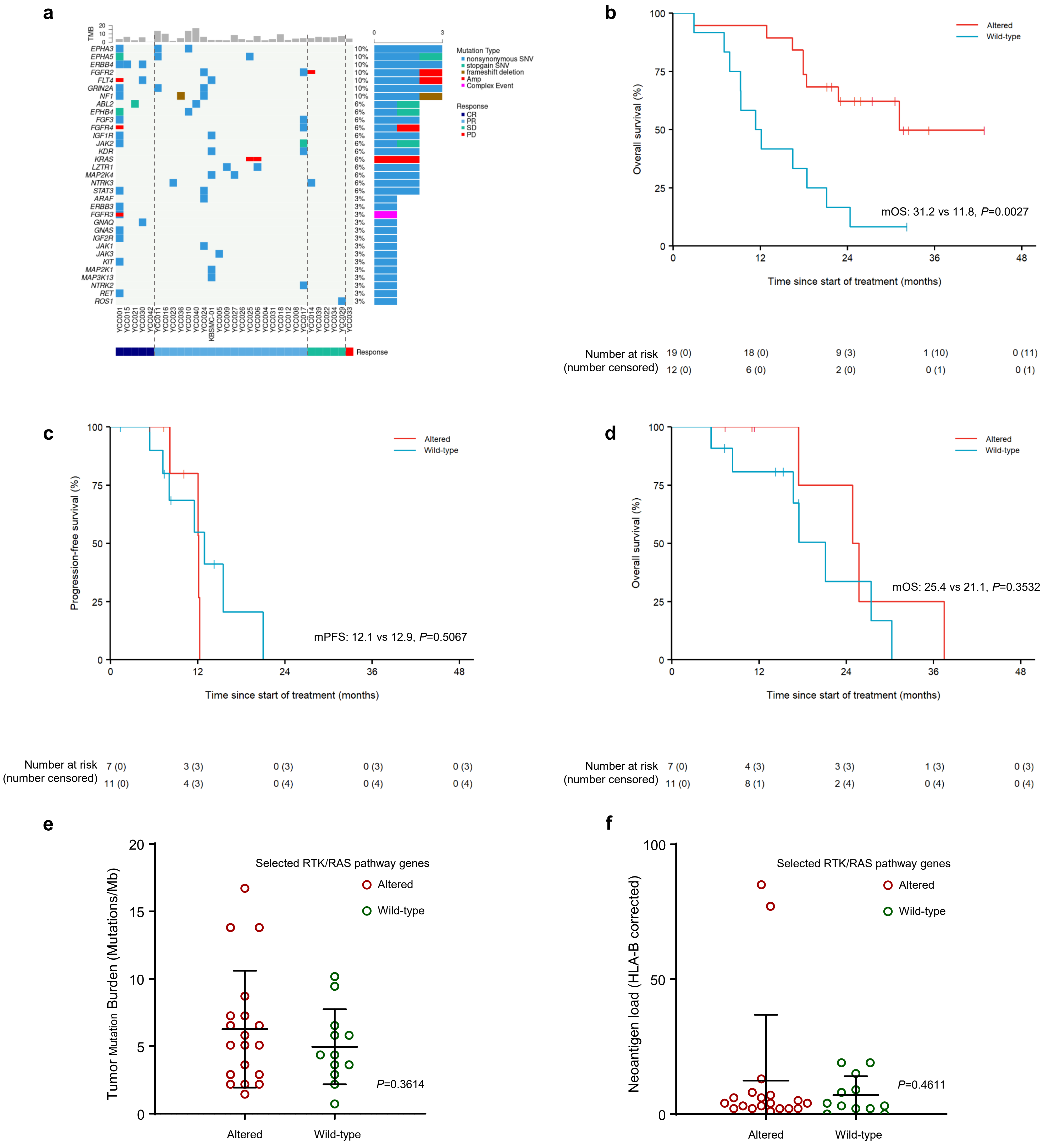

***Supplementary Figure 7*. Selected RTK/RAS pathway genes and patient survival from baseline tissue NGS**

a The detected molecular alterations on RTK/RAS pathway genes, except ERBB2 (n=31). **b** Kaplan Meier survival curves with overall survival (OS) of this study in months by alteration of selected RTK/RAS pathway genes (RTK/RAS pathway altered, n=19, and wild-type, n=12). **c,d** Kaplan Meier survival curves with progression-free survival (PFS, **c**) and overall survival (OS, **d**) in months by alteration of selected RTK/RAS pathway genes from historical retrospective cohort gastric cancer patients who were treated with Trastuzumab+Capeciatbine+Cisplatin as palliative 1st line treatment (RTK/RAS pathway altered, n=7, and wild-type, n=11). In (**b-d**), two-sided P-values for survival associations were calculated using the log-rank tests. Hazard ratios and corresponding 95% CIs were estimated using Cox proportional hazard regression model. No adjustments for multiple comparisons were made. Crosses denote censored observation and number at risk is indicated below the plots. **e** Association between tumor mutation burden from baseline tumor tissue NGS and group by RTK/RAS pathway gene alteration. **f** Association between neoantigen load (HLA-B corrected) from baseline tumor tissue NGS and group by RTK/RAS pathway gene alteration. In (**e**, **f**), P-values were calculated using a two-sided independent t-test.
Bars and error bars, mean±SD.

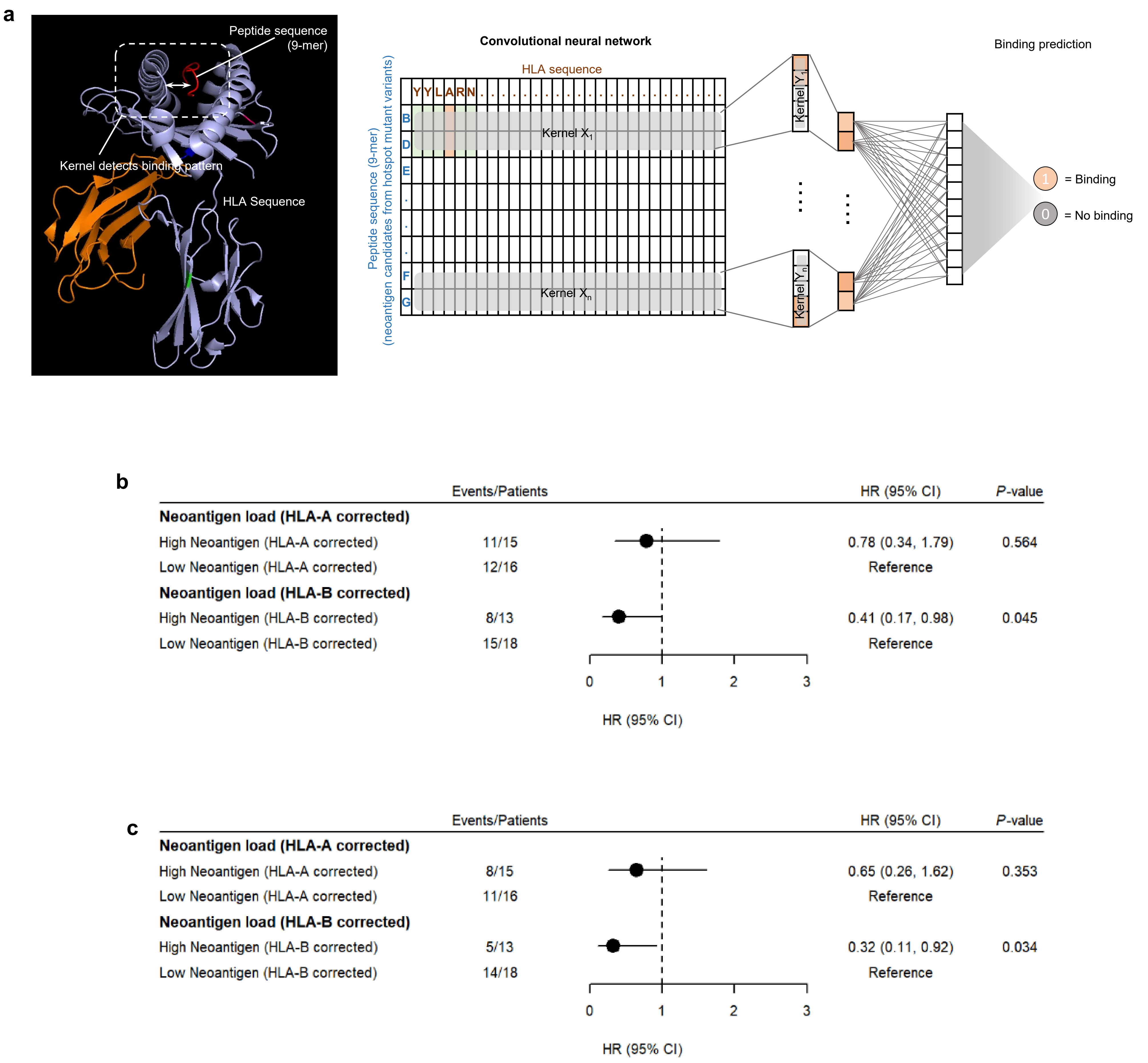

***Supplementary Figure 8*. Neoantigen load predicted by the CNN model and survival**
a Schematic 3D model for peptide-MHC class I binding (left). Schematic diagram showing HLA molecule and diagram showing estimation of neoantigen load by the convolutional neural network (CNN) according to individual patient's somatic mutations and HLA-A or HLA-B alleles (right). Modified from Kim *et al*., Nature Communications 2020. See methods for the details. Forest plot of hazard ratios for progression-free survival (**b**) and overall survival (**c**) according to neoantigen load predicted by neoantigen load predicted, stratified by pre-defined cutoff (> median indicates high neoantigen load and ≤ median indicates low neoantigen load). Data are presented as the event rate, estimated hazard ratio values (black circle) with the corresponding 95% CI (error bars), two-sided P-values based on the Cox proportional hazard regression model.
HLA-A corrected median neoantigen load, 13 peptides; HLA-B corrected median neoantigen load, 4 peptides.

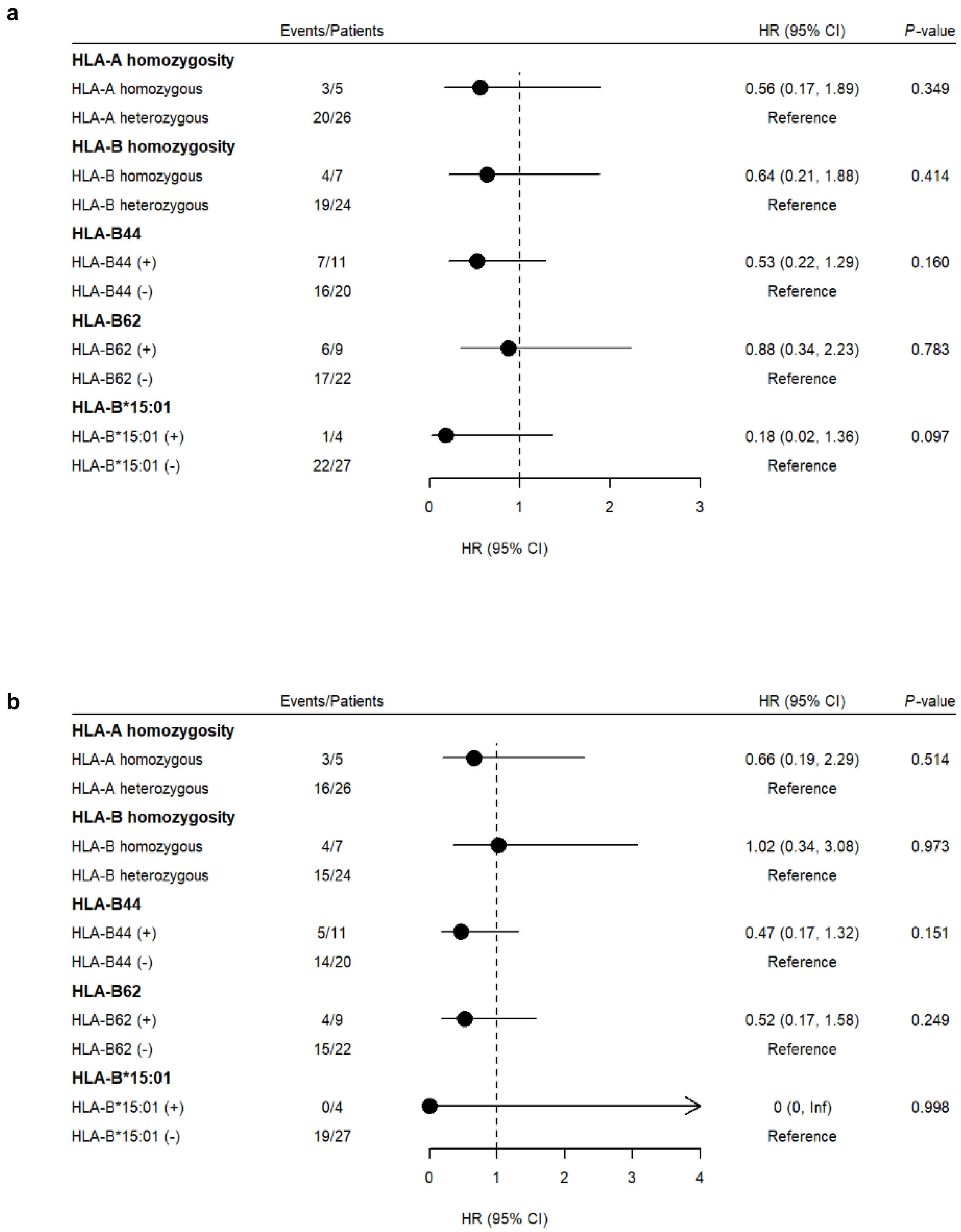

***Supplementary Figure 9*. Forest plot of hazard ratios for progression-free survival (a) and overall survival (b) according to patient HLA homozygosity, supertypes or allele.**
Data are presented as the event rate, estimated hazard ratio values (black circle) with the corresponding 95% CI (error bars), two-sided P-values based on the Cox proportional hazard regression model.

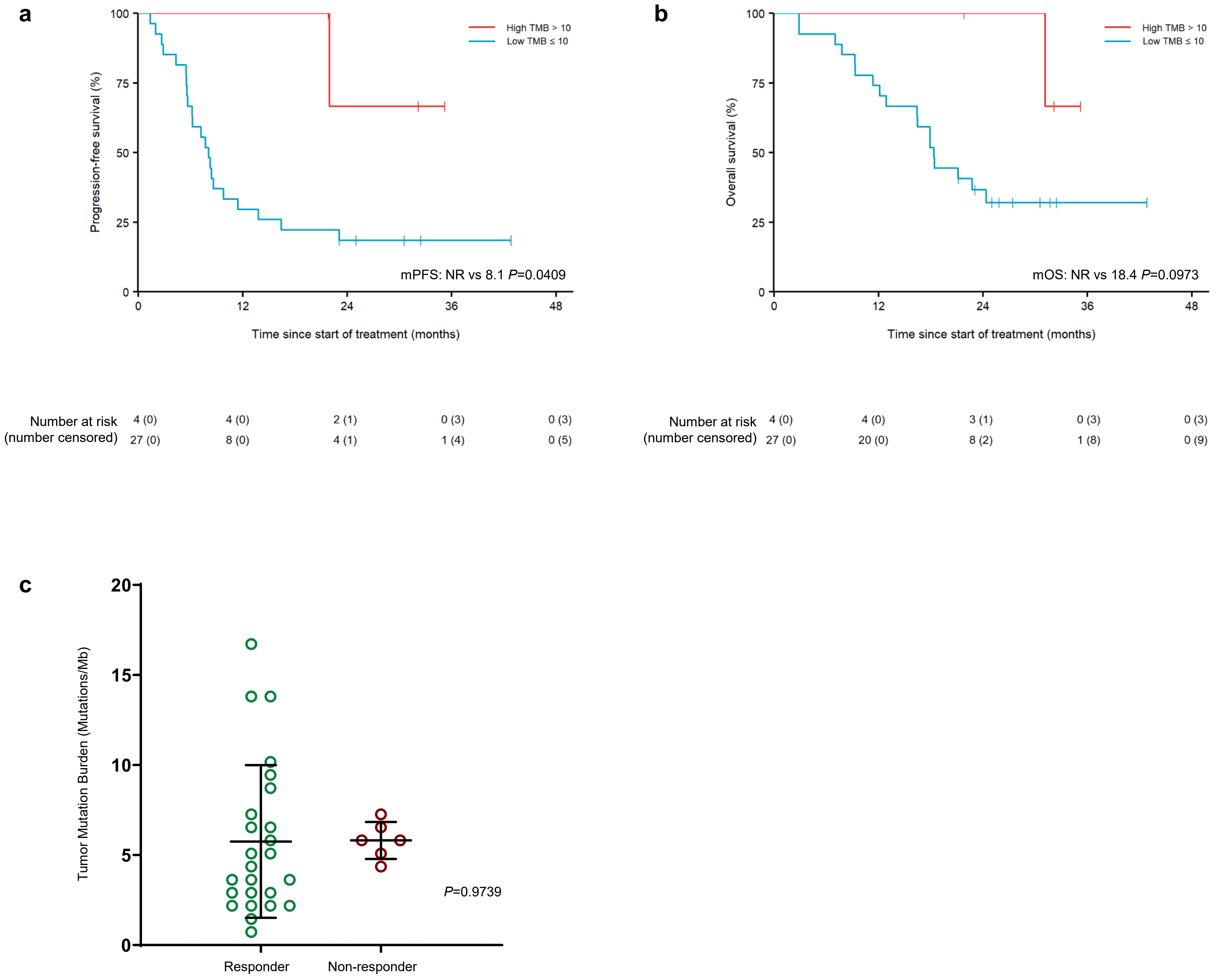

***Supplementary Figure 10*. Tumor mutation burden and survival from baseline tissue NGS, n=31**
a,b Kaplan Meier survival curves with progression-free survival (PFS, **a**) and overall survival (OS, **b**) in months by tumor mutation burden stratified by pre-defined cutoff (>10 mutations/Mb indicates high TMB, n=4, and ≤10 mutations/Mb indicates low TMB, n=27). In (**a**-**b**), two-sided P-values for survival associations were calculated using the log-rank tests. Hazard ratios and corresponding 95% CIs were estimated using Cox proportional hazard regression model. No adjustments for multiple comparisons were made. Crosses denote censored observation and number at risk is indicated below the plots. NR, not reached. **c** Association between tumor mutation burden from baseline tumor tissue NGS and objective response to the pembrolizumab, trastuzumab, and chemotherapy(responder, n=25, and non-responder, n=6). Bars and error bars, mean±SD. P-value were calculated using a two-sided independent t-test.

a

Gene	AA change	YCC031	YCC006	YCC008	KBSMC-01	YCC012	YCC001	YCC027	YCC010
PMS2	K706X	46							
ATM	R2443X		29						
ATM	R2993X			46					
PALB2	R1086X				1				
RAD51B	Q371X					2	3		
BAP1	Q392X			1					
FANCA	R853X				1				
FANCM	E454X							6	
PARP1	R340X						3		
PARP2	W74X						1		
PARP4	R1512X						2		
ARID1A	E1895X								64

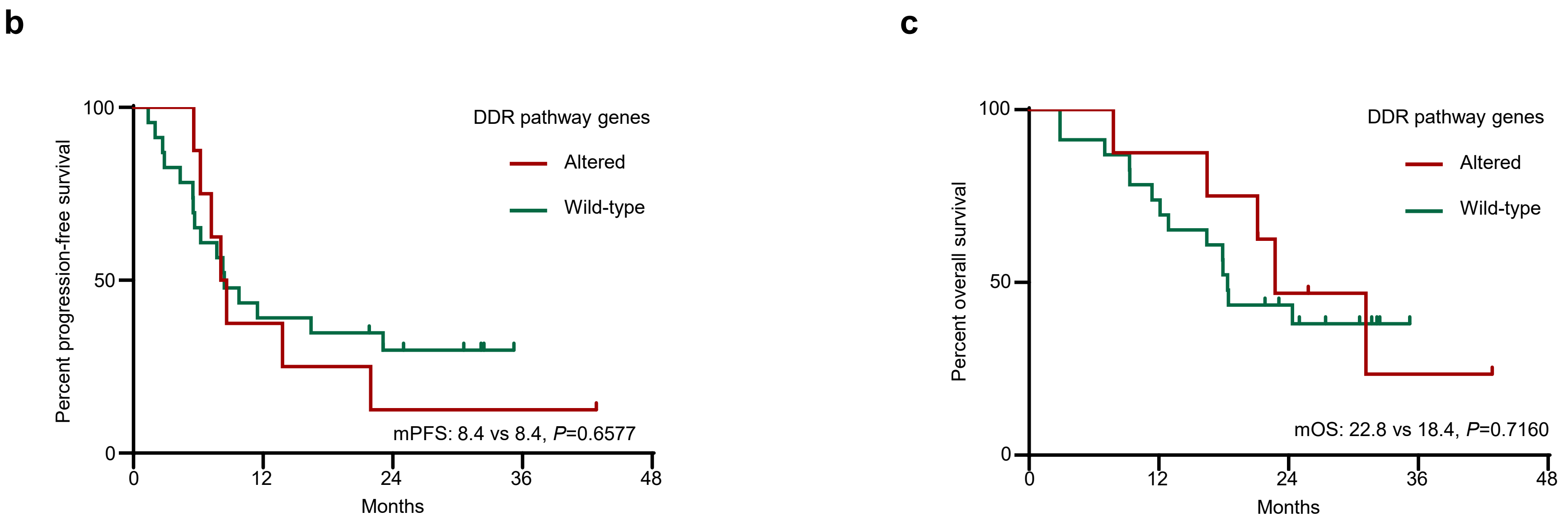

Supplementary Figure 11. **DNA damage response pathway genes and patient survival from baseline tissue NGS, n=31**

a Molecular alterations in DNA damage response (DDR) pathway genes among patients. Numbers indicate variant allele frequencies (VAF, %). **b,c** Kaplan-Meier survival curves with progression-free survival (PFS, **b**) and overall survival (OS, **c**) in months based on alterations of DDR pathway genes. In (**b-c**), two-sided P-values for survival associations were calculated using the log-rank tests. Crosses denote censored observation and number at risk is indicated below the plots.

Best response*	
Complete response	6 (14.0%)
Partial response	27 (62.8%)
Stable disease	9 (20.9%)
Progressive disease	1 (2.3%)
Objective response rate (ORR)	33 (76.7%)
Disease control rate	42 (97.7%)
ORR at 12 week	31 (72.1%)
ORR at 24 week	29 (67.4%)
Duration of response (DOR)	
Median time to response	1.7 months (range, 1.2-5.8)
Median DOR	10.8 months (95% CI 7.2-21.8)

*confirmed response per RECIST v1.1
Two patients who underwent conversion surgery achieved confirmed CR and PR each, before conducting conversion surgery.

***Supplementary Table 1*. Response details per RECIST v1.1**

	Median (Interqaurtile range)
Total treatment cycles	12 (8-24)
Pembrolizumab+Trastuzumab (Pem+T)	12 (8-24)
Capecitabine (X)	8 (6-12)
Cisplatin (P)	6 (4-7)
Quadruplet cycles (Pem+T+XP)	6 (4-7)
Chemotherapy-free maintenance	
Cisplatin-free cycles (Pem+T±X)	5 (1-20)
Capecitabine-free cycles (Pem+T±P)	1 (0-13)
XP-free cycles (Pem+T)	1 (0-13)
Maintenance cycles (beyond 6th cycle)*	6 (2-18)

*Number of cycles before 6th cycles was also included when XP were omitted earlier.

***Supplementary Table 2*. Treatment cycles**

ABL1	ABL2	**ACVR1**	ACVR1B	ADAM29	**ADGRA2**	**AKT1**	**AKT2**
AKT3	***ALK***	**ALOX12B**	**ALOX15B**	AMER1	**APC**	**APCDD1**	**APEX1**
APOB	APOBEC1	APOBEC3A	**APOBEC3B**	**AR**	ARAF	**ARFRP1**	ARID1A
ARID1B	**ARID2**	ASXL1	**ATM**	**ATP11B**	ATR	ATRX	AURKA
AURKB	**AXIN1**	AXL	B2M	B3GAT1	BACH1	**BAP1**	BARD1
**BCL2**	BCL2A1	**BCL2L1**	BCL2L2	BCL6	**BCL9**	BCOR	BCORL1
BCR	**BIRC2**	**BIRC3**	BLM	***BRAF***	***BRCA1***	**BRCA2**	BRD2
BRD3	**BRD4**	BRIP1	**BTG1**	BTK	BTLA	CARD11	CASP5
CASP8	CBFB	CBL	CCDC150	CCDC168	CCDC43	CCL2	CCL4
**CCND1**	CCND2	CCND3	**CCNE1**	CD27	**CD274**	CD276	CD28
CD3D	CD3E	CD3G	CD4	CD40	**CD44**	CD79A	CD79B
CD8A	CDC42	CDC73	**CDH1**	CDH2	CDH20	CDH5	CDK12
**CDK4**	**CDK6**	CDK8	CDKN1A	CDKN1B	**CDKN2A**	**CDKN2B**	CDKN2C
CDX2	CEBPA	**CHD1**	CHD2	CHD4	CHEK1	CHEK2	**CHUK**
CIC	**CRBN**	CREBBP	CRKL	CRLF2	CSF1R	CSF2	CSF2RA
CSF2RB	**CSNK2A1**	CTCF	CTLA4	CTNNA1	CTNNB1	CUL3	CUL4A
CUL4B	CXCL10	CXCL11	CXCL9	CXCR3	CYLD	CYP17A1	**DAXX**
**DCUN1D1**	**DDR2**	DICER1	**DIS3**	DNMT1	DNMT3A	DOCK2	DOT1L
EGFR	ELMO1	**EML4**	EMSY	EP300	EPHA3	EPHA5	EPHA6
EPHA7	EPHB1	EPHB4	EPHB6	***ERBB2***	ERBB3	ERBB4	ERCC1
ERCC2	ERG	ERRFI1	ESR1	**ETV1**	ETV4	ETV5	ETV6
EWSR1	EYA2	EZH2	**FAM46C**	FANCA	FANCC	FANCD2	FANCE
**FANCF**	FANCG	FANCI	FANCL	**FANCM**	FAS	FAT1	FAT3
**FBXW7**	FGF1	**FGF10**	FGF12	FGF14	FGF19	FGF2	FGF23
FGF3	FGF4	FGF6	FGF7	***FGFR1***	***FGFR2***	***FGFR3***	**FGFR4**
FH	FLCN	**FLT1**	**FLT3**	**FLT4**	FOXA1	FOXL2	FOXO3
FOXP1	FOXP3	FRS2	FUBP1	GABRA6	**GAS6**	GATA1	GATA2
**GATA3**	GATA4	GATA6	**GID4**	GLI1	GNA11	GNA13	GNAQ
GNAS	GRIN2A	GRM3	GSK3B	GUCY1A2	GZMA	GZMB	GZMH
H3F3A	HGF	**HIST1H3B**	HLA-A	HLA-B	HLA-C	HLA-DRA	HLA-E
HLA-F	HLA-G	**HNF1A**	HOXA3	**HRAS**	HSD3B1	HSP90AA1	IDH1
**IDH2**	IDO1	IDO2	IFITM1	IFITM3	IFNA1	IFNB1	IFNG
IGF1	**IGF1R**	IGF2	IGF2R	**IKBKE**	IKZF1	IL12A	IL12B
IL2	IL23A	**IL6**	**IL7R**	**INHBA**	INPP4B	INSR	IRF2
**IRF4**	IRS2	ITGAE	ITK	JAK1	**JAK2**	JAK3	JUN
KAT6A	KDM5A	KDM5C	KDM6A	KDR	KEAP1	KEL	**KIT**
KLF4	KLHL6	KMT2A	KMT2B	KMT2C	KNSTRN	**KRAS**	LAG3
**LMO1**	LRP1B	LRP6	LTK	LYN	LZTR1	MAGI2	MAGOH
**MAML1**	MAP2K1	MAP2K2	**MAP2K4**	MAP3K1	MAP3K13	MAPK1	MAX
**MCL1**	**MDM2**	**MDM4**	MED12	MEF2B	MEN1	***MET***	MITF
MLH1	MPL	**MRE11**	**MSH2**	MSH6	MTOR	MUTYH	MYB
**MYC**	**MYCL**	**MYCN**	MYD88	**MYO18A**	NCOA3	NCOR1	**NF1**
**NF2**	NFE2L2	NFKBIA	**NKX2-1**	**NKX2-8**	NKX3-1	**NOTCH1**	**NOTCH2**
**NOTCH3**	**NOTCH4**	NPM1	NRAS	NSD1	**NSD3**	*NTRK1*	*NTRK2*
NTRK3	NUP93	NUTM1	PAK3	PAK5	PALB2	PARP1	PARP2
**PARP3**	PARP4	PAX5	**PBRM1**	PDCD1	**PDCD1LG2**	***PDGFRA***	PDGFRB
PDK1	PGR	PHF6	PHLPP2	PIK3C2B	**PIK3C3**	**PIK3CA**	PIK3CB
PIK3CG	**PIK3R1**	PIK3R2	PKHD1	PLCG1	PLCG2	PMS2	**PNP**
PNRC1	POLD1	POLE	***PPARG***	PPP2R1A	**PRDM1**	PREX2	PRF1
PRKAR1A	PRKCI	PRKDC	PARK2	PRPF40B	PRSS8	**PTCH1**	PTCH2
**PTEN**	**PTK2**	PTPN11	PTPRC	**PTPRD**	QKI	**RAB35**	RAC1
RAC2	RAD17	RAD50	RAD51	RAD51B	RAD51C	RAD51D	RAD52
RAD54L	RAF1	RANBP2	RARA	**RB1**	RBM10	REL	*RET*
RHEB	RHOA	RHOB	**RICTOR**	RNF43	ROBO1	ROBO2	*ROS1*
**RPA1**	**RPS6KB1**	RPTOR	**RUNX1**	**RUNX1T1**	RUNX3	SDHA	SDHB
SDHC	SDHD	**SEMA3A**	SEMA3E	SET	**SETBP1**	SETD2	SF3A1
SF3B1	**SH2B3**	**SKP2**	SLIT2	**SMAD2**	SMAD3	**SMAD4**	SMARCA1
SMARCA4	**SMARCB1**	SMARCD1	SMO	SNCAIP	**SOCS1**	SOX10	**SOX2**
SOX9	SPEN	SPOP	SPTA1	SRC	SRSF1	SRSF2	SRSF7
STAG2	STAT3	STAT4	**STK11**	SUFU	SYK	TACC3	TAF1
TBX22	TBX3	TERC	**TERT**	**TET2**	TGFBR2	**TIAF1**	TIGIT
TIPARP	TMPRSS2	TNF	TNFAIP3	TNFRSF14	TNFRSF18	TNFRSF4	TNFSF13B
TNKS	**TNKS2**	TOP1	TOP2A	**TP53**	TRAF7	TRRAP	**TSC1**
**TSC2**	TSHR	U2AF1	U2AF2	USP9X	VEGFA	**VHL**	VSIR
VTCN1	WISP3	WNT1	**WT1**	**WWP1**	XBP1	XPO1	XRCC3
ZBTB2	**ZNF217**	**ZNF703**	ZRSR2				

Bold : CNV (143 genes), ***italic*** **: translocation (18 genes)**

***Supplementary Table 3*. In-house NGS sequencing panel**

Patient ID	Pretreatment NGS tissue	Pretreatment NGS HER2 amplification	HER2 copy number	HER-2 IHC	HER2 SISH AI[#]	Best Response
YCC030	Stomach	Amplified	8.78	3+	Not assessed	CR
YCC001	Stomach	Amplified	6.13	3+	20	CR
YCC021	Stomach	Non-amplified	1.66	2+	2.74	CR
YCC015	Stomach	Non-amplified	1.59	2+	3.7	CR
YCC042	Stomach	Non-amplified	1.18	2+	2.01	CR
YCC003	Not done			2+	2.9	CR
YCC037	Not done	Amplified*		3+	Not assessed	CR
YCC016	Stomach	Amplified	28.21	2+	>2.0	PR
YCC040	Stomach	Amplified	20.10	3+	Not assessed	PR
KBSMC-01	Stomach	Amplified	13.28	3+	14.16	PR
YCC010	Stomach	Amplified	13.07	3+	Not assessed	PR
YCC036	Stomach	Amplified	9.95	3+	Not assessed	PR
YCC012	Stomach	Non-amplified	3.07	3+	6.6	PR
YCC023	Stomach	Non-amplified	3.03	3+	Not assessed	PR
YCC004	Stomach	Non-amplified	2.94	3+	Not assessed	PR
YCC011	Stomach	Non-amplified	2.88	3+	Not assessed	PR
YCC005	Liver	Non-amplified	2.13	3+	Not assessed	PR
YCC026	Stomach	Non-amplified	1.99	3+	Not assessed	PR
YCC018	Stomach	Non-amplified	1.92	3+	Not assessed	PR
YCC024	Stomach	Non-amplified	1.79	2+	4.07	PR
YCC006	Stomach	Non-amplified	1.52	2+	2.19	PR
YCC027	Stomach	Non-amplified	1.36	3+	2.9	PR
YCC025	Stomach	Non-amplified	1.18	3+	1.921	PR
YCC008	Stomach	Non-amplified	1.10	3+	Not assessed	PR
YCC031	Liver	Non-amplified	1.07	2+	3.12	PR
YCC017	Stomach	Non-amplified	1.00	2+	2.05	PR
YCC009	Stomach	Non-amplified	0.98	2+	2.05	PR
CNUH-01	Not done			2+	2.38	PR
CNUH-02	Not done			3+	Not assessed	PR
YCC019	Not done			3+	Not assessed	PR
YCC028	Not done			3+	Not assessed	PR
YCC035	Not done			3+	Not assessed	PR
YCC041	Not done	Amplified*		3+	Not assessed	PR
YCC022	Stomach	Amplified	5.10	3+	7.452	SD
YCC034	Stomach	Non-amplified	1.83	3+	Not assessed	SD
YCC039	Stomach	Non-amplified	1.41	2+	2.89	SD
YCC014	Stomach	Non-amplified	1.41	3+	11.67	SD
YCC029	Stomach	Non-amplified	1.05	3+	1.643	SD
YCC002	Not done			3+	Not assessed	SD
YCC020	Not done	Non-amplified*		2+	2.08	SD
YCC032	Not done			3+	Not assessed	SD
YCC038	Not done	Non-amplified*		3+	Not assessed	SD
YCC033	Stomach	Non-amplified	2.52	3+	Not assessed	PD

*FoundationOne® panel sequencing results

[#]Amplification index from silver *in situ* hybridization

***Supplementary Table 4*. HER2 status of each patient measured using different technologies and clinical responses**

ID	Hugo Gene Symbol	Amino Acid Change	Seqeunce Change	ClinVar¶	ACMG†	COSMIC ID	Pathogenic Predictiion※
KBSMC-01	MAP2K1	D67N	G199A	Pathogenic	Pathogenic	COSV61068427	Pathogenic
KBSMC-01	MAP3K13	E452K	G1354A		VUS	COSV53988542	Pathogenic
KBSMC-01	MAP2K4	G249S	G745A		VUS	COSV99065793	Pathogenic
KBSMC-01	FLT4	R592C	C1774T		VUS	COSV56125517	Pathogenic
KBSMC-01	IGF1R	R794W	C2380T		VUS	COSV51308319	Pathogenic
KBSMC-01	KDR	T446M	C1337T		VUS	COSV55773759	Pathogenic
YCC001	FGFR3	R621H	G1862A	Pathogenic	Likely Pathogenic	COSV53404768	Pathogenic
YCC001	GNAS	R258W	C772T	Pathogenic	VUS	COSV105009920	Pathogenic
YCC001	EPHB4	R763X	C2287T		Likely Pathogenic	COSV100639474	Pathogenic
YCC001	FGFR3	D513N	G1537A	Uncertain_significance	Likely Pathogenic	COSV53408477	Pathogenic
YCC001	EPHA3	R750Q	G2249A		VUS	COSV60692867	Pathogenic
YCC001	ERBB4	G319E	G956A		VUS	COSV53526682	Pathogenic
YCC001	FGF3	R132W	C394T		VUS	COSV100490411	Pathogenic
YCC001	STAT3	T138M	C413T		VUS	COSV52892130	Pathogenic
YCC001	ERBB3	R679Q	G2036A		VUS	COSV57252242	Pathogenic
YCC001	IGF1R	R719H	G2156A		VUS	COSV51295636	Pathogenic
YCC001	IGF2R	V813M	G2437A		VUS	COSV63630409	Pathogenic
YCC001	KIT	A895T	G2683A	Uncertain_significance	VUS	COSV55394257	Pathogenic
YCC001	NF1	A2797T	G8389A	Uncertain_significance	VUS	COSV62226413	Pathogenic
YCC001	GRIN2A	R19C	C55T	Uncertain_significance	VUS	COSV100408696	Pathogenic
YCC001	EPHA3	T933M	C2798T		VUS	COSV60695123	Pathogenic
YCC001	EPHA5	R238X	C712T		VUS	COSV56643225	Pathogenic
YCC001	IGF1R	R437H	G1310A	Benign	VUS	COSV51278179	N/A
YCC001	JAK2	G294S	G880A		VUS	COSV104431578	Pathogenic
YCC001	RET	V53I	G157A	Uncertain_significance	VUS	COSV99053636	N/A
YCC005	JAK3	R899W	C2695T		VUS	COSV71686337	Pathogenic
YCC006	LZTR1	R97W	C289T		VUS	COSV53146118	Pathogenic
YCC009	LZTR1	A744V	C2231T		VUS	COSV99302557	Pathogenic
YCC010	EPHA3	R160H	G479A		VUS	COSV60701234	Pathogenic
YCC010	EPHB4	T526P	A1576C		VUS	COSV100639510	Pathogenic
YCC011	EPHA3	N79I	A236T		VUS	COSV60692365	Pathogenic
YCC011	GRIN2A	R49C	C145T		VUS	COSV58021605	Pathogenic
YCC011	EPHA5	R417W	C1249T		VUS	COSV56638771	Pathogenic
YCC014	NTRK3	V97M	G289A		VUS	COSV58113091	Pathogenic
YCC015	ERBB4	K1223T	A3668C		VUS	COSV61475135	Pathogenic
YCC017	FGF3	M99I	G297C		VUS	COSV61927061	Pathogenic
YCC017	KDR	R1022Q	G3065A		VUS	COSV55760145	Pathogenic
YCC017	FGFR2	R421H	G1262A		VUS	COSV60661464	Pathogenic
YCC017	FGFR4	R78H	G233A		VUS	COSV52805339	Pathogenic
YCC017	JAK2	R426X	C1276T		VUS	COSV67593461	Pathogenic
YCC017	NTRK2	P65H	C194A		VUS	COSV52858481	Neutral
YCC021	ABL2	R503X	C1507T		VUS	COSV61015911	Pathogenic
YCC023	NTRK3	L115P	T344C		VUS	COSV58125113	Pathogenic
YCC024	STAT3	A46V	C137T		VUS	COSV52885480	Pathogenic
YCC024	FGFR2	R210Q	G629A		VUS	COSV60640963	Pathogenic
YCC024	JAK1	R454W	C1360T		VUS	COSV61097942	Pathogenic
YCC024	GRIN2A	S1390L	C4169T		VUS	COSV58030987	Pathogenic
YCC024	ARAF	V21I	G61A		VUS	COSV51695045	Pathogenic
YCC024	NF1	V288M	G862A	Uncertain_significance	VUS	COSV62216442	Pathogenic
YCC025	EPHA5	K978M	A2933T		VUS	COSV56639128	Pathogenic
YCC027	MAP2K4	E141K	G421A		VUS	COSV62261222	Pathogenic
YCC029	ROS1	I704T	T2111C		VUS	COSV100877912	Pathogenic
YCC030	ERBB4	N278S	A833G		VUS	COSV53511860	Pathogenic
YCC030	GNAQ	A231V	C692T		VUS	COSV54115267	Pathogenic
YCC030	FLT4	R189W	C565T		VUS	COSV56101712	Pathogenic
YCC036	NF1	G96fs	286delG	Pathogenic	Pathogenic	N/A	N/A
YCC040	ABL2	A88T	G262A		VUS	COSV61012148	Pathogenic

Clinical significance according to ClinVar¶ or American College of Medical Genetics (ACMG) criteria using a tool for assessment and prioritisation in exome studies (TAPES)†.
Pathogenic predictions※ were from COSMIC databse which were predicted by Functional Analysis Through Hidden Markov Models - Multiple Kernel Learning (FATHMM-MKL) algorithm.

***Supplementary Table 5*. RTK/RAS pathway gene alterations and their clinical significance and pathogenic prediction.**

Patient ID	Pretreatment NGS tissue	Pretreatment NGS HER2 amplification	Pretreatment NGS RTK/RAS pathway	HER-2 IHC	HER2 SISH AI	PFS	OS
Pt001	Esophagus	Non-amplified	Altered	3+	Not done	8.2	37.5
Pt002	Stomach	Non-amplified	Wild-type	2+	4.42	15.5	30.3
Pt003	Stomach	Non-amplified	Wild-type	3+	Not done	21.0	27.4
Pt004	Bronchus	Non-amplified	Altered	3+	Not done	12.1	25.8
Pt005	Lymph Node	Amplified	Altered	3+	Not done	12.1	24.9
Pt006	Stomach	Non-amplified	Wild-type	2+	2.5	11.5	21.1
Pt007	Stomach	Non-amplified	Wild-type	3+	Not done	8.1	17.5
Pt008	Stomach	Amplified	Wild-type	2+	5.29	8.3	17.5
Pt009	Stomach	Amplified	Altered	3+	Not done	12.3	17.4
Pt010	Stomach	Amplified	Wild-type	3+	Not done	12.9	16.7
Pt011	Stomach	Amplified	Wild-type	3+	Not done	7.4	15.3
Pt012	Stomach	Amplified	Wild-type	3+	Not done	14.3	14.3
Pt013	Stomach	Amplified	Altered	3+	Not done	5.4	11.4
Pt014	Stomach	Amplified	Altered	3+	Not done	10.1	11.0
Pt015	Stomach	Non-amplified	Wild-type	3+	Not done	7.2	8.4
Pt016	Stomach	Non-amplified	Altered	3+	Not done	7.3	7.3
Pt017	Stomach	Non-amplified	Wild-type	2+	2.14	1.3	7.3
Pt018	Stomach	Non-amplified	Wild-type	3+	Not done	5.4	5.4

***Supplementary Table 6*. HER2 status, RTK/RAS pathway changes and survival in each patient from historical HER2-positive AGC cohort with different technologies.**

	PFS			OS		
	HR	95% CI	*P*	HR	95% CI	*P*
HLA-B corrected neoAg load	0.75	0.27-2.11	0.59	0.75	0.21-2.67	0.651
RTK/RAS pathway alteration[a]	0.24	0.08-0.71	**0.01**	0.3	0.10-0.93	**0.037**
ERBB2 NGS amplification	0.2	0.05-0.72	**0.014**	0.27	0.06-1.21	0.086

[a]HER2 alterations excluded.

Supplementary Table 7. **Multivariable analysis of HLA-B corrected neo-antigen load, RTK/RAS pathway alterations, and *ERBB2* amplification**
The prognostic impact of HLA-B corrected neo-antigen load, RTK/RAS pathway alterations, and ERBB2 amplification were evaluated using a multivariable Cox proportional hazards model. Hazard ratios derived from multivariable models including all prognostic factors, and P-values calculated from a Wald-test.

a

ID	Gene	Amino Acid Change	Baseline	On-Treatment	Post-progression
YCC018	ADAM29	S768R	0.194502	NA	0.000017
YCC018	ADAM29	T746M	0.194502	NA	0.000017
YCC009	APOB	A43V	0.609385	0	NA
YCC016	ARID1A	D2178fs	0.484482	0	NA
YCC018	ARID1B	H89P	0.194502	NA	0.000017
YCC016	ATM	R2832C	0.484482	0	NA
YCC039	ATM	R1086H	0.048241	NA	0.002423
YCC024	AXIN1	R647C	0.31184	NA	0.000038
YCC016	BAP1	R60Q	0.484482	0	NA
YCC024	BCL2L2	V178M	0.31184	NA	0.000038
YCC023	BIRC3	V102I	0.335063	0.000009	NA
YCC009	CD28	T202P	0.609385	0	NA
YCC023	CD8A	R106H	0.335063	0.000009	NA
YCC024	CHD1	R803X	0.31184	NA	0.000038
YCC023	CSF1R	R676X	0.335063	0.000009	NA
YCC023	DICER1	R745X	0.335063	0.000009	NA
YCC008	DNMT3A	V296G	0.65843	0.000009	0.000015
YCC023	DOCK2	G365D	0.335063	0.000009	NA
YCC027	ELMO1	A5T	0.353494	NA	0.000005
YCC015	ERBB4	K1223T	0.042867	0.000048	NA
YCC024	FGFR2	R210Q	0.31184	NA	0.000038
YCC024	GRIN2A	S1390L	0.31184	NA	0.000038
YCC024	GRM3	R237C	0.31184	NA	0.000038
YCC023	GUCY1A2	M410I	0.335063	0.000009	NA
YCC018	HIST1H3B	E74Q	0.194502	NA	0.000017
YCC024	IL7R	R140Q	0.31184	NA	0.000038
YCC024	JAK1	R454W	0.31184	NA	0.000038
YCC016	KMT2B	A1649T	0.484482	0	NA
YCC024	KMT2C	R444W	0.31184	NA	0.000038
YCC009	LZTR1	A744V	0.609385	0	NA
YCC027	MAP2K4	E141K	0.353494	NA	0.000005
YCC023	MITF	R223H	0.335063	0.000009	NA
YCC008	MYC	Q48H	0.65843	0.157011	0.078394
YCC015	MYC	Q48H	0.042867	0.000048	NA
YCC033	MYC	Q48H	0.108978	NA	0.000024
YCC039	MYC	Q48H	0.048241	NA	0.002423
YCC027	NCOR1	Q1993X	0.353494	NA	0.000005
YCC023	NCOR1	E1109X	0.335063	0.000009	NA
YCC024	NF1	V288M	0.31184	NA	0.000038
YCC034	NOTCH1	A2037T	0.027326	0.001765	0.001616
YCC008	PHLPP2	R1177G	0.65843	0.000009	0.000015
YCC023	PIK3CA	E39K	0.335063	0.000009	NA
YCC023	PIK3R2	R620H	0.335063	0.000009	NA
YCC034	PKHD1	R2891C	0.027326	0.001765	0.001616
YCC009	PTCH2	V471I	0.609385	0	NA
YCC033	RPS6KB1	R335C	0.108978	NA	0.000024
YCC008	SDHA	G84E	0.65843	0.157011	0.078394
YCC016	SDHA	V446A	0.206421	0	NA
YCC023	SETBP1	R1146W	0.335063	0.000009	NA
YCC023	SMO	R772H	0.335063	0.000009	NA
YCC024	STAT3	A46V	0.31184	NA	0.000038
YCC016	TP53	R342X	0.484482	0	NA
YCC023	TSC2	V1703M	0.335063	0.000009	NA

b

ID	Gene	Amino Acid Change	Baseline	On-Treatment	Post-progression
YCC009	ADAM29	S768R	0	0.642329	NA
YCC016	ADAM29	T746M	0.003856	0.873013	NA
YCC009	AKT1	T443M	0	0.642329	NA
YCC024	APOB	A43V	0	NA	0.340143
YCC023	APOB	Q2352H	0.000006	0.07308	NA
YCC023	ATR	S89R	0.000006	0.07308	NA
YCC015	BRCA2	G2837X	0.002376	0.056988	NA
YCC034	CARD11	R75W	0	0	0.019705
YCC027	CASP8	Q166X	0.000021	NA	0.545687
YCC018	CDH2	A610S	0.000044	NA	0.189706
YCC023	CDK12	R983Q	0.000006	0.07308	NA
YCC033	CRBN	T403M	0.006947	NA	0.131892
YCC026	CTLA4	D123N	0.000577	0.001269	0.05736
YCC008	EP300	T1857P	0.000016	0.000016	0.08014
YCC039	EPHA5	K505N	0.002227	NA	0.038415
YCC024	EPHB1	A36T	0	NA	0.340143
YCC016	ERBB2	E280Q	0.003856	0.873013	NA
YCC026	ERBB2	D769H	0.000577	0.001269	0.05736
YCC016	ETV4	R387W	0.003856	0.873013	NA
YCC023	FAT3	S3761F	0.000048	0.079218	NA
YCC016	FBXW7	D560H	0.003856	0.873013	NA
YCC034	FGF12	G20V	0	0	0.019705
YCC026	FH	L417X	0.000577	0.001269	0.05736
YCC009	FUBP1	A43E	0	0.642329	NA
YCC026	GABRA6	F27V	0.000577	0.001269	0.05736
YCC009	GATA2	T176P	0	0.642329	NA
YCC034	GATA6	T537A	0	0	0.019705
YCC033	HLA-B	E299G	0.006947	NA	0.131892
YCC024	HNF1A	A161T	0	NA	0.340143
YCC023	IKZF1	D290N	0.000006	0.07308	NA
YCC023	INSR	R566G	0.000006	0.07308	NA
YCC026	JUN	S73L	0.000577	0.001269	0.05736
YCC009	KMT2B	A1510T	0	0.642329	NA
YCC026	MAGI2	L114V	0.000577	0.001269	0.05736
YCC016	MYC	Q48H	0.003856	0.873013	NA
YCC023	NOTCH2	E1674D	0.000006	0.07308	NA
YCC009	PIK3CA	D1029N	0	0.642329	NA
YCC023	PKHD1	F3587V	0.000048	0.079218	NA
YCC009	PREX2	S1140L	0	0.642329	NA
YCC023	PTK2	K163T	0.000006	0.07308	NA
YCC016	RANBP2	W1669C	0.003856	0.873013	NA
YCC026	ROBO1	R1170G	0.000577	0.001269	0.05736
YCC026	SEMA3E	E726D	0.000577	0.001269	0.05736
YCC023	SETBP1	R498W	0.000006	0.07308	NA
YCC008	SPTA1	K1732T	0.000016	0.000016	0.08014
YCC016	TSC2	R1529W	0.003856	0.873013	NA
YCC016	WT1	H465N	0.003856	0.873013	NA

***Supplementary Table 8*. Genes with hotspot mutation from sensitive (a) or resistant (b) sub-clones and their sub-clonal fraction.**

a

ID	Description	GeneRatio	BgRatio	pvalue	p.adjust	qvalue	geneID
R-HSA-5663202	**Diseases of signal transduction**	12/40	377/10654	7.39E-09	3.09E-06	2.07E-06	MYC/TSC2/STAT3/PIK3R2/PIK3CA/NOTCH1/NF1/NCOR1/FGFR2/ERBB4/CD28/AXIN1
R-HSA-2219528	**PI3K/AKT Signaling in Cancer**	6/40	101/10654	1.85E-06	0.000387	0.000259	TSC2/PIK3R2/PIK3CA/FGFR2/ERBB4/CD28
R-HSA-199418	Negative regulation of the PI3K/AKT network	6/40	110/10654	3.05E-06	0.000426	0.000284	PIK3R2/PIK3CA/PHLPP2/FGFR2/ERBB4/CD28
R-HSA-1257604	PIP3 activates AKT signaling	8/40	264/10654	4.94E-06	0.000518	0.000346	TSC2/TP53/PIK3R2/PIK3CA/PHLPP2/FGFR2/ERBB4/CD28
R-HSA-2219530	Constitutive Signaling by Aberrant PI3K in Cancer	5/40	75/10654	8.2E-06	0.000643	0.000429	PIK3R2/PIK3CA/FGFR2/ERBB4/CD28
R-HSA-1266695	**Interleukin-7 signaling**	4/40	36/10654	9.2E-06	0.000643	0.000429	STAT3/PIK3R2/JAK1/IL7R
R-HSA-9006925	Intracellular signaling by second messengers	8/40	305/10654	1.42E-05	0.000852	0.00057	TSC2/TP53/PIK3R2/PIK3CA/PHLPP2/FGFR2/ERBB4/CD28
R-HSA-451927	**Interleukin-2 family signaling**	4/40	44/10654	2.08E-05	0.001087	0.000726	STAT3/PIK3R2/PIK3CA/JAK1
R-HSA-6811558	PI5P, PP2A and IER3 Regulate PI3K/AKT Signaling	5/40	103/10654	3.85E-05	0.001699	0.001136	PIK3R2/PIK3CA/FGFR2/ERBB4/CD28
R-HSA-449147	Signaling by Interleukins	9/40	461/10654	4.06E-05	0.001699	0.001136	MYC/TP53/STAT3/PIK3R2/PIK3CA/MAP2K4/JAK1/IL7R/CSF1R
R-HSA-389357	CD28 dependent PI3K/Akt signaling	3/40	22/10654	7.19E-05	0.002737	0.001829	PIK3R2/PIK3CA/CD28
R-HSA-9006931	Signaling by Nuclear Receptors	7/40	299/10654	0.000108	0.003754	0.002509	MYC/PIK3R2/PIK3CA/NCOR1/ERBB4/CHD1/AXIN1
R-HSA-164952	The role of Nef in HIV-1 replication and disease pathogenesis	3/40	27/10654	0.000135	0.004032	0.002695	ELMO1/DOCK2/CD28
R-HSA-912526	Interleukin receptor SHC signaling	3/40	27/10654	0.000135	0.004032	0.002695	PIK3R2/PIK3CA/JAK1
R-HSA-8939211	ESR-mediated signaling	6/40	223/10654	0.000166	0.004385	0.00293	MYC/PIK3R2/PIK3CA/ERBB4/CHD1/AXIN1
R-HSA-186763	Downstream signal transduction	3/40	29/10654	0.000167	0.004385	0.00293	STAT3/PIK3R2/PIK3CA
R-HSA-389356	CD28 co-stimulation	3/40	33/10654	0.000247	0.006098	0.004075	PIK3R2/PIK3CA/CD28
R-HSA-9607240	FLT3 Signaling	6/40	267/10654	0.000439	0.010224	0.006832	PIK3R2/PIK3CA/NF1/JAK1/FGFR2/ERBB4
R-HSA-3247509	Chromatin modifying enzymes	6/40	274/10654	0.000504	0.010536	0.007041	DNMT3A/ARID1B/NCOR1/KMT2C/KMT2B/ARID1A
R-HSA-4839726	Chromatin organization	6/40	274/10654	0.000504	0.010536	0.007041	DNMT3A/ARID1B/NCOR1/KMT2C/KMT2B/ARID1A

b

ID	Description	GeneRatio	BgRatio	pvalue	p.adjust	qvalue	geneID
R-HSA-157118	**Signaling by NOTCH**	7/37	235/10654	1.36E-05	0.006339	0.004811	MYC/NOTCH2/JUN/IKZF1/FBXW7/EP300/AKT1
R-HSA-1257604	**PIP3 activates AKT signaling**	7/37	264/10654	2.89E-05	0.006724	0.005104	ERBB2/TSC2/PREX2/PIK3CA/JUN/INSR/AKT1
R-HSA-9006925	**Intracellular signaling by second messengers**	7/37	305/10654	7.23E-05	0.011238	0.008529	ERBB2/TSC2/PREX2/PIK3CA/JUN/INSR/AKT1
R-HSA-8939211	**ESR-mediated signaling**	6/37	223/10654	0.000106	0.012372	0.00939	MYC/PTK2/PIK3CA/JUN/EP300/AKT1
R-HSA-9013694	Signaling by NOTCH4	4/37	82/10654	0.000177	0.016538	0.012552	NOTCH2/FBXW7/EP300/AKT1
R-HSA-5663202	Diseases of signal transduction	7/37	377/10654	0.00027	0.018733	0.014218	MYC/ERBB2/TSC2/PIK3CA/FBXW7/EP300/AKT1
R-HSA-8864260	Transcriptional regulation by the AP-2 (TFAP2) family of transcription factors	3/37	38/10654	0.000299	0.018733	0.014218	MYC/ERBB2/EP300
R-HSA-2219528	PI3K/AKT Signaling in Cancer	4/37	101/10654	0.000395	0.018733	0.014218	ERBB2/TSC2/PIK3CA/AKT1
R-HSA-186712	Regulation of beta-cell development	3/37	42/10654	0.000403	0.018733	0.014218	HNF1A/EP300/AKT1
R-HSA-6811558	PI5P, PP2A and IER3 Regulate PI3K/AKT Signaling	4/37	103/10654	0.000426	0.018733	0.014218	ERBB2/PIK3CA/INSR/AKT1
R-HSA-5683057	MAPK family signaling cascades	6/37	294/10654	0.000474	0.018733	0.014218	MYC/ERBB2/SPTA1/PTK2/JUN/ETV4
R-HSA-9006931	Signaling by Nuclear Receptors	6/37	299/10654	0.000519	0.018733	0.014218	MYC/PTK2/PIK3CA/JUN/EP300/AKT1
R-HSA-199418	Negative regulation of the PI3K/AKT network	4/37	110/10654	0.000546	0.018733	0.014218	ERBB2/PIK3CA/INSR/AKT1
R-HSA-2122947	NOTCH1 Intracellular Domain Regulates Transcription	3/37	47/10654	0.000563	0.018733	0.014218	MYC/FBXW7/EP300
R-HSA-1227986	Signaling by ERBB2	3/37	50/10654	0.000675	0.020256	0.015374	ERBB2/PIK3CA/AKT1
R-HSA-2197563	NOTCH2 intracellular domain regulates transcription	2/37	12/10654	0.000758	0.020256	0.015374	NOTCH2/EP300
R-HSA-1358803	Downregulation of ERBB2:ERBB3 signaling	2/37	13/10654	0.000894	0.020256	0.015374	ERBB2/AKT1
R-HSA-1500931	Cell-Cell communication	4/37	129/10654	0.000994	0.020256	0.015374	PTK2/PIK3CA/MAGI2/CDH2
R-HSA-198323	AKT phosphorylates targets in the cytosol	2/37	14/10654	0.00104	0.020256	0.015374	TSC2/AKT1
R-HSA-2644602	Signaling by NOTCH1 PEST Domain Mutants in Cancer	3/37	58/10654	0.001043	0.020256	0.015374	MYC/FBXW7/EP300

***Supplementary Table 9*. Top 20 enriched Reactomes from selected sensitive (a) or resistant (b) sub-clone genes.**

Reactomes depicted in Figure 5b,c are shown in bold. Note that some immune-related pathways are enriched only among sensitive sub-clones. Significance calculated with hypergeometric test, FDR corrected by Benjamini-Hochberg procedure. GeneRatio, ratio of input genes that are annotated in a term; BgRatio, ratio of all genes that are annotated in this term.

Version 1.3

Confidential

Supplementary Note

TITLE: A phase Ib/II study of first line pembrolizumab in combination with trastuzumab, capecitabine, and cisplatin in HER2 positive gastric cancer

IRB NUMBER: 4-2016-0190

Clinicaltrials.gov Number: NCT02901301

Confidential

1.0 TRIAL SUMMARY

Abbreviated Title	A phase IB/II study of first line pembrolizumab in combination with trastuzumab, capecitabine, and cisplatin in HER2 positive gastric cancer
Trial Phase	phase IB/II
Clinical Indication	HER2 positive gastric cancer patients
Principal Investigator	Name: Hyun Cheol Chung (M.D, Ph.D.)<br>Address:50-1 Yonsei-ro, Seodaemungu, Seoul 03722<br>Country: Korea<br>Phone no.: [redacted]<br>Fax:[redacted]<br>E-Mail: [redacted]
Contact Information	Name: Hyo Song Kim (M.D.,Ph.D.)<br>Address:50-1 Yonsei-ro, Seodaemungu, Seoul 03722<br>Country: Korea<br>Phone no.:[redacted]<br>Fax:[redacted]<br>E-Mail: [redacted]
Trial Type	Interventional
Type of control	Single arm
Trial Blinding	Open label

Background and Rationale

Gastric cancer is one of the major health problems worldwide, and one of the leading cause of death especially in Asia. Though the cytotoxic chemotherapy is the main treatment option, newer and molecularly targeted agents are recently incorporated to improve the survival outcome. Human epidermal growth factor receptor 2 (HER2, ErbB2) is a transmembrane tyrosine kinase receptor and is overexpressed or amplified in 10-20% of gastric cancer. Recently, Trastuzumab for Gastric Cancer (ToGA) study reported the clinical benefit of trastuzumab for HER2 positive gastric cancer patients. However, because the majority of patients develop intrinsic or acquired resistance within 1 year, elucidating the molecular mechanisms for trastuzumab resistance is warranted to improve the survival outcome of HER2 positive gastric cancer patients.

A growing body of preclinical and clinical evidence shows that the immune system contributes substantially to the therapeutic effects of “monoclonal antibody, trastuzumab”

in solid tumors. In addition, there is strong rationale in exploring the impact of combining trastuzumab with anti-PD-1 inhibitor in HER2 positive cancer.

Pembrolizumab is a potent and highly selective humanized monoclonal antibody designed to directly block the interaction between PD-1 and its ligands, PD-L1 and PD-L2. Pembrolizumab showed anti-tumor effect in gastric cancer. In KN012 study for heavily treated gastric cancer, the overall response rate (ORR) was 30.8% (95% confidence interval (CI) (17.0%, 47.6%) and disease control rate was 43.6% (95% CI (27.8%, 60.4%). Therefore, pembrolizumab was generally tolerated with promising efficacy in gastric cancer.

Based on strong rationale in exploring the impact of combining trastuzumab with anti-PD-1 inhibitor in HER2 positive cancer, we suggest multicenter phase IB/II study to determine antitumor activity and safety of pembrolizumab in combination with standard treatment (trastuzumab, capecitabine, and cisplatin) in patients with HER2 positive gastric cancer. We will also explore predictive biomarker by repeated biopsies and high throughput genetic analyses.

Objectives	**Primary Objective**<br>Ph IB: To determine the recommended dose of phase II (RP2D)<br>Ph II: To evaluate the objective response rate using RECIST 1.1<br>(Supportive analyses include irRECIST)<br><br>**Secondary Objective**<br>1) Duration of response (including time frame: 1, 2 year)<br>2) Time to response<br>3) Progression free survival<br>4) Overall survival<br>5) Safety<br><br>**Exploratory Objective**<br>To explore the molecular mechanisms connecting HER2 activity and immune checkpoint pathway as predictive biomarkers<br>1) To investigate the biomarkers predicting response utilizing tumor tissue

	- Gene expression profiling using NanoString nCounter<br>- Clinical relevance of immune reaction and/or HER2 signaling with companion diagnostics<br>- Exome sequencing (for outlier cases)<br><br>2) To explore specific immune markers using blood<br>- Lymphocyte subset analysis (FACS)<br>- NK cell activity (for crosstalk between NK induced ACDD and immune checkpoint)

Study Design

Open-label, single-arm, multicenter, phase IB/II tril

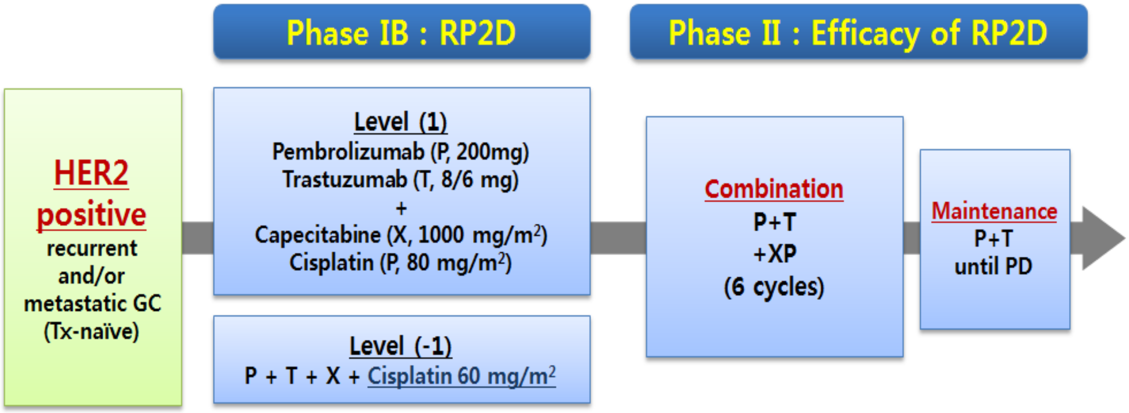

Phase IB part

About 3-6 patients enrollment is expected (dose level 1 and -1). For each dose level, DLTs are assessed during the first 3 weeks for 3 cases. The RP2D will be defined as the highest dose at which fewer than one out of 3 patients (or 2 out of 6) experience a DLT. If a DLT is observed in three patients, the cohort will be expanded a total of 6 patients before further dose escalation. If 2 or more DLTs are observed in three patients for level 1, the cohort will be deescalated to a lower level (level-1).

Phase II part

For the RP2D dose level in phase I, we will expand phase 2 study for a total of 38 patients as RP2D (3 or more patients with MTD dose in phase I). Finally, about 38~44 patients will be enrolled for this sequential phase IB/II trial. Patients will be treated at the time of disease progression, toxicities, or patient's refusal

Treatment

Drug	Dose/ Potency	Frequency	Treatment Period (Q3 weeks)	Route of Administration	Use
**Pembrolizumab**	200 mg	Q3W	Day 1	IV infusion	Experimental
**Trastuzumab**	6 mg/kg (8mg loading dose)	Q3W	Day 1	IV infusion	
**Capecitabine**	1,000 mg/m^2	BID	Day 1-14	p.o.	
**Cisplatin**	RP2D	Q3W	Day 1	IV infusion	

Inclusion Criteria

1. HER2 positive advanced gastric cancer

   HER2-positive tumor defined as either IHC 3+ or IHC 2+ in combination with

   ISH + (or FISH+), as assessed by local laboratory on primary or metastatic tumor

2. Be willing and able to provide written informed consent/assent for the trial.
3. Be ≥ 19 years of age on day of signing informed consent.
4. Have measurable disease based on RECIST 1.1.
5. Be willing to provide tissue from an endoscopic or excisional biopsy of a tumor lesion.
6. Have a performance status of 0 or 1 on the ECOG Performance Scale.
7. Demonstrate adequate organ function
8. Female subject of childbearing potential should have a negative urine or serum pregnancy or be willing to use birth control.

Exclusion Criteria
1. Is currently participating and receiving study therapy or has participated in a study of an investigational agent and received study therapy or used an investigational device within 4 weeks of the first dose of treatment.<br>2. Has a diagnosis of immunodeficiency or is receiving systemic steroid therapy or any other form of immunosuppressive therapy within 7 days prior to the first dose of trial treatment.<br>3. Has a known history of active TB (Bacillus Tuberculosis)<br>4. Hypersensitivity to pembrolizumab or any of its excipients.<br>5. Has had a prior anti-cancer monoclonal antibody (mAb) within 4 weeks prior to study Day 1 or who has not recovered (i.e., ≤ Grade 1 or at baseline) from adverse events due to agents administered more than 4 weeks earlier.<br>6. Has had prior chemotherapy, targeted small molecule therapy, or radiation therapy within 2 weeks prior to study Day 1 or who has not recovered (i.e., ≤ Grade 1 or at baseline) from adverse events due to a previously administered agent.<br>7. Has a known additional malignancy that is progressing or requires active treatment within 3 years. Exceptions include basal cell carcinoma of the skin, squamous cell carcinoma of the skin and thyroid cancer that has undergone potentially curative therapy or in situ cervical cancer.<br>8. Has known active central nervous system (CNS) metastases and/or carcinomatous meningitis.<br>9. Has active autoimmune disease that has required systemic treatment in the past 2 years<br>10. Has known history of, or any evidence of active, non-infectious pneumonitis.<br>11. Has an active infection requiring systemic therapy.<br>12. Has known psychiatric or substance abuse disorders that would interfere with cooperation with the requirements of the trial.

13. Is pregnant or breastfeeding, or expecting to conceive or father children within the projected duration of the trial, starting with the pre-screening or screening visit through 120 days after the last dose of trial treatment.<br>14. Has received prior therapy with an anti-PD-1, anti-PD-L1, or anti-PD-L2 agent.<br>15. Has a known history of Human Immunodeficiency Virus (HIV) (HIV 1/2 antibodies).<br>16. Has known active Hepatitis B (HBsAg reactive and HBV DNA is detected) or Hepatitis C (anti-HCV reactive and HCV RNA [qualitative] is detected).<br>17. Has received a live vaccine within 30 days of planned start of study therapy.	
Number of trial subjects	38~44
Estimated enrollment period	30 months
Duration of participation	Oct 2016 – Apr 2019
Estimated duration of trial	Oct 2016 – Oct 2019<br>(6 months follow up after last enrollment)

Table of Contents

1.0 TRIAL SUMMARY 2

2.0 TRIAL DESIGN 12

2.1 Trial Design 12

2.2 Trial Diagram 12

3.0 OBJECTIVE(S) & HYPOTHESIS(ES) 12

3.1 Primary Objective(s) & Hypothesis(es) 12

3.2 Secondary Objective(s) & Hypothesis(es) 13

3.3 Exploratory Objective 13

4.0 BACKGROUND & RATIONALE 13

4.1 Background 13

4.1.1 Pharmaceutical and Therapeutic Background 14

4.1.2 Preclinical and Clinical Trial Data 15

4.2 Rationale 15

4.2.1 Rationale of Pembrolizumab for the Gastric Cancer 15

4.2.2 Rationale of HER2 Positive Gastric Cancer 16

4.2.3 Rationale of Pembrolizumab for HER2 Positive Gastric Cancer 17

4.2.4 Rationale for Dose Selection/Regimen/Modification 19

4.2.5 Rationale for Endpoint 21

5.0 METHODOLOGY 24

5.1 Entry Criteria 24

5.1.1 Diagnosis/Condition for Entry into the Trial 24

5.1.2 Subject Inclusion Criteria 24

5.1.3 Subject Exclusion Criteria 26

5.2 Trial Treatments 28

5.2.1 Dose Selection/Modification 30

5.2.2 Timing of Dose Administration 36

5.2.3 Trial Blinding/Masking 37

5.3 Concomitant Medications/Vaccinations (allowed & prohibited) 37

5.3.1 Acceptable Concomitant Medications 38

5.3.2 Prohibited Concomitant Medications 38

5.4 Rescue Medications & Supportive Care 39

5.4.1 Supportive Care Guidelines for Pembrolizumab 39

5.4.2 Supportive Care Guidelines for Trastuzumab 44

5.4.3 Supportive Care Guidelines for Cisplatin 44

5.5 Diet/Drug/Activity/Other Considerations 44

5.5.1 Diet and drug 44

5.5.2 Contraception 44

5.5.3 Use in Pregnancy 45

5.5.4 Use in Nursing Women 45

5.6 Subject Withdrawal/Discontinuation Criteria 46

5.6.1 Discontinuation of Study Therapy after CR 47

5.7 Subject Replacement Strategy 47

5.8 Clinical Criteria for Early Trial Termination 47

6.0 TRIAL FLOW CHART 48

6.1 Study Flow Chart 48

7.0 TRIAL PROCEDURES 52

7.1 Trial Procedures 52

7.1.1 Administrative Procedures 52

7.1.2 Clinical Procedures/Assessments 54

7.1.3 Laboratory Procedures/Assessments 57

7.1.4 Other Procedures 60

7.1.5 Visit Requirements 61

7.1.6 Reporting of Pregnancy and Lactation to the Sponsor and to Merck 63

7.1.7 Immediate Reporting of Adverse Events to the Sponsor and to Merck 64

7.1.8 Evaluating Adverse Events 66

7.1.9 Responsibility for Reporting Adverse Events 71

8.0 STATISTICAL ANALYSIS PLAN 71

8.1 Statistical Analysis Plan Summary 71

8.2 Statistical Analysis Plan 71

9.0 LABELING, PACKAGING, STORAGE AND RETURN OF CLINICAL SUPPLIES 72

9.1 Investigational Product 72

9.2 Packaging and Labeling Information 73

9.3 Clinical Supplies Disclosure 73

9.4 Storage and Handling Requirements 73

9.5 Returns and Reconciliation 73

10.0 ADMINISTRATIVE AND REGULATORY DETAILS 74

10.1 Confidentiality 74

10.2 Compliance with Financial Disclosure Requirements 74

10.3 Compliance with Law, Audit and Debarment 74

10.4 Compliance with Trial Registration and Results Posting Requirements 75

10.5 Quality Management System ... 75

10.6 Data Management ... 76

11.0 APPENDICES ... 77

11.1 ECOG Performance Status ... 77

11.2 Common Terminology Criteria for Adverse Events V4.03 (CTCAE) ... 77

11.3 Response Evaluation Criteria in Solid Tumors (RECIST) 1.1 Criteria for Evaluating Response in Solid Tumors ... 78

11.4 Events of Clinical Interest Guidance Document ... 78

11.5 References ... 78

2.0 TRIAL DESIGN

2.1 Trial Design

IB: 3+3 dose escalation design, single center

II: single-arm, open label, multi-center

2.2 Trial Diagram

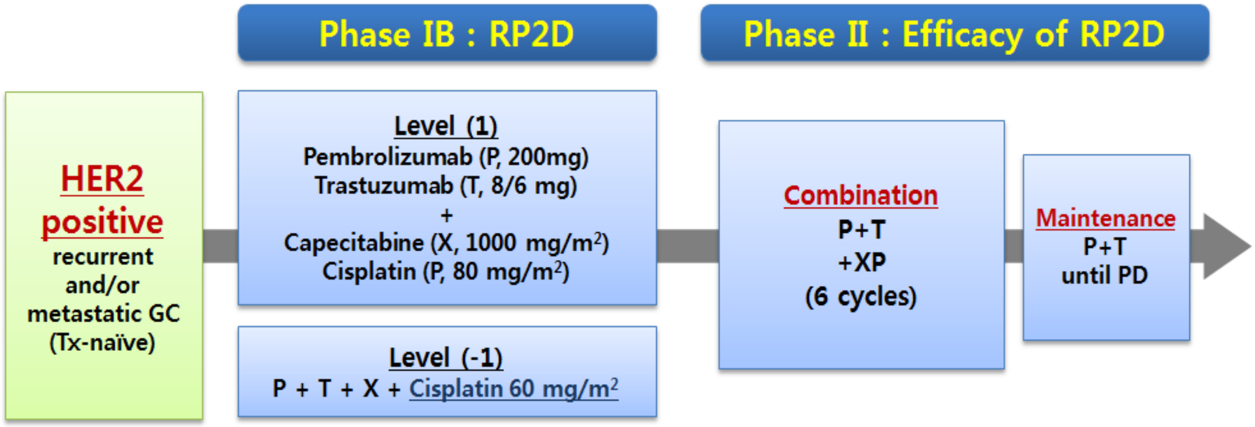

3.0 OBJECTIVE(S) & HYPOTHESIS(ES)

3.1 Primary Objective(s) & Hypothesis(es)

(1) Ph IB: To determine the recommended dose of phase II (RP2D) of pembrolizumab 200 mg administered in combination with cisplatin, capecitabine, and trastuzumab in HER2 positive recurrent and/or metastatic gastric or gastroesophageal junction adenocarcinoma

(2) Ph II: To evaluate the response rate of pembrolizumab using RECIST 1.1

Hypothesis: Pembrolizumab in combination with capecitabine, cisplatin, trastuzumab is tolerable and synergistic for HER2 positive advanced gastric cancer

3.2 Secondary Objective(s) & Hypothesis(es)

(1) Ph IB: To evaluate safety

(2) Ph II: To evaluate duration of response (DOR), progression free survival (PFS), overall survival (OS), and safety

Hypothesis: Pembrolizumab in combination with capecitabine, cisplatin, trastuzumab is synergistic for HER2 positive advanced gastric cancer

Supportive analyses include irRECIST evaluation of ORR as well as RECIST and irRECIST evaluation of DOR (among responders), DCR, PFS and OS.

3.3 Exploratory Objective

1) To investigate the biomarkers predicting response utilizing tumor tissue

- Gene expression profiling using NanoString nCounter
- Clinical relevance of immune reaction and/or HER2 signaling with companion diagnostics (ex, immunohistochemistry for PD-L1)
- Exome sequencing (only for outlier cases)

2) To explore specific immune markers using blood

- Lymphocyte subset analysis (FACS)
- NK cell activity (for crosstalk between NK induced ACDD and immune checkpoint)

4.0 BACKGROUND & RATIONALE

4.1 Background

Refer to the Investigator's Brochure (IB)/approved labeling for detailed background information on MK-3475.

4.1.1 Pharmaceutical and Therapeutic Background

The importance of intact immune surveillance in controlling outgrowth of neoplastic transformation has been known for decades. Accumulating evidence shows a correlation between tumor-infiltrating lymphocytes (TILs) in cancer tissue and favorable prognosis in various malignancies. In particular, the presence of CD8+ T-cells and the ratio of CD8+ effector T-cells / FoxP3+ regulatory T-cells seems to correlate with improved prognosis and long-term survival in many solid tumors.

The PD-1 receptor-ligand interaction is a major pathway hijacked by tumors to suppress immune control. The normal function of PD-1, expressed on the cell surface of activated T-cells under healthy conditions, is to down-modulate unwanted or excessive immune responses, including autoimmune reactions. PD-1 (encoded by the gene Pdcd1) is an Ig superfamily member related to CD28 and CTLA-4 which has been shown to negatively regulate antigen receptor signaling upon engagement of its ligands (PD-L1 and/or PD-L2). The structure of murine PD-1 has been resolved. PD-1 and family members are type I transmembrane glycoproteins containing an Ig Variable-type (V-type) domain responsible for ligand binding and a cytoplasmic tail which is responsible for the binding of signaling molecules. The cytoplasmic tail of PD-1 contains 2 tyrosine-based signaling motifs, an immunoreceptor tyrosine-based inhibition motif (ITIM) and an immunoreceptor tyrosine-based switch motif (ITSM). Following T-cell stimulation, PD-1 recruits the tyrosine phosphatases SHP-1 and SHP-2 to the ITSM motif within its cytoplasmic tail, leading to the dephosphorylation of effector molecules such as CD3ζ, PKCθ and ZAP70 which are involved in the CD3 T-cell signaling cascade. The mechanism by which PD-1 down modulates T-cell responses is similar to, but distinct from that of CTLA-4 as both molecules regulate an overlapping set of signaling proteins. PD-1 was shown to be expressed on activated lymphocytes including peripheral CD4+ and CD8+ T-cells, B-cells, T regs and Natural Killer cells. Expression has also been shown during thymic development on CD4-CD8- (double negative) T-cells as well as subsets of macrophages and dendritic cells. The ligands for PD-1 (PD-L1 and PD-L2) are constitutively expressed or can be induced in a variety of cell types, including non-hematopoietic tissues as well as in various tumors . Both ligands are type I transmembrane receptors containing both IgV- and IgC-like domains in the extracellular region and contain short cytoplasmic regions with no known signaling motifs. Binding of either PD-1 ligand to PD-1 inhibits T-cell activation triggered through the T-cell receptor. PD-L1 is expressed at low levels on various non-hematopoietic tissues, most notably on vascular endothelium, whereas PD-L2 protein is only detectably expressed on antigen-presenting cells found in lymphoid tissue or chronic inflammatory environments. PD-L2 is thought to control immune T-cell activation in lymphoid organs, whereas PD-L1 serves to dampen unwarranted T-cell function in peripheral tissues. Although healthy organs express little (if any) PD-L1, a variety of cancers were demonstrated to express abundant levels of this T-cell inhibitor. PD-1 has

been suggested to regulate tumor-specific T-cell expansion in subjects with melanoma (MEL). This suggests that the PD-1/PD-L1 pathway plays a critical role in tumor immune evasion and should be considered as an attractive target for therapeutic intervention.

Pembrolizumab is a potent and highly selective humanized monoclonal antibody (mAb) of the IgG4/kappa isotype designed to directly block the interaction between PD-1 and its ligands, PD-L1 and PD-L2. Keytruda™ (pembrolizumab) has recently been approved in the United Stated for the treatment of patients with unresectable or metastatic melanoma and disease progression following ipilumumab and, if BRAF V600 mutation positive, a BRAF inhibitor.

4.1.2 Preclinical and Clinical Trial Data

Refer to the Investigator's Brochure for Preclinical and Clinical data.

4.2 Rationale

4.2.1 Rationale of Pembrolizumab for the Gastric Cancer

The importance of intact immune surveillance in controlling outgrowth of neoplastic transformation has been known for decades. The programmed death 1 protein (PD-1) is key immune checkpoint receptor expressed by activated T cells. PD-1 binds to its ligands PD1-L1 (B7-H1) and PD1-L2 (B7-DC), which are expressed on tumor cells, thereby causing immunosuppression and preventing the immune system from rejecting the tumor. The ligands for PD-1 (PD-L1 and PD-L2) are constitutively expressed or can be induced in a variety of cell types, including non-hematopoietic tissues as well as in various tumor.[1,2)] In gastric cancer PD-L1 and PD-L2 overexpression have recently been associated with EBV positive tumors.[3)] Monoclonal antibodies targeting both PD-1 and PD-L1 are being developed to interrupt this pathway and to augment the antitumor immune response; these have demonstrated significant clinical activity against several tumor types.

Pembrolizumab is a potent and highly selective humanized monoclonal antibody of the IgG4/kappa isotype designed to directly block the interaction between PD-1 and its ligands, PD-L1 and PD-L2. Pembrolizumab showed anti-tumor effect in not only melanoma, but in several tumor types. Especially, expression of PD-L1 by the tumor appeared to predict for a higher response for Pembrolizumab. Ongoing clinical trials are being conducted in advanced melanoma, non -small cell lung cancer, a number of advanced solid tumor indications and hematologic malignancies. For study details please refer to the IB. Preliminary interim data is available from a cohort of gastric adenocarcinoma patients studied in trial KN012[4)]. Thirty-nine patients (19 from clinical trial sites in Asia and 20 from trial sites outside Asia) who had metastatic gastric or gastroesophageal adenocarcinoma, ECOG performance

status of 0 or 1, and tumor positive for PD-L1 by immunohistochemistry (defined as staining in ≥1% of tumor cells or any stroma cells using a prototype assay) received single agent pembrolizumab at a dose of 10mg/kg every 2 weeks. The number of prior systemic treatments for metastatic disease ranged from zero to greater than 4. The primary efficacy endpoint was objective response rate (ORR). Overall, the interim ORR is 30.8% (95% confidence interval (CI) (17.0%, 47.6%); all partial responses), while the interim disease control rate (DCR) is 43.6% (95% CI (27.8%, 60.4%). ORR was similar in patients from Asia and outside of Asia, while the DRC was numerically higher in Asia. Responses were observed across all lines of treatment. It should be noted that in the non-Asia group, patients had less prior therapy relative to the Asian patients, and that ORR in later line patients (≥3L) was higher in the Asian group (1 PR/7 patients in the non-Asia group, 4 PR/13 patients in the Asia group). As of the data cutoff (6 Aug 2014), the overall median duration of follow-up is 6-months, and 11/12 patients who responded are still responding. Based on the study, combination trials with 5-Flurorauracil/cisplatin and taxol are ongoing (KN059 and 61).

In conclusion, single agent pembrolizumab at 10 mg/kg Q2W was generally well tolerated in heavily treated and severity and frequency of adverse events similar to that observed in other indications (see the IB for information about adverse events in other indications) with promising efficacy.

4.2.2 Rationale of HER2 Positive Gastric Cancer

The human epidermal growth factor receptor 2 (HER2) is a transmembrane glycoprotein with intrinsic tyrosine kinase activity. Studies of HER2 positivity rates in gastric cancer using immunohistochemistry (IHC) have shown a mean value of 17.6% (range, 6.8%-34%)[5,6)].

ToGA was a randomized, open-label, multicenter, international, comparative Phase III trial designed to evaluate the efficacy and safety of trastuzumab in combination with chemotherapy compared with chemotherapy alone as first-line therapy in patients with inoperable, locally advanced or recurrent and/or metastatic HER2-positive adenocarcinoma of the stomach or gastroesophageal junction.[7)] The primary objective of the study was to compare overall survival for patients treated with trastuzumab combined with fluoropyrimidine (5-FU or capecitabine) plus cisplatin. The results from ToGA demonstrated a significant clinical benefit when trastuzumab was used in combination with chemotherapy in patients with gastric cancer. Overall survival, the primary endpoint, was significantly improved in the trastuzumab plus chemotherapy arm compared with the chemotherapy alone
arm ($p = 0.0045$, log-rank test; hazard ratio, 0.74). The median survival time was 13.8 months in the trastuzumab plus chemotherapy arm and 11.1 months in the chemotherapy alone arm, and the risk of death was decreased by 26% for patients in the trastuzumab plus chemotherapy

arm. All other secondary endpoints demonstrated clinical significance with similar hazard and odds ratios.

As a result of this study, HERCEPTIN® (trastuzumab) is now indicated in the EU and United States, in combination with cisplatin plus capecitabine or 5-FU, for the treatment of patients with HER2-positive metastatic gastric or gastroesophageal junction adenocarcinoma who have not received prior treatment for metastatic disease.

4.2.3 Rationale of Pembrolizumab for HER2 Positive Gastric Cancer

Though the profound efficacy of trastuzumab, majority of patients develop intrinsic or acquired resistance within 1 year. Therefore, novel therapeutic approaches to overcome primary and secondary drug resistance is warranted to improve the survival outcome of HER2 positive gastric cancer. A growing body of preclinical and clinical evidence shows that the immune system contributes substantially to the therapeutic effects of "monoclonal antibody, trastuzumab".[8,9] Because innate and adaptive immune mechanisms are emerging as key players in modulation of the effects of HER2-targeted agents, several immune mechanisms contributing to trastuzumab resistance have been reported.

Clinical trials with HER2 positive breast cancer support the idea that specific characteristics of immune infiltration are associated with a higher likelihood of benefit from trastuzumab. In the NOAH study, increased expression an immunoglobulin metagene was linked to an augmented frequency of pathological complete response to trastuzumab.[10] In the FinHER trial,[11] adjuvant chemotherapy was compared with combined chemotherapy and trastuzumab in HER2 positive breast cancer. In the study, the degree of lymphocyte infiltration was linked to a lower risk of relapse in HER2 positive cohort (figure 1)

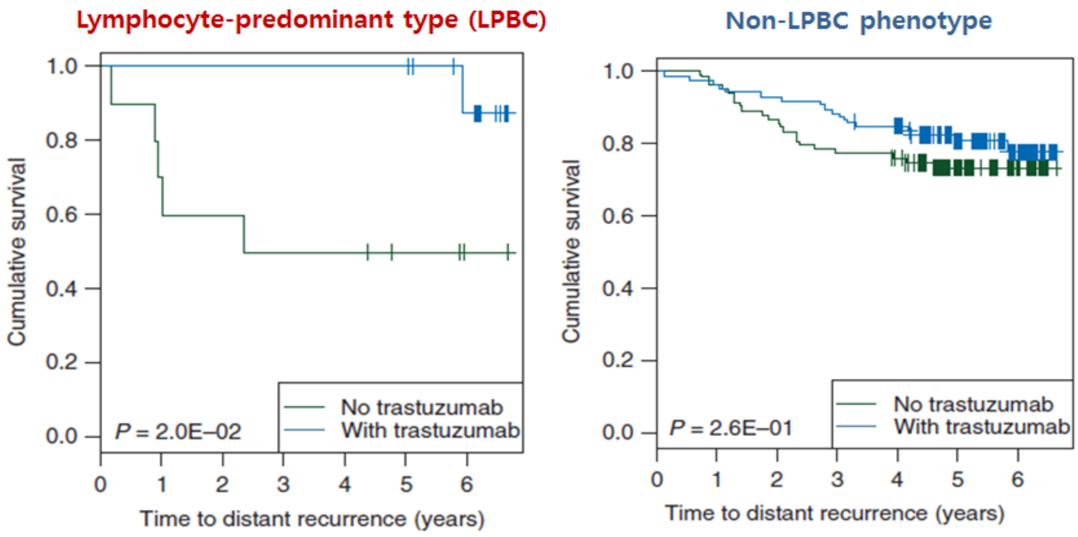

Figure 1. Association of tumor infiltrating lymphocyte with trastuzumab benefit

in HER2+ breast cancer

Prognostic and predictive value of immune gene signature was also reported in trastuzumab neoadjuvant trial for HER2 positive breast cancer.[12)] Overexpression of PD-L1 and CTLA3 were independently associated with lower pathologic response for trastuzumab. Staaf et al identified molecular gene expression profile of HER2 positive breast cancer, and constructed HER2-derived predictive model to define different treatment outcome (figure 2)[13)] The predictor included genes associated with immune response and provided molecular mechanism connecting HER2 activity with immune response. Expression of immune response-associated gene was significantly associated with HER2-derived predictive model.

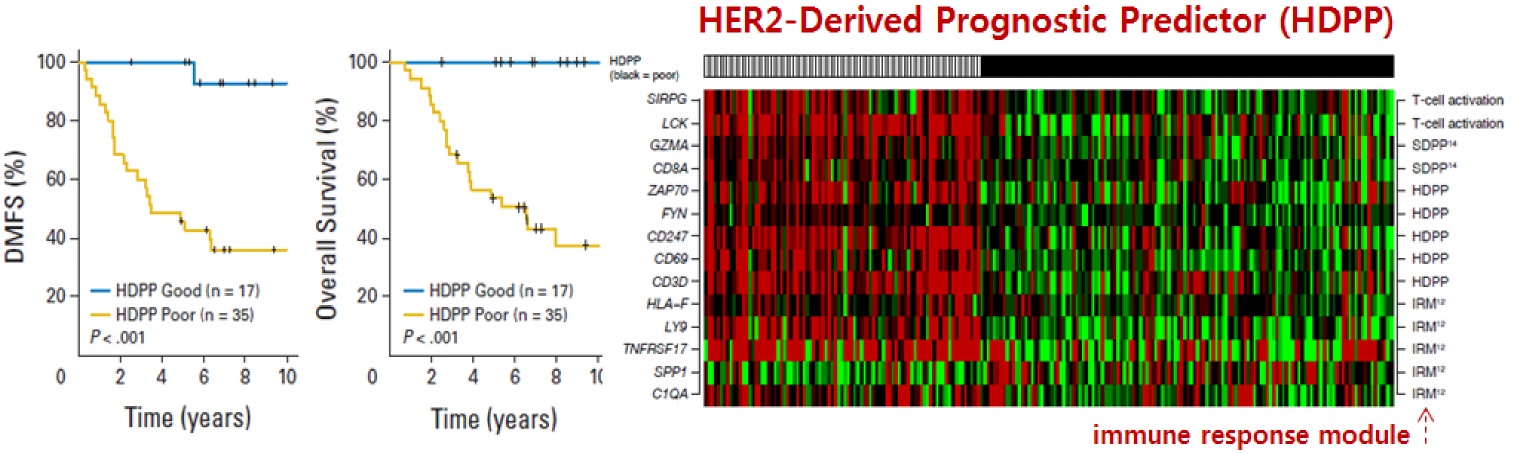

Figure 2. Expression of immune response-associated gene is associated

with HER2-derived predictor model.

Overall, these findings support the involvement of immune checkpoints in the mechanisms of response and resistance to trastuzumab and provide a rationale for combining trastuzumab with immune modulating agents.

Preclinical studies show that monoclonal antibody against PD-1 boost substantially the efficacy of anti-HER2 treatment. Stagg et al demonstrated synergistic activity of anti-PD-1 and trastuzumab (figure 3).[14)] Combining trastuzumab with anti-PD-1 antibody showed greater tumor regression over trastuzumab alone in HER2 positive mouse model.

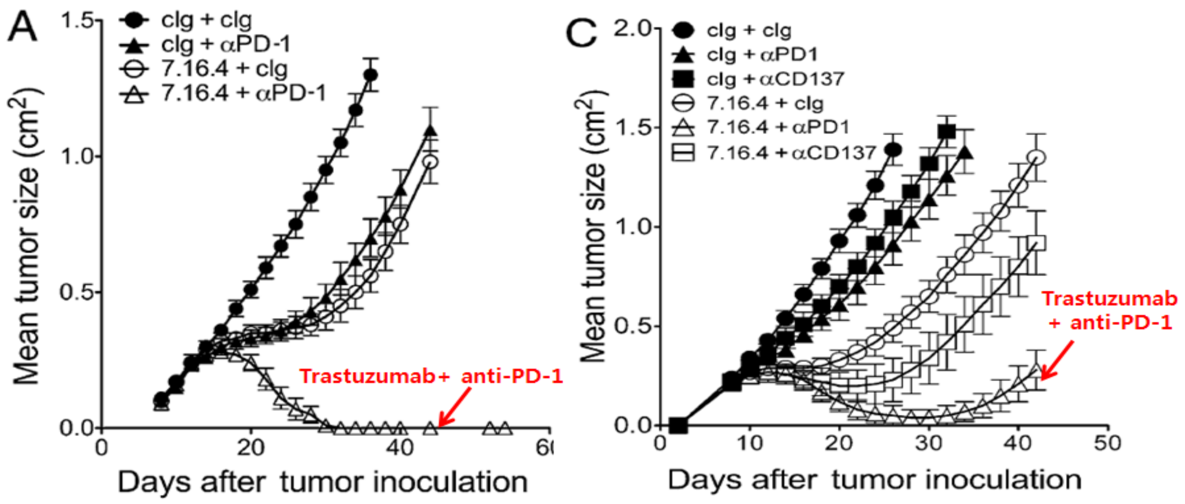

Figure 3. Synergistic activity of anti-PD-1 and trastuzumab in mice model

In conclusion, the role of immune components in the outcome of trastuzumab treatment has well known in HER2 positive breast cancer. In addition, there is strong rationale in exploring the impact of combining trastuzumab with anti-PD-1 inhibitor in HER2 positive cancer.

4.2.4 Rationale for Dose Selection/Regimen/Modification

4.2.4.1 Rationale for Dose Selection: Pembrolizumab

An open-label Phase I trial (Protocol 001) is being conducted to evaluate the safety and clinical activity of single agent MK-3475. The dose escalation portion of this trial evaluated three dose levels, 1 mg/kg, 3 mg/kg, and 10 mg/kg, administered every 2 weeks (Q2W) in subjects with advanced solid tumors. All three dose levels were well tolerated and no dose-limiting toxicities were observed. This first in human study of MK-3475 showed evidence of target engagement and objective evidence of tumor size reduction at all dose levels (1 mg/kg, 3 mg/kg and 10 mg/kg Q2W). No MTD has been identified to date. 10.0 mg/kg Q2W, the highest dose tested in PN001, will be the dose and schedule utilized in Cohorts A, B, C and D of this protocol to test for initial tumor activity. Recent data from other clinical studies within the MK-3475 program has shown that a lower dose of MK-3475 and a less frequent schedule may be sufficient for target engagement and clinical activity.

PK data analysis of pembrolizumab administered Q2W and Q3W showed slow systemic clearance, limited volume of distribution, and a long half-life (refer to IB). Pharmacodynamic data (IL-2 release assay) suggested that peripheral target engagement is

durable (>21 days). This early PK and pharmacodynamic data provides scientific rationale for testing a Q2W and Q3W dosing schedule.

A population pharmacokinetic analysis has been performed using serum concentration time data from 476 patients. Within the resulting population PK model, clearance and volume parameters of pembrolizumab were found to be dependent on body weight. The relationship between clearance and body weight, with an allometric exponent of 0.59, is within the range observed for other antibodies and would support both body weight normalized dosing or a fixed dose across all body weights. Pembrolizumab has been found to have a wide therapeutic range based on the melanoma indication. The differences in exposure for a 200 mg fixed dose regimen relative to a 2 mg/kg Q3W body weight based regimen are anticipated to remain well within the established exposure margins of 0.5 – 5.0 for MK-3475 in the melanoma indication. The exposure margins are based on the notion of similar efficacy and safety in melanoma at 10 mg/kg Q3W vs. the proposed dose regimen of 2 mg/kg Q3W (i.e. 5-fold higher dose and exposure). The population PK evaluation revealed that there was no significant impact of tumor burden on exposure. In addition, exposure was similar between the NSCLC and melanoma indications. Therefore, there are no anticipated changes in exposure between different indication settings.

The rationale for further exploration of 2 mg/kg and comparable doses of pembrolizumab in solid tumors is based on: 1) similar efficacy and safety of pembrolizumab when dosed at either 2 mg/kg or 10 mg/kg Q3W in melanoma patients, 2) the flat exposure-response relationships of pembrolizumab for both efficacy and safety in the dose ranges of 2 mg/kg Q3W to 10 mg/kg Q3W, 3) the lack of effect of tumor burden or indication on distribution behavior of pembrolizumab (as assessed by the population PK model) and 4) the assumption that the dynamics of pembrolizumab target engagement will not vary meaningfully with tumor type.

The choice of the 200 mg Q3W as an appropriate dose for the switch to fixed dosing is based on simulations performed using the population PK model of pembrolizumab showing that the fixed dose of 200 mg every 3 weeks will provide exposures that 1) are optimally consistent with those obtained with the 2 mg/kg dose every 3 weeks, 2) will maintain individual patient exposures in the exposure range established in melanoma as associated with maximal efficacy response and 3) will maintain individual patients exposure in the exposure range established in melanoma that are well tolerated and safe.

A fixed dose regimen will simplify the dosing regimen to be more convenient for physicians and to reduce potential for dosing errors. A fixed dosing scheme will also reduce complexity in the logistical chain at treatment facilities and reduce wastage.

4.2.4.2 Rationale for Dose Selection: Trastuzumab/Capecitabine/Cisplatin

In ToGA study,[7)] conventional chemotherapy (capecitabine/cisplatin; XP or 5-flurorouracil/cisplatin; FP) was given every 3 weeks for 6 cycles. Regarding XP regimen, Capecitabine 1000 mg/m² was given orally twice a day for 14 days followed by a 1-week rest and Cisplatin 80 mg/m² on day 1 was given by intravenous infusion. Trastuzumab was given by intravenous infusion at a dose of 8 mg/kg on day 1 of the first cycle, followed by 6 mg/kg every 3 weeks until disease progression, unacceptable toxicity, or withdrawal of consent. Based on the result, trastuzumab is indicated in combination with cisplatin plus capecitabine for the treatment of HER2-positive gastric cancer. Since then, clinical trials addressed capecitabine (1000 mg/m²), cisplatin (80 mg/m²), and trastuzumab (8 mg/kg followed by 6 mg) as backbone of conventional treatment targeting HER2 positive gastric cancer. Pertuzumab combination trials with trastuzumab, capecitabine, and cisplatin are ongoing (ClinicalTrials.gov Identifier:NCT01774786, NCT01461057, and NCT02205047).

4.2.5 Rationale for Endpoint

4.2.5.1 Efficacy Endpoints

The primary endpoint of phase IB study is to assess dose limiting toxicity (DLT) of the first cycle. Definition of DLT is as follows

Hematologic DLT

- Grade 4 neutropenia lasting for ≥ 7 days in duration
- Grade 4 neutropenia with fever > 38.5 ^{0}C and/or infection requiring antibiotic or anti-fungal treatment
- Grade 4 thrombocytopenia

Non-hematologic DLT

- Grade 3 or greater toxicity lasting > 48 hours other than anorexia, nausea, diarrhea, and alopecia (despite appropriate supportive care)

The primary efficacy objective of phase II study is to evaluate the anti-tumor activity of pembrolizumab in subjects with advanced gastric cancer. Response rates per RECIST 1.1 as assessed will be used as the primary response rate efficacy endpoint. Objective response rate will also be used as the primary endpoint due to the single-arm design of this study.

However RECIST 1.1 will be adapted to account for the unique tumor response profile seen with immunotherapies such as pembrolizumab. Immunotherapeutic agents such as pembrolizumab may produce antitumor effects by potentiating endogenous cancer-specific immune responses which may be functionally anergic. The response patterns seen with such an approach may extend beyond the typical time course of responses seen with cytotoxic agents, and can manifest a clinical response after an initial increase in tumor burden or even the appearance of new lesions. Standard RECIST criteria may not provide a complete response assessment of immunotherapeutic agents such as pembrolizumab. Therefore, RECIST 1.1 will be used with the following adaptation, outlined in Section 7.1.2.6, termed irRECIST. When feasible, subjects should not be discontinued until progression is confirmed. This allowance to continue treatment despite initial radiologic progression takes into account the observation that some subjects can have a transient tumor flare in the first few months after the start of immunotherapy, but with subsequent disease response. A secondary objective including PFS, OS, and DOR will be also evaluated.

4.2.5.2 Safety Endpoints

The primary safety objective of this trial is to characterize the safety and tolerability of pembrolizumab in combination with capecitabine, cisplatin, and trastuzumab in subjects with HER2 positive gastric cancer. The primary safety analysis will be based on subjects who experienced toxicities as defined by NCI CTCAE 4.03 criteria (Appendix 11.2). Safety will be assessed by quantifying the toxicities and grades experienced including serious adverse events (SAEs) and events of clinical interest (ECIs).

4.2.4.3. Biomarker Research

Additional biomarker research to identify factors important for pembrolizumab and trastuzumab therapy may also be pursued. This research may evaluate factors important for predicting responsiveness or resistance to pembrolizumab therapy and other immunologic targets. Tumor from this study may undergo gene expression profiling using NanoString nCounter exome sequencing, immunohistochemistry, and optionally exome sequecing. Blood samples including whole blood and plasma (including peripheral mononuclear cells; PBMC) may undergo the analysis of lymphocyte subset and NK cell activity.

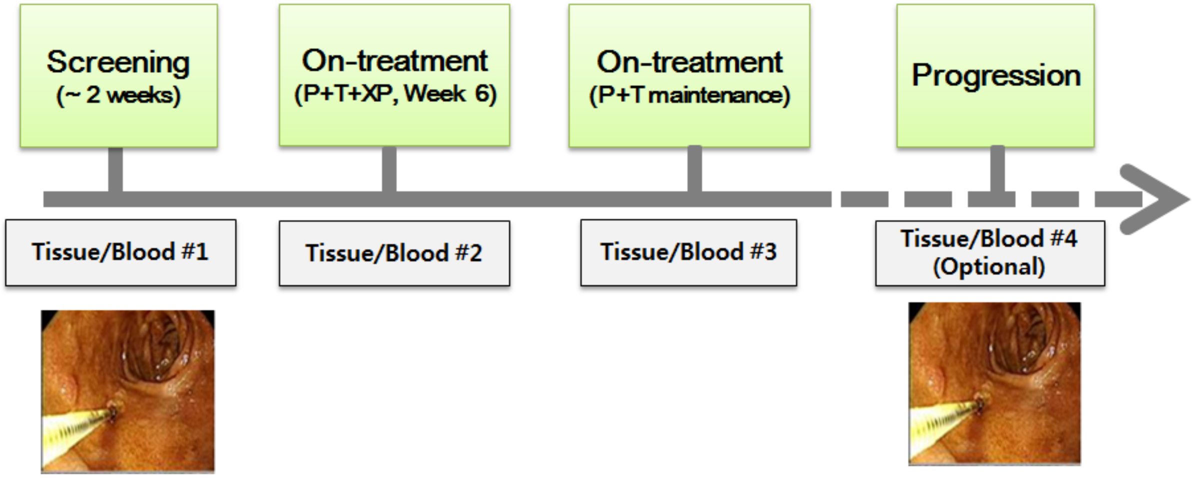

Figure 3. Scheme of Biomarker Research

<u>Tumor tissue</u>

1) Gene expression profiling using NanoString nCounter

- To define the value of baseline immune-related gene signature as predictive biomarker

- To explore whether the defined gene signature is changed as pharmacodynamic marker

: pre-treatment and on-treatment

2) Clinical relevance of immune reaction and/or HER2 signaling with companion diagnostics

- To examine the clinical relevance of baseline immune reaction and/or HER2 signaling as predictive marker

  - Immune modulating activity: Immune checkpoint expression (PD1,PDL1, LAG3, TIM3, CTLA4) using immunohistochemistry (IHC)
  - HER2 signaling pathway :

    EGFR, HER3 expression and amplification (IHC and FISH)

    PIK3CA mutation (exon 9 and 20) and PIK3CA amplification (FISH)

    PTEN loss (IHC)

: pre-treatment and on-treatment

3) Exome sequencing

- Identification of acquired resistance mechanism for outlier cases (for partial responders)

  : pre-treatment and progression

<Blood>

1) Lymphocyte subset analysis (FACS)

- To explore whether specific lymphocyte subsets and/or changes predict response
  - Treg, CD4/8 T cell, CD4/8 T-cell exhaustion, Activated CD4/8 T cell, Effector CD8 T cell, and macrophage

  : pre-treatment, on-treatment and post- treatment

2) NK cell activity

- To explore crosstalk between NK cell induced ADCC and immune checkpoint inhibitor
  - Cancer Immuno-Assay Kit (ATgen, Seoul) for NK cell activity

  : pre-treatment, on-treatment and post- treatment

5.0 METHODOLOGY

5.1 Entry Criteria

5.1.1 Diagnosis/Condition for Entry into the Trial

HER2-positive adenocarcinoma of the stomach or gastroesophageal junction have received no prior treatment for advanced or metastatic gastric cancer.

5.1.2 Subject Inclusion Criteria

In order to be eligible for participation in this trial, the subject must:

1. HER2 positive advanced gastric cancer

   HER2-positive tumor defined as either IHC 3+ or IHC 2+ in combination with

   ISH + (or FISH), as assessed by local laboratory on primary or metastatic tumor

   (ISH positivity is defined as a ratio of ≥ 2.0 for the number of HER2 gene copies to the number of signals for CEP17)

2. Be willing and able to provide written informed consent/assent for the trial.

3. Be ≥ 19 years of age on day of signing informed consent.

4. Have measurable disease based on RECIST 1.1.

5. Be willing to provide tissue from an endoscopic or excisional biopsy of a tumor lesion.

6. Have a performance status of 0 or 1 on the ECOG Performance Scale.

7. Demonstrate adequate organ function as defined in Table 1, all screening labs should be performed within 14 days of treatment initiation.

Table 1. Adequate Organ Function Laboratory Values

System	Laboratory Value
**Hematological**	
Absolute neutrophil count (ANC)	≥1,500 /mcL
Platelets	≥100,000 / mcL
Hemoglobin	≥9 g/dL or ≥5.6 mmol/L without transfusion or EPO dependency (within 7 days of assessment)
**Renal**	
Serum creatinine **OR**<br>Measured or calculated[a] creatinine clearance<br>(GFR can also be used in place of creatinine or CrCl)	≤1.5 X upper limit of normal (ULN) **OR**<br><br>≥60 mL/min for subject with creatinine levels > 1.5 X institutional ULN
**Hepatic**	
Serum total bilirubin	≤ 1.5 X ULN **OR**

	Direct bilirubin ≤ ULN for subjects with total bilirubin levels > 1.5 ULN
AST (SGOT) and ALT (SGPT)	≤ 2.5 X ULN **OR**<br>≤ 5 X ULN for subjects with liver metastases
Albumin	≥2.5 mg/dL
**Coagulation**	
International Normalized Ratio (INR) or Prothrombin Time (PT)	≤1.5 X ULN unless subject is receiving anticoagulant therapy as long as PT or PTT is within therapeutic range of intended use of anticoagulants
Activated Partial Thromboplastin Time (aPTT)	≤1.5 X ULN unless subject is receiving anticoagulant therapy as long as PT or PTT is within therapeutic range of intended use of anticoagulants
[a]Creatinine clearance should be calculated per institutional standard.	

8. Female subject of childbearing potential should have a negative urine or serum pregnancy within 72 hours prior to receiving the first dose of study medication. If the urine test is positive or cannot be confirmed as negative, a serum pregnancy test will be required.

9. Female subjects of childbearing potential should be willing to use 2 methods of birth control or be surgically sterile, or abstain from heterosexual activity for the course of the study through 120 days after the last dose of study medication (Reference Section 5.7.2). Subjects of childbearing potential are those who have not been surgically sterilized or have not been free from menses for > 1 year.

10. Male subjects should agree to use an adequate method of contraception starting with the first dose of study therapy through 120 days after the last dose of study therapy.

5.1.3 Subject Exclusion Criteria

The subject must be excluded from participating in the trial if the subject:

1. Is currently participating and receiving study therapy or has participated in a study of an investigational agent and received study therapy or used an investigational device within 4 weeks of the first dose of treatment.

2. Has a diagnosis of immunodeficiency or is receiving systemic steroid therapy or any other form of immunosuppressive therapy within 7 days prior to the first dose of trial treatment.

3. Has a known history of active TB (Bacillus Tuberculosis)

4. Hypersensitivity to pembrolizumab or any of its excipients.

5. Has had a prior anti-cancer monoclonal antibody (mAb) within 4 weeks prior to study Day 1 or who has not recovered (i.e., ≤ Grade 1 or at baseline) from adverse events due to agents administered more than 4 weeks earlier.

6. Has had prior chemotherapy, targeted small molecule therapy, or radiation therapy within 2 weeks prior to study Day 1 or who has not recovered (i.e., ≤ Grade 1 or at baseline) from adverse events due to a previously administered agent.

    - Note: Subjects with ≤ Grade 2 neuropathy are an exception to this criterion and may qualify for the study.

    - Note: If subject received major surgery, they must have recovered adequately from the toxicity and/or complications from the intervention prior to starting therapy.

7. Has a known additional malignancy that is progressing or requires active treatment within 3 years. Exceptions include basal cell carcinoma of the skin, squamous cell carcinoma of the skin and thyroid cancer that has undergone potentially curative therapy or in situ cervical cancer.

8. Has known active central nervous system (CNS) metastases and/or carcinomatous meningitis. Subjects with previously treated brain metastases may participate provided they are stable (without evidence of progression by imaging for at least four weeks prior to the first dose of trial treatment and any neurologic symptoms have returned to baseline), have no evidence of new or enlarging brain metastases, and are not using steroids for at least 7 days prior to trial treatment. This exception does not include carcinomatous meningitis which is excluded regardless of clinical stability.

9. Has active autoimmune disease that has required systemic treatment in the past 2 years (i.e. with use of disease modifying agents, corticosteroids or immunosuppressive drugs). Replacement therapy (eg., thyroxine, insulin, or physiologic corticosteroid replacement therapy for adrenal or pituitary insufficiency, etc.) is not considered a form of systemic treatment.

10. Has known history of, or any evidence of active, non-infectious pneumonitis.

11. Has an active infection requiring systemic therapy.

12. Has known psychiatric or substance abuse disorders that would interfere with cooperation with the requirements of the trial.

13. Is pregnant or breastfeeding, or expecting to conceive or father children within the projected duration of the trial, starting with the pre-screening or screening visit through 120 days after the last dose of trial treatment.

14. Has received prior therapy with an anti-PD-1, anti-PD-L1, or anti-PD-L2 agent.

15. Has a known history of Human Immunodeficiency Virus (HIV) (HIV 1/2 antibodies).

16. Has known active Hepatitis B (HBsAg reactive and HBV DNA is detected) or Hepatitis C (anti-HCV reactive and HCV RNA [qualitative] is detected).

17. Has received a live vaccine within 30 days of planned start of study therapy.

    Note: Seasonal influenza vaccines for injection are generally inactivated flu vaccines and are allowed; however intranasal influenza vaccines (e.g., Flu-Mist®) are live attenuated vaccines, and are not allowed.

5.2 Trial Treatments

Patients will receive trastuzumab, cisplatin, and capecitabine as defined by the ToGA study. Treatment will be given in 3-week cycles as outlined below. The treatment to be used in this trial is outlined below in Table 2.

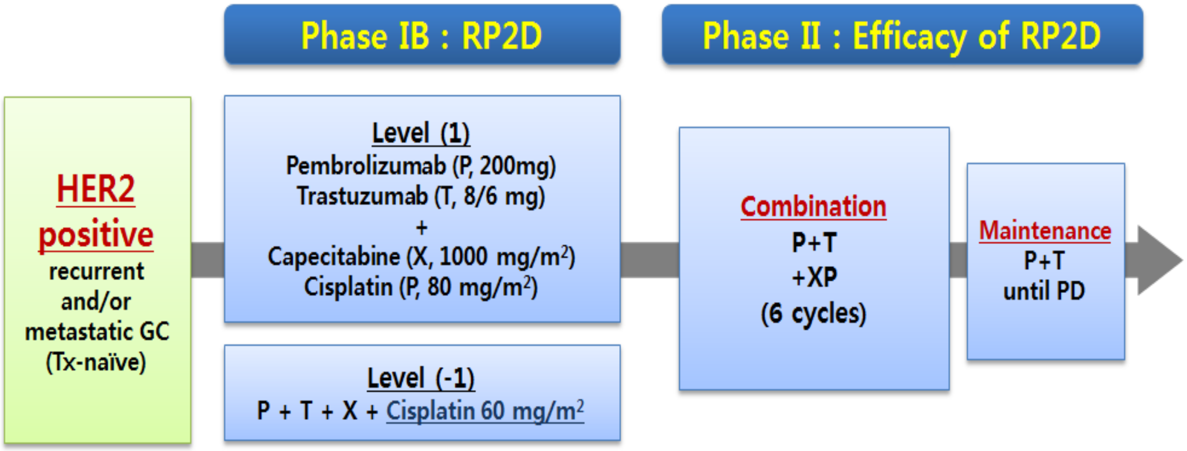

Table 2. Trial Treatment (during phase IB)

	**Level 0**	**Level -1**
**Pembrolizumab**	200 mg fixed dose	200 mg fixed dose
**Trastuzumab (Herzuma)**	6 mg/kg<br>(8mg loading dose)	6 mg/kg<br>(8mg loading dose)
**Capecitabine**	1,000 mg/m^2 bid	1,000 mg/m^2 bid
**Cisplatin**	**80 mg/m^2**	**60 mg/m^2**

Table 3. Trial Treatment (during phase II)

**Drug**	**Dose/ Potency**	**Frequency**	**Treatment Period (Q3 weeks)**	**Route of Administration**	**Use**
**Pembrolizumab**	200 mg	Q3W	Day 1	IV infusion	
**Trastuzumab (Herzuma)**	6 mg/kg (8mg loading dose)	Q3W	Day 1	IV infusion	Experimental
**Capecitabine**	1,000 mg/m^2	BID	Day 1-14	p.o.	

Drug	Dose/ Potency	Frequency	Treatment Period (Q3 weeks)	Route of Administration	Use
**Cisplatin**	RP2D	Q3W	Day 1	IV infusion	

5.2.1 Dose Selection/Modification

5.2.1.1 Dose Selection

The rationale for selection of doses to be used in this trial is provided in Section 4.0 – Background and Rationale. Details on preparation and administration of pembrolizumab (MK-3475) are provided in the Pharmacy Manual.

5.2.1.2 Dose Modification

Reduction or holding of one agent and not the other agents is appropriate if, in the opinion of the Investigator, the toxicity is clearly related to one of the study drugs. If, in the opinion of the Investigator, the toxicity is related to the combination of two agents, both drugs should be reduced or held according to recommended dose modifications. If the toxicity is related to the combination of three agents, all three agents should be reduced or held according to the recommended dose modifications. If one or more study agent(s) are held for toxicity, the schedule for restarting the agent(s) should correspond with the next treatment cycle once the toxicity has resolved according to the recommended guidelines.

If any of the individual study drugs must be delayed for a day or more, all agents should be delayed for the same timeframe. If a patient requires a dose delay of XP components of study treatment for > 3 weeks (i.e., > 6 weeks since start of the previous treatment cycle), due to toxicity, the XP chemotherapy components of the treatment regimen will be permanently discontinued for unacceptable toxicity. Treatment with the trastuzumab and pembrolizumab components may continue per protocol.

Table 4. Dose modifications for Trial Medications (in case of level 1 RP2D: cisplatin 80 mg/m^2)

	Level 0	Level -1	Level -2
**Pembrolizumab**	200 mg fixed dose	Dose reductions are not permitted	Dose reductions are not permitted

**Trastuzumab (Herzuma)**	6 mg/kg (8mg loading dose)	Dose reductions are not permitted	Dose reductions are not permitted
**Capecitabine**	1,000 mg/m^2 bid	75%	50%
**Cisplatin**	80 mg/m^2	75%	50%

5.2. 1.2.1 Dose Modification : capecitabine and cisplatin

Table 5. Dose modification s for hematologic toxicity

**Event**		**Action**
**Grade 1-3**	1st occurrence	Hold until recovery to ≤ grade 1 or baseline.<br>Resume without reduction<br>Consider G-CSF support
	2nd occurrence	Reduce by 1 dose level (DL).<br>Consider G-CSF support
	3rd occurrence	Reduce by 2DL<br>Consider G-CSF support
**Grade 3**<br>**Febrile neutropenia**	1st occurrence	Hold until recovery to ≤ grade 1 or baseline.<br>Reduce by 1DL<br>Consider G-CSF support
	2nd occurrence	Reduce by 2DL<br>Consider G-CSF support
	3rd occurrence	Discontinue

**Grade 4 Febrile neutropenia**	1st occurrence	Reduce by 2DL<br><br>Consider G-CSF support
	2nd occurrence	Discontinue

If AE is considered independently related to a specific drug, reduction or holding one agent and not the other agents is permitted based on the investigator's opinion.

Table 6. Dose modification s for non-hematologic toxicity

**Event**		**Action**
**Grade 3-4 non-hematologic toxicities**	1st occurrence	Hold until recovery to ≤ grade 1 or baseline.<br><br>Reduce by 1 DL
	2nd occurrence	Reduce by 2 DL.
	3rd occurrence	Discontinue
**Grade 2-3 Diarrhea, Mucositis, or Hand-foot syndrome**	1st occurrence	Hold until recovery to ≤ grade 1 or baseline.<br><br>Reduce by 1DL (consider capecitabine)
	2nd occurrence	Reduce by 2DL (consider capecitabine)
	3rd occurrence	Discontinue
**Grade 2-4**<br><br>**Creatinine Increased**	1st occurrence	Hold until recovery to ≤ grade 1 or baseline.<br><br>Reduce by 1DL(consider cisplatin)

**Grade 3-4 ototoxicity or neuropathy**	2nd occurrence	Reduce by 2DL(consider cisplatin)
**Grade 4 Laboratory Adverse Events**	1st occurrence	Hold until recovery to ≤ grade 1 or baseline.<br><br>Reduce by 1DL
	2nd occurrence	Reduce by 2DL

If AE is considered independently related to a specific drug (ie, capecitabine only for hand-foot syndrome), reduction or holding one agent and not the other agents is permitted based on the investigator's opinion.

5.2. 1.2.2 Dose Modification : Pembrolizumab

Adverse events (both non-serious and serious) associated with pembrolizumab exposure may represent an immunologic etiology. These adverse events may occur shortly after the first dose or several months after the last dose of treatment. Pembrolizumab must be withheld for drug-related toxicities and severe or life-threatening AEs as per Table 7 below. See Section 5.4 and Events of Clinical Interest Guidance Document for supportive care guidelines, including use of corticosteroids

Table 7. Dose Modification Guidelines for Drug-Related Adverse Events for Pembrolizumab

**Toxicity**	**Hold Treatment For Grade**	**Timing for Restarting Treatment**	**Discontinue Subject**
Diarrhea/Colitis	2-3	Toxicity resolves to Grade 0-1.	Toxicity does not resolve within 12 weeks of last dose or inability to reduce corticosteroid to 10 mg or less of prednisone or equivalent per day within 12 weeks.
	4	Permanently discontinue	Permanently discontinue
AST, ALT, or Increased Bilirubin	2	Toxicity resolves to Grade 0-1	Toxicity does not resolve within 12 weeks of last dose.
	3-4	Permanently discontinue (see exception below)[1]	Permanently discontinue

Toxicity	Hold Treatment For Grade	Timing for Restarting Treatment	Discontinue Subject
Type 1 diabetes mellitus (if new onset) or Hyperglycemia	T1DM or 3-4	Hold pembrolizumab for new onset Type 1 diabetes mellitus or Grade 3-4 hyperglycemia associated with evidence of beta cell failure.	Resume pembrolizumab when patients are clinically and metabolically stable.
Hypophysitis	2-3	Toxicity resolves to Grade 0-1	Toxicity does not resolve within 12 weeks of last dose or inability to reduce corticosteroid to 10 mg or less of prednisone or equivalent per day within 12 weeks.
	4	Permanently discontinue	Permanently discontinue
Hyperthyroidism	3	Toxicity resolves to Grade 0-1	Toxicity does not resolve within 12 weeks of last dose or inability to reduce corticosteroid to 10 mg or less of prednisone or equivalent per day within 12 weeks.
	4	Permanently discontinue	Permanently discontinue
Hypothyroidism	2-4	Therapy with pembrolizumab can be continued while treatment for the thyroid disorder is instituted	Therapy with pembrolizumab can be continued while treatment for the thyroid disorder is instituted.
Infusion Reaction	3-4	Permanently discontinue	Permanently discontinue
Pneumonitis	2	Toxicity resolves to Grade 0-1	Toxicity does not resolve within 12 weeks of last dose or inability to reduce corticosteroid to 10 mg or less of prednisone or equivalent per day within 12 weeks.
	3-4	Permanently discontinue	Permanently discontinue
Renal Failure or Nephritis	2	Toxicity resolves to Grade 0-1	Toxicity does not resolve within 12 weeks of last dose or inability to reduce corticosteroid to 10 mg or less of prednisone or equivalent per day within 12 weeks.
	3-4	Permanently discontinue	Permanently discontinue
	3 or Severe	Toxicity resolves to Grade 0-1	Toxicity does not resolve within 12 weeks of last dose or inability to reduce corticosteroid to 10

Toxicity	Hold Treatment For Grade	Timing for Restarting Treatment	Discontinue Subject
All Other Drug-Related Toxicity[2]			mg or less of prednisone or equivalent per day within 12 weeks.
	4	Permanently discontinue	Permanently discontinue
**Note: Permanently discontinue for any severe or Grade 3 drug-related AE that recurs or any life-threatening event.**			
[1] For patients with liver metastasis who begin treatment with Grade 2 AST or ALT, if AST or ALT increases by greater than or equal to 50% relative to baseline and lasts for at least 1 week then patients should be discontinued.			
[2] Patients with intolerable or persistent Grade 2 drug-related AE may hold study medication at physician discretion. Permanently discontinue study drug for persistent Grade 2 adverse reactions for which treatment with study drug has been held, that do not recover to Grade 0-1 within 12 weeks of the last dose.			

Dosing interruptions are permitted in the case of medical / surgical events or logistical reasons not related to study therapy (e.g., elective surgery, unrelated medical events, patient vacation, and/or holidays). Subjects should be placed back on study therapy within 3 weeks of the scheduled interruption, unless otherwise discussed with the Sponsor. The reason for interruption should be documented in the patient's study record.

5.2. 1.2.3 Dose Modification : Trastuzumab (Herzuma)

Trastuzumab administration may be delayed to assess or treat AEs, such as cardiac events. However, no dose reduction is permitted for trastuzumab. If the patient misses a dose of trastuzumab for any cycle (i.e., the two sequential administration times are 6 weeks or more apart), a reloading dose of 8 mg/kg of trastuzumab should be given.

- **Dose Delays or Discontinuations due to Cardiac Events**

In this study, all patients must have a baseline LVEF value ≥ 55%, and LVEF is to be monitored at least every 9 weeks during chemotherapy treatment and every 12 weeks during antibody treatment. If symptomatic left ventricular dysfunction develops (NCI CTCAE Grade 3 or 4) with a drop in LVEF consistent with cardiac failure, the patient must discontinue study treatment. Left ventricular dysfunction, whether symptomatic or not, should be treated and followed according to standard medical practice and as follows.

Figure 3. Algorithm for Continuation and Discontinuation of Trastuzumab Based on LVEF

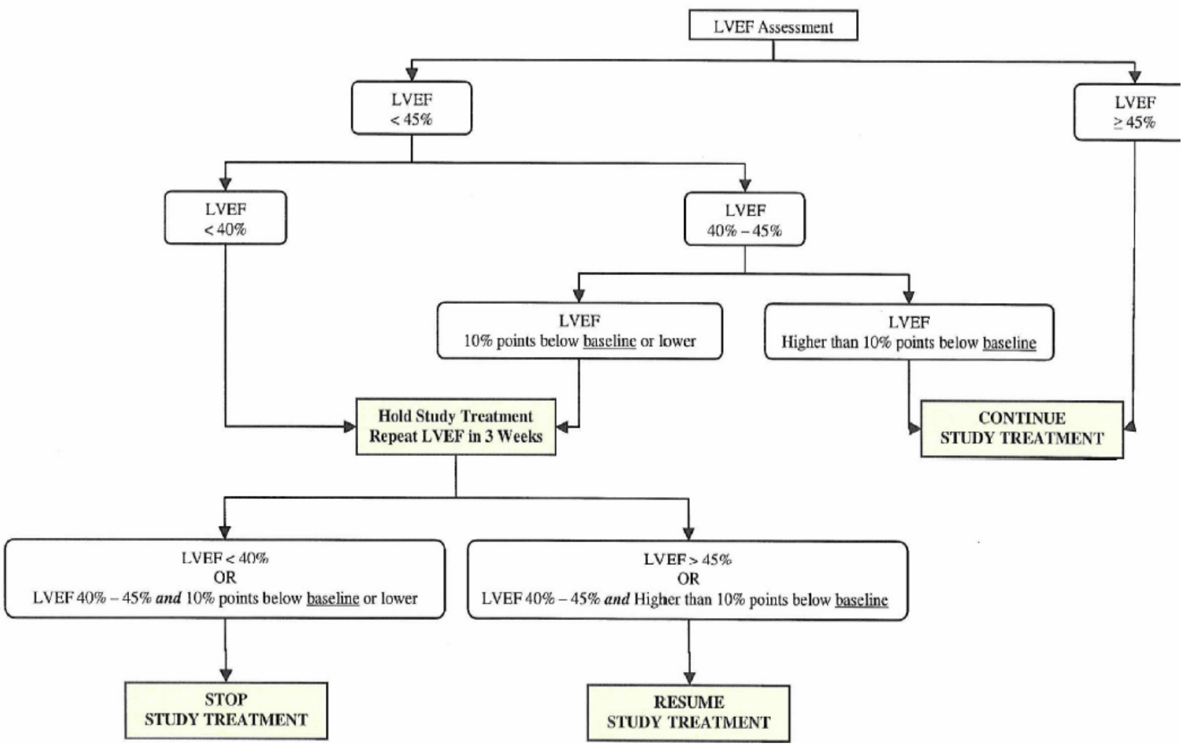

5.2.2 Timing of Dose Administration

All trial treatments will be administered in the order presented below. Pembrolizumab/trastuzumab/cisplatin should be administered on Day 1 of each cycle after all procedures/assessments have been completed as detailed on the Trial Flow Chart (Section 6.0). Pembrolizumab/trastuzumab/cisplatin may be administered up to 3 days before or after the scheduled Day 1 of each cycle due to administrative reasons.

Note: Dosing of each drugs may be withheld in the case of medical / surgical events or logistical reasons (i.e. elective surgery, unrelated medical events, subject vacation, holidays) not related to study therapy. Subjects should be placed back on study therapy within 3 weeks of the scheduled interruption to remain aligned with the Q3W dosing interval. The reason for withholding dosing of either pembrolizumab or combination chemotherapy regimen should be documented in the subject's study record.

- Pembrolizumab 200 mg will be administered as a 30 minute IV infusion every 3 weeks. Sites should make every effort to target infusion timing to be as close to 30 minutes as possible. However, given the variability of infusion pumps from site to site, a

window of -5 minutes and +10 minutes is permitted (i.e., infusion time is 30 minutes: -5 min/+10 min).

- Trastuzumab will be administered as an intravenous (IV) infusion on Day 1 of each cycle flowing administration of pembrolizumab, at a loading dose of 8 mg/kg for Cycle 1 and a dose of 6 mg/kg for subsequent cycles.

- Cisplatin 80 mg/m^2 will be administered as an IV infusion on Day 1 of each cycle, following administration of trastuzumab, for a total of six cycles or until investigator-assessed disease progression or unmanageable toxicity, whichever occurs first. Cisplatin may be administered beyond six cycles at the discretion of the investigator after careful risk-benefit assessment for individual patients.

- Capecitabine 1000 mg/m^2 will be administered orally twice daily, from the evening of Day 1 to the morning of Day 15 of each cycle, for a total of six cycles or until investigator-assessed disease progression or unmanageable toxicity, whichever occurs first. In the absence of disease progression or unmanageable toxicity, capecitabine may be administered beyond six cycles at the discretion of the investigator after careful risk-benefit assessment for individual patients.

The Pharmacy Manual contains specific instructions for the preparation of the pembrolizumab infusion fluid and administration of infusion solution.

5.2.3 Trial Blinding/Masking

This is an open-label trial; therefore, the investigator and subject will know the treatment administered.

5.3 Concomitant Medications/Vaccinations (allowed & prohibited)

Medications or vaccinations specifically prohibited in the exclusion criteria are not allowed during the ongoing trial. If there is a clinical indication for one of these or other medications or vaccinations specifically prohibited during the trial, discontinuation from trial therapy or vaccination may be required. The investigator should discuss any questions regarding this with the Merck Clinical team. The final decision on any supportive therapy or vaccination rests with the investigator and/or the subject's primary physician.

5.3.1 Acceptable Concomitant Medications

All treatments that the investigator considers necessary for a subject's welfare may be administered at the discretion of the investigator in keeping with the community standards of medical care. All concomitant medication will be recorded on the case report form (CRF) including all prescription, over-the-counter (OTC), herbal supplements, and IV medications and fluids. If changes occur during the trial period, documentation of drug dosage, frequency, route, and date may also be included on the CRF.

All concomitant medications received within 28 days before the first dose of trial treatment and 30 days after the last dose of trial treatment should be recorded. Concomitant medications administered after 30 days after the last dose of trial treatment should be recorded for SAEs and ECIs as defined in Section 7.2.

5.3.2 Prohibited Concomitant Medications

Subjects are prohibited from receiving the following therapies during the Screening and Treatment Phase (including retreatment for post-complete response relapse) of this trial:

- Antineoplastic systemic chemotherapy or biological therapy
- Immunotherapy not specified in this protocol
- Chemotherapy not specified in this protocol
- Investigational agents other than pembrolizumab
- Radiation therapy
    - Note: Radiation therapy to a symptomatic solitary lesion or to the brain may be allowed at the investigator's discretion.
- Live vaccines within 30 days prior to the first dose of trial treatment and while participating in the trial. Examples of live vaccines include, but are not limited to, the following: measles, mumps, rubella, varicella/zoster, yellow fever, rabies, BCG, and typhoid vaccine.
- Systemic glucocorticoids for any purpose other than to modulate symptoms from an event of clinical interest of suspected immunologic etiology. The use of physiologic doses of corticosteroids may be approved after consultation with the Sponsor (e.g., for control of acute asthma symptoms).
- Neither capecitabine nor 5-FU should be administered together with the antiviral drug sorivudine or its chemically related analogs, such as brivudine

- Subjects who, in the assessment by the investigator, require the use of any of the aforementioned treatments for clinical management should be removed from the trial. Subjects may receive other medications that the investigator deems to be medically necessary.

The Exclusion Criteria describes other medications which are prohibited in this trial. There are no prohibited therapies during the Post-Treatment Follow-up Phase.

5.4 Rescue Medications & Supportive Care

5.4.1 Supportive Care Guidelines for Pembrolizumab

Subjects should receive appropriate supportive care measures as deemed necessary by the treating investigator. Suggested supportive care measures for the management of adverse events with potential immunologic etiology are outlined below and in greater detail in the ECI guidance document. Where appropriate, these guidelines include the use of oral or intravenous treatment with corticosteroids as well as additional anti-inflammatory agents if symptoms do not improve with administration of corticosteroids. Note that several courses of steroid tapering may be necessary as symptoms may worsen when the steroid dose is decreased. For each disorder, attempts should be made to rule out other causes such as metastatic disease or bacterial or viral infection, which might require additional supportive care. The treatment guidelines are intended to be applied when the investigator determines the events to be related to pembrolizumab.

Note: if after the evaluation the event is determined not to be related, the investigator is instructed to follow the ECI reporting guidance but does not need to follow the treatment guidance (as outlined in the ECI guidance document). Refer to Section 5.2.1 for dose modification.

It may be necessary to perform conditional procedures such as bronchoscopy, endoscopy, or skin photography as part of evaluation of the event. Suggested conditional procedures, as appropriate, can be found in the ECI guidance document.

- **Pneumonitis**:
  - For **Grade 2 events**, treat with systemic corticosteroids. When symptoms improve to Grade 1 or less, steroid taper should be started and continued over no less than 4 weeks.
  - For **Grade 3-4 events**, immediately treat with intravenous steroids. Administer additional anti-inflammatory measures, as needed.
  - Add prophylactic antibiotics for opportunistic infections in the case of prolonged steroid administration.

- **Diarrhea/Colitis**:
  Subjects should be carefully monitored for signs and symptoms of enterocolitis (such as diarrhea, abdominal pain, blood or mucus in stool, with or without fever) and of bowel perforation (such as peritoneal signs and ileus).

  - All subjects who experience diarrhea/colitis should be advised to drink liberal quantities of clear fluids. If sufficient oral fluid intake is not feasible, fluid and electrolytes should be substituted via IV infusion. For Grade 2 or higher diarrhea, consider GI consultation and endoscopy to confirm or rule out colitis.
  - For **Grade 2 diarrhea/colitis** that persists greater than 3 days, administer oral corticosteroids.
  - For **Grade 3 or 4 diarrhea/colitis** that persists > 1 week, treat with intravenous steroids followed by high dose oral steroids.
  - When symptoms improve to Grade 1 or less, steroid taper should be started and continued over no less than 4 weeks.

- **Type 1 diabetes mellitus (if new onset, including diabetic ketoacidosis [DKA]) or ≥ Grade 3 Hyperglycemia, if associated with ketosis (ketonuria) or metabolic acidosis (DKA)**

  - For **T1DM** or **Grade 3-4** Hyperglycemia
    - Insulin replacement therapy is recommended for Type I diabetes mellitus and for Grade 3-4 hyperglycemia associated with metabolic acidosis or ketonuria.
    - Evaluate patients with serum glucose and a metabolic panel, urine ketones, glycosylated hemoglobin, and C-peptide.

- **Hypophysitis**:

  - For **Grade 2** events, treat with corticosteroids. When symptoms improve to Grade 1 or less, steroid taper should be started and continued over no less than 4 weeks. Replacement of appropriate hormones may be required as the steroid dose is tapered.
  - For **Grade 3-4** events, treat with an initial dose of IV corticosteroids followed by oral corticosteroids. When symptoms improve to Grade 1 or less, steroid

taper should be started and continued over no less than 4 weeks. Replacement of appropriate hormones may be required as the steroid dose is tapered.

- **Hyperthyroidism or Hypothyroidism**:
  Thyroid disorders can occur at any time during treatment. Monitor patients for changes in thyroid function (at the start of treatment, periodically during treatment, and as indicated based on clinical evaluation) and for clinical signs and symptoms of thyroid disorders.

  - **Grade 2** hyperthyroidism events (and **Grade 2-4** hypothyroidism):
    - In hyperthyroidism, non-selective beta-blockers (e.g. propranolol) are suggested as initial therapy.
    - In hypothyroidism, thyroid hormone replacement therapy, with levothyroxine or liothyroinine, is indicated per standard of care.
  - **Grade 3-4** hyperthyroidism
    - Treat with an initial dose of IV corticosteroid followed by oral corticosteroids. When symptoms improve to Grade 1 or less, steroid taper should be started and continued over no less than 4 weeks. Replacement of appropriate hormones may be required as the steroid dose is tapered.

- **Hepatic**:
  - For **Grade 2** events, monitor liver function tests more frequently until returned to baseline values (consider weekly).
    - Treat with IV or oral corticosteroids
  - For **Grade 3-4** events, treat with intravenous corticosteroids for 24 to 48 hours.
  - When symptoms improve to Grade 1 or less, a steroid taper should be started and continued over no less than 4 weeks.

- **Renal Failure or Nephritis**:
  - For **Grade 2** events, treat with corticosteroids.
  - For **Grade 3-4** events, treat with systemic corticosteroids.
  - When symptoms improve to Grade 1 or less, steroid taper should be started and continued over no less than 4 weeks.

- **Management of Infusion Reactions**: Signs and symptoms usually develop during or shortly after drug infusion and generally resolve completely within 24 hours of completion of infusion.

  Table 4 below shows treatment guidelines for subjects who experience an infusion reaction associated with administration of pembrolizumab (MK-3475).

Table 8. Infusion Reaction Treatment Guidelines

NCI CTCAE Grade	Treatment	Premedication at subsequent dosing
Grade 1<br>Mild reaction; infusion interruption not indicated; intervention not indicated	Increase monitoring of vital signs as medically indicated until the subject is deemed medically stable in the opinion of the investigator.	None
Grade 2<br>Requires infusion interruption but responds promptly to symptomatic treatment (e.g., antihistamines, NSAIDS, narcotics, IV fluids); prophylactic medications indicated for < =24 hrs	**Stop Infusion and monitor symptoms.**<br>Additional appropriate medical therapy may include but is not limited to:<br>IV fluids<br>Antihistamines<br>NSAIDS<br>Acetaminophen<br>Narcotics<br><br>Increase monitoring of vital signs as medically indicated until the subject is deemed medically stable in the opinion of the investigator.<br>If symptoms resolve within one hour of stopping drug infusion, the infusion may be restarted at 50% of the original infusion rate (e.g., from 100 mL/hr to 50 mL/hr). Otherwise dosing will be held until symptoms resolve and the subject should be	Subject may be premedicated 1.5h (± 30 minutes) prior to infusion of pembrolizumab (MK-3475) with:<br><br>Diphenhydramine 50 mg po (or equivalent dose of antihistamine).<br><br>Acetaminophen 500-1000 mg po (or equivalent dose of antipyretic).

NCI CTCAE Grade	Treatment	Premedication at subsequent dosing
	premedicated for the next scheduled dose.<br>**Subjects who develop Grade 2 toxicity despite adequate premedication should be permanently discontinued from further trial treatment administration.**	
<u>Grades 3 or 4</u><br><br>Grade 3:<br>Prolonged (i.e., not rapidly responsive to symptomatic medication and/or brief interruption of infusion); recurrence of symptoms following initial improvement; hospitalization indicated for other clinical sequelae (e.g., renal impairment, pulmonary infiltrates)<br><br>Grade 4:<br>Life-threatening; pressor or ventilatory support indicated	**Stop Infusion.**<br>Additional appropriate medical therapy may include but is not limited to:<br>IV fluids<br>Antihistamines<br>NSAIDS<br>Acetaminophen<br>Narcotics<br>Oxygen<br>Pressors<br>Corticosteroids<br>Epinephrine<br><br>Increase monitoring of vital signs as medically indicated until the subject is deemed medically stable in the opinion of the investigator.<br>Hospitalization may be indicated.<br>**Subject is permanently discontinued from further trial treatment administration.**	No subsequent dosing
Appropriate resuscitation equipment should be available in the room and a physician readily available during the period of drug administration.		

5.4.2 Supportive Care Guidelines for Trastuzumab

Administration of trastuzumab may result in infusion-associated symptoms such as nausea, pyrexia, diarrhea, chills, fatigue, and headache, or allergic reactions. The majority of hypersensitivity reactions was mild or moderate in severity and resolved upon treatment. Study treatment will be administered in a clinical treatment setting with emergency equipment and staff who are trained to monitor for and respond to medical emergencies. Any patient who experiences a Grade 4 allergic reaction, bronchospasm, or acute respiratory distress syndrome (ARDS) associated with trastuzumab administration will be withdrawn from study treatment on the basis of unacceptable toxicity. Patients will continue to be followed for clinical outcomes.

Patients who experience infusion-associated symptoms may be managed by slowing orinterrupting the trastuzumab infusion and by providing supportive care with oxygen and medications (e.g., beta-agonists, antihistamines, antipyretics, corticosteroids), as determined by the investigator to be clinically appropriate. Premedication with antipyretics, antihistamines, or corticosteroids may be administered before infusions of trastuzumab.

5.4.3 Supportive Care Guidelines for Cisplatin

Cisplatin 80 mg/m^2 will be administered as a 2-hour IV infusion on Day 1 of each cycle, for a total of six cycles or until investigator-assessed disease progression or unmanageable toxicity, whichever occurs first. Cisplatin will be administered after the trastuzumab infusion, once the 30- or 60-minute observation period has been completed. Investigators should administer pre-medication and ensure adequate hydration before and after infusion per institutional standards. For further details, see the local prescribing information for cisplatin.

5.5 Diet/Drug/Activity/Other Considerations

5.5.1 Diet and drug

Subjects should maintain a normal diet unless modifications are required to manage an AE such as diarrhea, nausea or vomiting.

5.5.2 Contraception

Pembrolizumab may have adverse effects on a fetus in utero. Furthermore, it is not known if pembrolizumab has transient adverse effects on the composition of sperm. Non-pregnant, non-breast-feeding women may be enrolled if they are willing to use 2 methods of birth control or are considered highly unlikely to conceive. Highly unlikely to conceive is defined as 1) surgically sterilized, or 2) postmenopausal (a woman who is ≥45 years of age and

has not had menses for greater than 1 year will be considered postmenopausal), or 3) not heterosexually active for the duration of the study. The two birth control methods can be either two barrier methods or a barrier method plus a hormonal method to prevent pregnancy. Subjects should start using birth control from study Visit 1 throughout the study period up to 120 days after the last dose of study therapy.

The following are considered adequate barrier methods of contraception: diaphragm, condom (by the partner), copper intrauterine device, sponge, or spermicide. Appropriate hormonal contraceptives will include any registered and marketed contraceptive agent that contains an estrogen and/or a progestational agent (including oral, subcutaneous, intrauterine, or intramuscular agents).

Subjects should be informed that taking the study medication may involve unknown risks to the fetus (unborn baby) if pregnancy were to occur during the study. In order to participate in the study they must adhere to the contraception requirement (described above) for the duration of the study and during the follow-up period defined in section 7.2.2-Reporting of Pregnancy and Lactation to the Sponsor and to Merck. If there is any question that a subject will not reliably comply with the requirements for contraception, that subject should not be entered into the study.

5.5.3 Use in Pregnancy

If a subject inadvertently becomes pregnant while on treatment with pembrolizumab, the subject will immediately be removed from the study. The site will contact the subject at least monthly and document the subject's status until the pregnancy has been completed or terminated. The outcome of the pregnancy will be reported to the Sponsor and to Merck without delay and within 24 hours to the Sponsor and within 2 working days to Merck if the outcome is a serious adverse experience (e.g., death, abortion, congenital anomaly, or other disabling or life-threatening complication to the mother or newborn).

The study investigator will make every effort to obtain permission to follow the outcome of the pregnancy and report the condition of the fetus or newborn to the Sponsor. If a male subject impregnates his female partner the study personnel at the site must be informed immediately and the pregnancy reported to the Sponsor and to Merck and followed as described above and in Section 7.2.2.

5.5.4 Use in Nursing Women

It is unknown whether pembrolizumab is excreted in human milk. Since many drugs are excreted in human milk, and because of the potential for serious adverse reactions in the nursing infant, subjects who are breast-feeding are not eligible for enrollment.

5.6 Subject Withdrawal/Discontinuation Criteria

Subjects may withdraw consent at any time for any reason or be dropped from the trial at the discretion of the investigator should any untoward effect occur. In addition, a subject may be withdrawn by the investigator or the Sponsor if enrollment into the trial is inappropriate, the trial plan is violated, or for administrative and/or other safety reasons. Specific details regarding discontinuation or withdrawal are provided in Section 7.1.4 – Other Procedures.

A subject must be discontinued from the trial for any of the following reasons:

- The subject or legal representative (such as a parent or legal guardian) withdraws consent.
- Confirmed radiographic disease progression

  Note: A subject may be granted an exception to continue on treatment with confirmed radiographic progression if clinically stable or clinically improved, please see Section 7.1.2.7.1

- Unacceptable adverse experiences as described in Section 5.2.1.2
- Intercurrent illness that prevents further administration of treatment
- Investigator's decision to withdraw the subject
- The subject has a confirmed positive serum pregnancy test
- Noncompliance with trial treatment or procedure requirements
- The subject is lost to follow-up
- Completed 35 administrations of pembrolizumab and trastuzumab (additional administration may be considered at the discretion of the investigator in consultation with the patient, optional)
- Administrative reasons

The End of Treatment and Follow-up visit procedures are listed in Section 6 (Protocol Flow Chart). After the end of treatment, each subject will be followed for 30 days for adverse event monitoring (serious adverse events will be collected for 90 days after the end of treatment as described). Subjects who discontinue for reasons other than progressive disease will have post-treatment follow-up for disease status until disease progression, initiating a non-study cancer

treatment, withdrawing consent or becoming lost to follow-up. After documented disease progression each subject will be followed by telephone for overall survival until death, withdrawal of consent, or the end of the study, whichever occurs first.

5.6.1 Discontinuation of Study Therapy after CR

Discontinuation of treatment may be considered for subjects who have attained a confirmed CR that have been treated for at least 24 weeks based on the investigator's judgement.

5.7 Subject Replacement Strategy

A subject who discontinues from the trial will not be replaced.

5.8 Clinical Criteria for Early Trial Termination

Early trial termination will be the result of the criteria specified below:

1) Quality or quantity of data recording is inaccurate or incomplete

2) Poor adherence to protocol and regulatory requirements

3) Incidence or severity of adverse drug reaction in this or other studies indicates a potential health hazard to subjects

4) Plans to modify or discontinue the development of the study drug

In the event of Merck decision to no longer supply study drug, ample notification will be provided so that appropriate adjustments to subject treatment can be made.

6.0 TRIAL FLOW CHART

6.1 Study Flow Chart

Trial Period:	Screening Phase	Treatment Cycles[a]									Post-Treatment	
Treatment Cycle/Title:	Study Screening	Combination						Maintenance (To be repeated beyond 6 cycles)			Safety Follow-up Follow Up Visits	Survival Follow-Up
		1	2	3	4	5	6	7	8	9		
Scheduling Window (Days)[b]:	-28 to -1	±3	± 3	± 3	± 3	± 3	± 3	± 3	± 3	± 3	~30 days post discon	Every 16 weeks (± 7)
**Administrative Procedures**												
Informed Consent	X											
Inclusion/Exclusion Criteria	X											
Demographics and Medical History	X											
Prior and Concomitant Medication Review	X	X	X	X	X	X	X	X	X	X	X	
Trial Treatment Administration		X	X	X	X	X	X	X	X	X		
Post-study anticancer therapy status											X	X

**Trial Period:**	**Screening Phase**	**Treatment Cycles[a]**									**Post-Treatment**	
Treatment Cycle/Title:	Study Screening	**Combination**						**Maintenance (To be repeated beyond 6 cycles)**			Safety Follow-up Follow Up Visits	Survival Follow-Up
		1	2	3	4	5	6	7	8	9		
Scheduling Window (Days)[b]:	-28 to -1	±3	± 3	± 3	± 3	± 3	± 3	± 3	± 3	± 3	~30 days post discon	Every 16 weeks (± 7)
**Clinical Procedure/Assessments**												
Survival Status												X
Review Adverse Events	X										X	
Full Physical Examination	X											
Directed Physical Examination		X	X	X	X	X	X	X	X	X	X	
Vital Signs and Weight	X	X	X	X	X	X	X	X	X	X	X	
ECOG Performance Status	X	X	X	X	X	X	X	X	X	X	X	
**Laboratory Procedures [d]**												
Pregnancy Test – Urine or Serum □ -HCG[c]	X											
PT/INR and aPTT	X											
CBC with Differential	X		X	X	X	X	X	X	X	X	X	

Trial Period:	Screening Phase	Treatment Cycles[a]									Post-Treatment	
Treatment Cycle/Title:	Study Screening	Combination						Maintenance (To be repeated beyond 6 cycles)			Safety Follow-up Follow Up Visits	Survival Follow-Up
		1	2	3	4	5	6	7	8	9		
Scheduling Window (Days)[b]:	-28 to -1	±3	± 3	± 3	± 3	± 3	± 3	± 3	± 3	± 3	~30 days post discon	Every 16 weeks (± 7)
Comprehensive Serum Chemistry Panel	X		X	X	X	X	X	X	X	X	X	
Urinalysis	X											
T3, FT4 and TSH	X		X		X		X		X			
EKG	X				X				X			
Echo	X				X				X			
Chest X-ray	X	If needed										
**Efficacy measurement**												
Tumor Imaging[f]	X			X		X		X		X	X[h]	
**Tissue/Blood biomarker collection [g]**												
Blood for lymphocyte analysis	X			X			X			X	X	
Blood for NK cell activity	X		X		X					X	X	

**Trial Period:**	**Screening Phase**	**Treatment Cycles**[a]									**Post-Treatment**	
Treatment Cycle/Title:	Study Screening	**Combination**						**Maintenance (To be repeated beyond 6 cycles)**			Safety Follow-up Follow Up Visits	Survival Follow-Up
		1	2	3	4	5	6	7	8	9		
Scheduling Window (Days)[b]:	-28 to -1	±3	± 3	± 3	± 3	± 3	± 3	± 3	± 3	± 3	~30 days post discon	Every 16 weeks (± 7)
Tissue Collection	X[i]			(X)						(X)	(X)	

a. Unless otherwise specified, assessments/procedures are to be performed on Day 1 and prior to the first dose of treatment for each cycle.
b. Unless otherwise specified, the window for each visit is ± 3 days.
c. For women of reproductive potential, a negative pregnancy test should be confirmed within 72 hours prior to first dose of trial treatment to be eligible for the trial.
d. Laboratory tests for screening are to be performed within 14 days prior to the first dose of trial treatment. After Cycle 1, lab samples can be collected up to ±3 days prior to the scheduled time point.
f. Baseline tumor imaging will be performed within 28 days prior to the first dose of trial treatment Scans performed as part of routine clinical management are acceptable for use as the baseline scan if they are of diagnostic quality and performed within the allotted screening window (± 7 days). After 1 year, imaging time point will occur every 9 weeks (± 7 days). Imaging timing should follow calendar days and should not be adjusted due to dose interruptions. The same imaging technique, acquisition, and processing parameters should be used in a subject throughout the trial.
g. Informed consent for the optional future biomedical research samples must be obtained before the DNA sample is collected.
h. In subjects who discontinue study therapy without confirmed disease progression, a radiologic evaluation should be performed within 6 weeks of treatment discontinuation.
Every effort should be made to continue monitoring their disease status by radiologic imaging every 12 weeks (± 7 days) until (1) the start of new anti-cancer treatment, (2) diseaseprogression, (3) death, or (4) the end of the study, whichever occurs first. For subjects who discontinue study due to progression, then a scan is not required.
i. Baseline fresh tumor biopsy will be obtained prior to the initiation of study for all cases. On-treatment (cycle 3 and 9) and post-treatment tissue collection will be done for the available cases (X).

7.0 TRIAL PROCEDURES

7.1 Trial Procedures

The Trial Flow Chart - Section 6.0 summarizes the trial procedures to be performed at each visit. Individual trial procedures are described in detail below. It may be necessary to perform these procedures at unscheduled time points if deemed clinically necessary by the investigator.

7.1.1 Administrative Procedures

7.1.1.1 Informed Consent

The Investigator must obtain documented consent from each potential subject prior to participating in a clinical trial.

7.1.1.1.1 General Informed Consent

Consent must be documented by the subject's dated signature or by the subject's legally acceptable representative's dated signature on a consent form along with the dated signature of the person conducting the consent discussion. A copy of the signed and dated consent form should be given to the subject before participation in the trial.

The initial informed consent form, any subsequent revised written informed consent form and any written information provided to the subject must receive the IRB/ERC's approval/favorable opinion in advance of use. The subject or his/her legally acceptable representative should be informed in a timely manner if new information becomes available that may be relevant to the subject's willingness to continue participation in the trial. The communication of this information will be provided and documented via a revised consent form or addendum to the original consent form that captures the subject's dated signature or by the subject's legally acceptable representative's dated signature.

Specifics about a trial and the trial population will be added to the consent form template at the protocol level. The informed consent will adhere to IRB/ERC requirements, applicable laws and regulations and Sponsor requirements.

7.1.1.2 Inclusion/Exclusion Criteria

All inclusion and exclusion criteria will be reviewed by the investigator or qualified designee to ensure that the subject qualifies for the trial.

7.1.1.3 Medical History

A medical history will be obtained by the investigator or qualified designee. Medical history will include all active conditions, and any condition diagnosed within the prior 10 years that are considered to be clinically significant by the Investigator. Details regarding the disease for which the subject has enrolled in this study will be recorded separately and not listed as medical history.

7.1.1.4 Prior and Concomitant Medications Review

7.1.1.4.1 Prior Medications

The investigator or qualified designee will review prior medication use, including any protocol-specified washout requirement, and record prior medication taken by the subject within 28 days before starting the trial. Treatment for the disease for which the subject has enrolled in this study will be recorded separately and not listed as a prior medication.

7.1.1.4.2 Concomitant Medications

The investigator or qualified designee will record medication, if any, taken by the subject during the trial. All medications related to reportable SAEs and ECIs should be recorded as defined in Section 7.2.

7.1.1.5 Disease Details and Treatments

7.1.1.5.1 Disease Details

The investigator or qualified designee will obtain prior and current details regarding disease status.

7.1.1.5.2 Prior Treatment Details

The investigator or qualified designee will review all prior cancer treatments including systemic treatments, radiation and surgeries.

7.1.1.5.3 Subsequent Anti-Cancer Therapy Status

The investigator or qualified designee will review all new anti-neoplastic therapy initiated after the last dose of trial treatment. If a subject initiates a new anti-cancer therapy within 30 days after the last dose of trial treatment, Safety Follow-up visit must occur before the first dose of the new therapy. Once new anti-cancer therapy has been initiated the subject will move into survival follow-up.

7.1.1.6 Assignment of Screening Number

All consented subjects will be given a unique screening number that will be used to identify the subject for all procedures that occur prior to randomization or allocation. Each subject will be assigned only one screening number. Screening numbers must not be re-used for different subjects. Any subject who is screened multiple times will retain the original screening number assigned at the initial screening visit.

7.1.1.7 Trial Compliance (Medication/Diet/Activity/Other)

Interruptions from the protocol specified treatment plan for greater than 6 weeks require consultation between the investigators and written documentation of the collaborative decision on subject management.

7.1.2 Clinical Procedures/Assessments

7.1.2.1 Adverse Event (AE) Monitoring

The investigator or qualified designee will assess each subject to evaluate for potential new or worsening AEs as specified in the Trial Flow Chart and more frequently if clinically indicated. Adverse experiences will be graded and recorded throughout the study and during the follow-up period according to NCI CTCAE Version 4.03 (see Section 11.2). Toxicities will be characterized in terms regarding seriousness, causality, toxicity grading, and action taken with regard to trial treatment.

For subjects receiving treatment with pembrolizumab all AEs of unknown etiology associated with pembrolizumab exposure should be evaluated to determine if it is possibly an event of clinical interest (ECI) of a potentially immunologic etiology (termed immune-related adverse events, or irAEs); see the separate ECI guidance document in Appendix 4 regarding the identification, evaluation and management of potential irAEs.

7.1.2.2 Full Physical Exam

The investigator or qualified designee will perform a complete physical exam during the screening period. Clinically significant abnormal findings should be recorded as medical history. A full physical exam should be performed during screening,

7.1.2.3 Directed Physical Exam

For cycles that do not require a full physical exam per the Trial Flow Chart, the investigator or qualified designee will perform a directed physical exam as clinically indicated prior to trial treatment administration.

7.1.2.4 Vital Signs

The investigator or qualified designee will take vital signs at screening, prior to the administration of each dose of trial treatment and at treatment discontinuation as specified in the Trial Flow Chart (Section 6.0). Vital signs should include temperature, pulse, respiratory rate, weight and blood pressure. Height will be measured at screening only.

7.1.2.5 Eastern Cooperative Oncology Group (ECOG) Performance Scale

The investigator or qualified designee will assess ECOG status at screening, prior to the administration of each dose of trial treatment and discontinuation of trial treatment as specified in the Trial Flow Chart.

7.1.2.6 Tumor Imaging and Assessment of Disease

Tumor imaging may be performed by computed tomography (CT) (preferred) or magnetic resonance imaging (MRI), but the same imaging technique, acquisition, and processing parameters should be used in a subject throughout the trial. In determining whether or not the tumor burden has increased or decreased, investigators should consider all target lesions as well as non-target lesions

- **Baseline Tumor Imaging**

Baseline tumor imaging must be performed within 28 days prior to the first dose of treatment. Scans performed as part of routine clinical management are acceptable for use as the baseline scan if they are of diagnostic quality.

- **Tumor Imaging During Trial**

On-study imaging should be performed every 6 weeks (42 days ± 7 days), or more frequently if clinically indicated. Imaging should follow calendar days and not be delayed for any dose interruptions that may occur. Per RECIST 1.1, response should be confirmed by a repeat radiographic assessment not less than 4 weeks from the date the response was first documented. The scan for confirmation of response may be performed at the earliest 4 weeks after the first indication of response, or at the next scheduled scan (6 weeks later), whichever is clinically indicated.

Imaging should continue to be performed until disease progression, the start of new anticancer treatment, withdrawal of consent, death, or the end of the study, whichever occurs first. Disease progression may be confirmed at least 4 weeks after the first scan indicating progressive disease in clinically stable subjects. Subjects who have unconfirmed disease progression may continue on treatment until progression is confirmed (or possible even later)

7.1.2.7 Immune-related RECIST (irRECIST)

Following PD by RECIST, sites will assess tumor response and progression per immune - related RECIST (irRECIST) for subjects receiving pembrolizumab as this data will be collected in the clinical database. irRECIST is RECIST 1.1 adapted for use with immunotherapies as described in the Procedure Manual and irRECIST Tip Sheet.

If initial imaging shows progressive disease (PD), tumor assessment may be repeated by the site at least 4 weeks later in order to confirm PD with the option of continuing treatment until this scan is obtained for clinically stable subjects (see Table 9). Clinically stable is defined by the following criteria:

	Clinically Stable		Clinically Unstable	
	**Imaging**	**Treatment**	**Imaging**	**Treatment**
1st radiologic evidence of PD	Repeat imaging at ≥ 4 weeks to confirm PD	May continue study treatment at the Investigator's discretion while awaiting confirmatory scan	Repeat imaging at ≥ 4 weeks to confirm PD if possible	Discontinue treatment
Repeat scan confirms PD	No additional imaging required	Discontinue treatment (exception is possible upon consultation with Sponsor).	No additional imaging required	N/A
Repeat scan shows SD, PR or CR	Continue regularly scheduled imaging assessments every 6 weeks	Continue study treatment at the Investigator's discretion	Continue regularly scheduled imaging assessments every 6 weeks	May restart study treatment if condition has improved and/or clinically stable per Investigator's discretion

Absence of signs and symptoms indicating disease progression

- No decline in ECOG performance status
- Absence of rapid progression of disease
- Absence of progressive tumor at critical anatomical sites (e.g., cord compression) requiring urgent alternative medical intervention

In determining whether or not the tumor burden has increased or decreased, investigators should consider all target lesions as well as non-target lesions (please refer to the irRECIST Tip Sheet). Subjects that are deemed clinically unstable are not required to have repeat imaging

for confirmation. If radiologic progression is confirmed, it is recommended that the subject be discontinued from trial treatment unless, in the investigator's opinion, the subject is deriving benefit from treatment. Clinically stable subjects as defined above may continue to receive trial therapy after discussion with the Sponsor. If a subject has unconfirmed progression of disease and is clinically stable, it is at the discretion of the investigator to continue treating the subject with the assigned treatment per protocol until progression of disease is confirmed at least 28 days from the date of the scan suggesting progression of disease. If progression is not confirmed on the subsequent scan, the subject should continue to receive study therapy and radiographic scans obtained to monitor for disease status every 6 weeks (42 ± 7 days).

7.1.2.8 Tumor Tissue Collection and Correlative Studies Blood Sampling

To demonstrate mechanism of response of pembrolizumab and trastuzumab combination, blood and tumor tissue analysis from newly-obtained tumor will be tested. Detailed information presented in Section 4.2.4.3 Biomarker Research and outlined in the Study Flow Chart-Section

7.1.3 Laboratory Procedures/Assessments

Details regarding specific laboratory procedures/assessments to be performed in this trial are provided below Laboratory Safety Evaluations (Hematology, Chemistry and Urinalysis) Laboratory tests for hematology, chemistry, urinalysis, and others are specified in Table 8.

Laboratory tests for screening or entry into the Second Course Phase should be performed within 14 days prior to the first dose of treatment. After Cycle 1, pre-dose laboratory procedures can be conducted up to ±3 days prior to dosing. Results must be reviewed by the investigator or qualified designee and found to be acceptable prior to each dose of trial treatment.

Table 10. Laboratory Tests

Hematology	Chemistry	Urinalysis	Other
Hematocrit	Albumin	Blood	Serum β-human chorionic gonadotropin†
Hemoglobin	Alkaline phosphatase	Glucose	(β-hCG)†
Platelet count	Alanine aminotransferase (ALT)	Protein	PT (INR)
WBC (total and differential)	Aspartate aminotransferase (AST)	Specific gravity	aPTT
Red Blood Cell Count	Lactate dehydrogenase (LDH)	Microscopic exam (If abnormal results are noted)	Total thriiodothyronine (T3)
Absolute Neutrophil Count	Uric Acid		Free tyroxine (T4)
Absolute Lymphocyte Count	Calcium	Urine pregnancy test †	Thyroid stimulating hormone (TSH)
	Chloride		
	Glucose		
	Phosphorus		Blood for correlative studies
	Potassium		
	Sodium		
	Magnesium		
	Total Bilirubin		
	Direct Bilirubin *(If total bilirubin is elevated above the upper limit of normal)*		
	Total protein		

Hematology	Chemistry	Urinalysis	Other
	Blood Urea Nitrogen		
† Perform on women of childbearing potential only. If urine pregnancy results cannot be confirmed as negative, a serum pregnancy test will be required.			

7.1.3.1 Tissue/blood Biomarker Collection

7.1.3.1.1 Blood Collection for lymphocyte subset anaysis

Blood (8 mL) will be drawn into labeled 2 EDTA tubes (4 mL each) and prepared in Yonsei Cancer Center.

- Plasma and PBMC: 1 sample will be centrifuged within 30 minutes of collection at 0 to 5°C at 3000 rpm and the plasma withdrawn. The plasma and PBMC will be transferred to BICELL and stored at -80°C
- Whole blood: 1 sample will be placed in a freezer and stored at -80°C

7.1.3.1.2 NK cell activity

- Blood (4 mL) will be drawn into labeled 1 EDTA tube and prepared in Department of Microbiology, Yonsei University College of Medicine.

7.1.3.1.3 Tissue collection

Baseline tumor biopsy will be obtained from stomach and/or the gastroesophageal junction tumor by an endoscopic procedure prior to the initiation of study treatment (core needle biopsy procedure for acquisition of metastatic site is also feasible as baseline tissue) for all the cases. On-treatment (cycle 3 and 9) and post-treatment tissue collection will be done for the available cases.

7.1.4 Other Procedures

7.1.4.1 Withdrawal/Discontinuation

When a subject discontinues/withdraws prior to trial completion, all applicable activities scheduled for the final trial visit should be performed at the time of discontinuation. Any adverse events which are present at the time of discontinuation/withdrawal should be followed in accordance with the safety requirements.

7.1.5 Visit Requirements

Visit requirements are outlined in Section 6.0 - Trial Flow Chart. Specific procedure-related details are provided above in Section 7.1 - Trial Procedures.

7.1.5.1 Screening

7.1.5.1.1 Screening Period

Screening procedures are to be completed within 28 days prior to the first dose of trial treatment except for the following:

- Laboratory tests and ECOG performance status are to be performed within 14 days prior to the first dose of trial treatment.
- For women of reproductive potential, a urine pregnancy test will be performed within 72 hours prior to the first dose of trial treatment.

7.1.5.2 Treatment Period

Visit requirements are outlined in Section 6.0 – Trial Flow Chart. Specific procedure-related details are provided above in Section 7.1 – Trial Procedures

7.1.5.3 Post-Treatment Visits

7.1.5.3.1 Safety Follow-Up Visit

The mandatory Safety Follow-Up Visit should be conducted within 30 days after the last dose of trial treatment or before the initiation of a new anti-cancer treatment, whichever comes first. All AEs that occur prior to the Safety Follow-Up Visit should be recorded. Subjects with an AE of Grade > 1 will be followed until the resolution of the AE to Grade 0-1 or until the beginning of a new anti-neoplastic therapy, whichever occurs first. SAEs that occur within 30 days of the end of treatment or before initiation of a new anti-cancer treatment should also be followed and recorded.

7.1.5.3.2 Survival Follow-up

Once a subject experiences confirmed disease progression or starts a new anti-cancer therapy, the subject moves into the survival follow-up phase and should be contacted by telephone every 16 weeks to assess for survival status until death, withdrawal of consent, or the end of the study, whichever occurs first.

7.1.5.4 Assessing and Recording Adverse Events

An adverse event is defined as any untoward medical occurrence in a patient or clinical investigation subject administered a pharmaceutical product and which does not necessarily have to have a causal relationship with this treatment. An adverse event can therefore be any unfavorable and unintended sign (including an abnormal laboratory finding, for example), symptom, or disease temporally associated with the use of a medicinal product or protocol-specified procedure, whether or not considered related to the medicinal product or protocol-specified procedure. Any worsening (i.e., any clinically significant adverse change in frequency and/or intensity) of a preexisting condition that is temporally associated with the use of the Merck's product, is also an adverse event.

Changes resulting from normal growth and development that do not vary significantly in frequency or severity from expected levels are not to be considered adverse events. Examples of this may include, but are not limited to, teething, typical crying in infants and children and onset of menses or menopause occurring at a physiologically appropriate time.

Merck product includes any pharmaceutical product, biological product, device, diagnostic agent or protocol-specified procedure, whether investigational (including placebo or active comparator medication) or marketed, manufactured by, licensed by, provided by or distributed by Merck for human use.

Adverse events may occur during the course of the use of Merck product in clinical trials or within the follow-up period specified by the protocol, or prescribed in clinical practice, from overdose (whether accidental or intentional), from abuse and from withdrawal.

Adverse events may also occur in screened subjects during any pre-allocation baseline period as a result of a protocol-specified intervention, including washout or discontinuation of usual therapy, diet, placebo treatment or a procedure.

Progression of the cancer under study is not considered an adverse event unless it is considered to be drug related by the investigator.

All adverse events will be recorded from the time the consent form is signed through 30 days following cessation of treatment and at each examination on the Adverse Event case report forms/worksheets. The reporting timeframe for adverse events meeting any serious criteria is described in section 7.2.3.1.

7.1.5.5 Definition of an Overdose for This Protocol and Reporting of Overdose to the Sponsor and to Merck

For purposes of this trial, an overdose of pembrolizumab will be defined as any dose of 1,000 mg or greater (≥5 times the indicated dose). No specific information is available on the treatment of overdose of pembrolizumab. Appropriate supportive treatment should be provided if clinically indicated. In the event of overdose, the subject should be observed closely for signs of toxicity. Appropriate supportive treatment should be provided if clinically indicated.

If an adverse event(s) is associated with ("results from") the overdose of a Merck product, the adverse event(s) is reported as a serious adverse event, even if no other seriousness criteria are met.

If a dose of Merck's product meeting the protocol definition of overdose is taken without any associated clinical symptoms or abnormal laboratory results, the overdose is reported as a non-serious Event of Clinical Interest (ECI), using the terminology "accidental or intentional overdose without adverse effect."

All reports of overdose with and without an adverse event must be reported within 24 hours to the principle investigator (PI) and IRB and within 2 working days to Merck Global Safety (Attn: Worldwide Product Safety; FAX 215 993-1220) and CELLTRION.

7.1.6 Reporting of Pregnancy and Lactation to the Sponsor and to Merck

Although pregnancy and lactation are not considered adverse events, it is the responsibility of investigators or their designees to report any pregnancy or lactation in a subject (spontaneously reported to them), including the pregnancy of a male subject's female partner that occurs during the trial or within 120 days of completing the trial completing the trial, or 30 days following cessation of treatment if the subject initiates new anticancer therapy, whichever is earlier. All subjects and female partners of male subjects who become pregnant must be followed to the completion/termination of the pregnancy. Pregnancy outcomes of spontaneous abortion, missed abortion, benign hydatidiform mole, blighted ovum, fetal death, intrauterine death, miscarriage and stillbirth must be reported as serious events (Important Medical Events). If the pregnancy continues to term, the outcome (health of infant) must also be reported.

Such events must be reported within 24 hours to the PI (and IRB) and within 2 working days to Merck Global Safety (Attn: Worldwide Product Safety; FAX 215 993-1220) and CELLTRION.

7.1.7 Immediate Reporting of Adverse Events to the Sponsor and to Merck

7.1.7.1 Serious Adverse Events

A serious adverse event is any adverse event occurring at any dose or during any use of Merck's product that:

- Results in death;
- Is life threatening;
- Results in persistent or significant disability/incapacity;
- Results in or prolongs an existing inpatient hospitalization;
- Is a congenital anomaly/birth defect;
- Is a new cancer (that is not a condition of the study);
- Is associated with an overdose;
- Is another important medical event

Refer to Table 9 for additional details regarding each of the above criteria.

Any serious adverse event, or follow up to a serious adverse event, including death due to any cause other than progression of the cancer under study that occurs to any subject from the time the consent is signed through 30 days following cessation of treatment, or the initiation of new anti-cancer therapy, whichever is earlier, whether or not related to Merck product, must be reported within 24 hours to the PI (and IRB) and within 2 working days to Merck Global Safety and CELLTRION.

Non-serious Events of Clinical Interest will be forwarded to Merck Global Safety and CELLTRION and will be handled in the same manner as SAEs.

Additionally, any serious adverse event, considered by an investigator who is a qualified physician to be related to Merck product that is brought to the attention of the investigator at any time outside of the time period specified in the previous paragraph also must be reported immediately to the Sponsor and to Merck.

SAE reports and any other relevant safety information are to be forwarded to the Merck Global Safety facsimile number: +1-215-993-1220

A copy of all 15 Day Reports and Annual Progress Reports is submitted as required by FDA, European Union (EU), Pharmaceutical and Medical Devices agency (PMDA) or other local regulators. Investigators will cross reference this submission according to local regulations to the Merck Investigational Compound Number (IND, CSA, etc.) at the time of submission. Additionally investigators will submit a copy of these reports to Merck & Co.,

Inc. (Attn: Worldwide Product Safety; FAX 215 993-1220) at the time of submission to FDA. All subjects with serious adverse events must be followed up for outcome.

7.1.7.2 Events of Clinical Interest

Selected non-serious and serious adverse events are also known as Events of Clinical Interest (ECI) and must be recorded as such on the Adverse Event case report forms/worksheets and reported within 24 hours to the PI and within 2 working days to Merck Global Safety. (Attn: Worldwide Product Safety; FAX 215 993-1220). Events of clinical interest for this trial include:

1) An overdose of Merck product, as defined in Section 7.2.1 - Definition of an Overdose for This Protocol and Reporting of Overdose to the Sponsor, that is not associated with clinical symptoms or abnormal laboratory results.

2) An elevated AST or ALT lab value that is greater than or equal to 3X the upper limit of normal and an elevated total bilirubin lab value that is greater than or equal to 2X the upper limit of normal and, at the same time, an alkaline phosphatase lab value that is less than 2X the upper limit of normal, as determined by way of protocol-specified laboratory testing or unscheduled laboratory testing.*

> *Note: These criteria are based upon available regulatory guidance documents. The purpose of the criteria is to specify a threshold of abnormal hepatic tests that may require an additional evaluation for an underlying etiology. The trial site guidance for assessment and follow up of these criteria can be found in the Investigator Trial File Binder (or equivalent).

3) Additional adverse events:

A separate guidance document has been provided entitled “Event of Clinical Interest Guidance Document” (previously entitled, “Event of Clinical Interest and Immune-Related Adverse Event Guidance Document”). This document can be found in Appendix 4 and provides guidance regarding identification, evaluation and management of ECIs and irAEs.

ECIs (both non-serious and serious adverse events) identified in this guidance document from the date of first dose through 90 days following cessation of treatment, or 30 days after the initiation of a new anticancer therapy, whichever is earlier, need to be reported within 24 hours to the PI and within 2 working days to Merck Global Safety. (Attn: Worldwide Product Safety; FAX 215 993-1220), regardless of attribution to study treatment, consistent with standard SAE reporting guidelines.

Subjects should be assessed for possible ECIs prior to each dose. Lab results should be evaluated and subjects should be asked for signs and symptoms suggestive of an immune-related event. Subjects who develop an ECI thought to be immune-related should have additional testing to rule out other etiologic causes. If lab results or symptoms indicate a possible immune-related ECI, then additional testing should be performed to rule out other etiologic causes. If no other cause is found, then it is assumed to be immune-related.

7.1.8 Evaluating Adverse Events

An investigator who is a qualified physician will evaluate all adverse events according to the NCI Common Terminology for Adverse Events (CTCAE), version 4.03. Any adverse event which changes CTCAE grade over the course of a given episode will have each change of grade recorded on the adverse event case report forms/worksheets. All adverse events regardless of CTCAE grade must also be evaluated for seriousness.

Table 11. Evaluating Adverse Events

An investigator who is a qualified physician, will evaluate all adverse events as to:

**V4.03 CTCAE Grading**	**Grade 1**	**Mild; asymptomatic or mid symptoms; clinical or diagnostic observations only; intervention not indicated.**
	**Grade 2**	**Moderate; minimal, local or noninvasive intervention indicated; limiting age-appropriate instrumental ADL.**
	**Grade 3**	**Severe or medically significant but not immediately life-threatening; hospitalization or prolongation or hospitalization indicated; disabling; limiting self-care ADL.**
	**Grade 4**	**Life threatening consequences; urgent intervention indicated.**
	**Grade 5**	**Death related to AE**
**Seriousness**	A serious adverse event is any adverse event occurring at any dose or during any use of Merck product that:	
	†**Results in death**; or	
	†**Is life threatening; or** places the subject, in the view of the investigator, at immediate risk of death from the event as it occurred (Note: This does not include an adverse event that, had it occurred in a more severe form, might have caused death.); or	
	†**Results in a persistent or significant disability/incapacity** (substantial disruption of one's ability to conduct normal life functions); or	
	†**Results in or prolongs an existing inpatient hospitalization** (hospitalization is defined as an inpatient admission, regardless of length of stay, even if the hospitalization is a precautionary measure for continued observation. (Note: Hospitalization [including hospitalization for an elective procedure] for a preexisting condition which has not worsened does not constitute a serious adverse event.); or	
	†**Is a congenital anomaly/birth defect** (in offspring of subject taking the product regardless of time to diagnosis);or	
	**Is a** new **cancer;** (that is not a condition of the study) **or**	

<table>
<tr><td></td><td colspan="2">Is an overdose (whether accidental or intentional). Any adverse event associated with an overdose is considered a serious adverse event. An overdose that is not associated with an adverse event is considered a non-serious event of clinical interest and must be reported within 24 hours.</td></tr>
<tr><td></td><td colspan="2">Other important medical events that may not result in death, not be life threatening, or not require hospitalization may be considered a serious adverse event when, based upon appropriate medical judgment, the event may jeopardize the subject and may require medical or surgical intervention to prevent one of the outcomes listed previously (designated above by a †).</td></tr>
<tr><td>Duration</td><td colspan="2">Record the start and stop dates of the adverse event. If less than 1 day, indicate the appropriate length of time and units</td></tr>
<tr><td>Action taken</td><td colspan="2">Did the adverse event cause the Merck product to be discontinued?</td></tr>
<tr><td>Relationship to test drug</td><td colspan="2">Did the Merck product cause the adverse event? The determination of the likelihood that the Merck product caused the adverse event will be provided by an investigator who is a qualified physician. The investigator's signed/dated initials on the source document or worksheet that supports the causality noted on the AE form, ensures that a medically qualified assessment of causality was done. This initialed document must be retained for the required regulatory time frame. The criteria below are intended as reference guidelines to assist the investigator in assessing the likelihood of a relationship between the test drug and the adverse event based upon the available information.
The following components are to be used to assess the relationship between the Merck product and the AE; the greater the correlation with the components and their respective elements (in number and/or intensity), the more likely the Merck product caused the adverse event (AE):</td></tr>
<tr><td></td><td>Exposure</td><td>Is there evidence that the subject was actually exposed to the Merck product such as: reliable history, acceptable compliance assessment (pill count, diary, etc.), expected pharmacologic effect, or measurement of drug/metabolite in bodily specimen?</td></tr>
<tr><td></td><td>Time Course</td><td>Did the AE follow in a reasonable temporal sequence from administration of the Merck product? Is the time of onset of the AE compatible with a drug-induced effect (applies to trials with investigational medicinal product)?</td></tr>
<tr><td></td><td>Likely Cause</td><td>Is the AE not reasonably explained by another etiology such as underlying disease, other drug(s)/vaccine(s), or other host or environmental factors</td></tr>
</table>

**Relationship to Merck product (continued)**	**The following components are to be used to assess the relationship between the test drug and the AE: (continued)**	
	**Dechallenge**	Was the Merck product discontinued or dose/exposure/frequency reduced?<br>If yes, did the AE resolve or improve?<br>If yes, this is a positive dechallenge. If no, this is a negative dechallenge.<br>(Note: This criterion is not applicable if: (1) the AE resulted in death or permanent disability; (2) the AE resolved/improved despite continuation of the Merck product; or (3) the trial is a single-dose drug trial); or (4) Merck product(s) is/are only used one time.)
	**Rechallenge**	Was the subject re-exposed to the Merck product in this study?<br>If yes, did the AE recur or worsen?<br>If yes, this is a positive rechallenge. If no, this is a negative rechallenge.<br>(Note: This criterion is not applicable if: (1) the initial AE resulted in death or permanent disability, or (2) the trial is a single-dose drug trial); or (3) Merck product(s) is/are used only one time).<br>NOTE: IF A RECHALLENGE IS PLANNED FOR AN ADVERSE EVENT WHICH WAS SERIOUS AND WHICH MAY HAVE BEEN CAUSED BY THE MERCK PRODUCT, OR IF REEXPOSURE TO THE MERCK PRODUCT POSES ADDITIONAL POTENTIAL SIGNIFICANT RISK TO THE SUBJECT, THEN THE RECHALLENGE MUST BE APPROVED IN ADVANCE BY THE U.S. CLINICAL MONITOR AS PER DOSE MODIFICATION GUIDELINES IN THE PROTOCOL.
	**Consistency with Trial Treatment Profile**	Is the clinical/pathological presentation of the AE consistent with previous knowledge regarding the Merck product or drug class pharmacology or toxicology?

The assessment of relationship will be reported on the case report forms /worksheets by an investigator who is a qualified physician according to his/her best clinical judgment, including consideration of the above elements.	
**Record one of the following**	**Use the following scale of criteria as guidance (not all criteria must be present to be indicative of a Merck product relationship).**
**Yes, there is a reasonable possibility of Merck product relationship.**	There is evidence of exposure to the Merck product. The temporal sequence of the AE onset relative to the administration of the Merck product is reasonable. The AE is more likely explained by the Merck product than by another cause.
**No, there is not a reasonable possibility Merck product relationship**	Subject did not receive the Merck product OR temporal sequence of the AE onset relative to administration of the Merck product is not reasonable OR there is another obvious cause of the AE. (Also entered for a subject with overdose without an associated AE.)

7.1.9 Responsibility for Reporting Adverse Events

All Adverse Events will be reported to regulatory authorities, IRB/IECs and investigators in accordance with all applicable global laws and regulations.

8.0 STATISTICAL ANALYSIS PLAN

8.1 Statistical Analysis Plan Summary

Phase IB part

About 3-6 patients enrollment is expected (dose level 1 and -1). Dose escalation will be proceeded with three patient/cohort until the first DLT (3 week). The RP2D will be defined as the highest dose at which fewer than one out of 3 patients (or 2 out of 6) experience a DLT. If a DLT is observed in three patients, the cohort will be expanded a total of 6 patients before further dose escalation. If 2 or more DLTs are observed in three patients for level 1, the cohort will be deescalated to a lower level (level-1).

Phase II part

On the basis of a ToGA trial, with a minimax, two-stage design:

Successes in 45% or fewer of the patient cases were considered insufficient and did not warrant additional investigation (ie, P0=45%), and successes in 60% or more of the patient cases were sufficient to warrant additional investigation in that particular group (ie, P1= 60%). Applying these hypotheses with 0.05 type I and 0.20 type II errors, if 6 or more successes are observed in the first 13 patients, accrual will be continued until a total of 34 patients. If, of these 34 patients, with 18 or more responses, additional investigation will be warranted.

Allowing for a follow-up loss rate of 10 %, the total sample size is expected as 38 patients

Sample size calculation

For the RP2D dose level in phase I, we will expand phase 2 study for a total of 38 patients as RP2D (3 or more patients with MTD dose in phase I). Finally, about 38~44 patients will be enrolled for this sequential phase IB/II trial. Patients will be treated at the time of disease progression, toxicities, or patient’s refusal

8.2 Statistical Analysis Plan

Analyses of OS and PFS will be performed on the ITT population. For ORR and CBR,only patients in the ITT population with measurable disease at baseline will be included in the analysis. The Kaplan-Meier approach will be used to estimate median OS for each treatment arm with 95% CI. The efficacy variables in this study are PFS, ORR, DoR, and CBR. DoR and CBR will not be included in the multiplicity adjustment

Progression-free survival is defined as the time between the day of first cycle and the date of first documentation of PD or date of death, whichever occurs first. Documentation of disease progression will be defined as per RECIST v1.1 criteria based on investigator assessment. Patients without documented PD or death will be censored at the date of last tumor assessment (or, if no tumor assessments are performed after the baseline visit, at the date of randomization plus 1 day). The Kaplan-Meier approach will be used to estimate the median PFS duration for each treatment arm with 95% CI. The ORR, based on investigator assessments, will be summarized and 95% confidence limits will be calculated using the Clopper-Pearson method.

9.0 LABELING, PACKAGING, STORAGE AND RETURN OF CLINICAL SUPPLIES

9.1 Investigational Product

9.1.1 Pembrolizumab

The investigator shall take responsibility for and shall take all steps to maintain appropriate records and ensure appropriate supply, storage, handling, distribution and usage of investigational product in accordance with the protocol and any applicable laws and regulations.

Clinical Supplies will be provided by Merck as summarized in Table 12.

Table 12. Product Descriptions

**Product Name & Potency**	**Dosage Form**
Pembrolizumab 50 mg	Lyophilized Powder for Injection
Pembrolizumab 100 mg/ 4mL	Solution for Injection

9.1.2 Trastuzumab

A biosimilar drug of trastuzumab, Herzuma (CELLTRION, Inc. Incheon, South Korea) will be provided by CELLTRION. Herzuma is approved for HER2-positive advanced gastric cancer and breast cancer.

**Product Name & Potency**	**Dosage Form**
Herzuma 440mg/vial	Solution for Injection

Regarding, capecitabine and cisplatin where permitted by regulations, sites will obtain and use commercially available drugs. For further details, see the local prescribing information for each agent.

9.2 Packaging and Labeling Information

Clinical supplies will be affixed with a clinical label in accordance with regulatory requirements.

9.3 Clinical Supplies Disclosure

This trial is open-label; therefore, the subject, the trial site personnel, the Sponsor and/or designee are not blinded to treatment. Drug identity (name, strength) is included in the label text, disclosure envelopes or lists are not provided.

9.4 Storage and Handling Requirements

Clinical supplies must be stored in a secure, limited-access location under the storage conditions specified on the label. Receipt and dispensing of trial medication must be recorded by an authorized person at the trial site. Clinical supplies may not be used for any purpose other than that stated in the protocol.

9.5 Returns and Reconciliation

The investigator is responsible for keeping accurate records of the clinical supplies received from Merck or designee, the amount dispensed to and returned by the subjects and the amount remaining at the conclusion of the trial.

Upon completion or termination of the study, all unused and/or partially used investigational product will be destroyed at the site per institutional policy. It is the Investigator's responsibility to arrange for disposal of all empty containers, provided that procedures for proper disposal have been established according to applicable federal, state,

local and institutional guidelines and procedures, and provided that appropriate records of disposal are kept.

10.0 ADMINISTRATIVE AND REGULATORY DETAILS

10.1 Confidentiality

Only the subject number and subject initials will be recorded in the case report form. Study findings stored on a computer will be stored in accordance with local data protection laws. The subjects will be informed that representatives of the institutional review board (IRB), or regulatory authorities may inspect their medical records to verify the information collected, and that all personal information made available for inspection will be handled in strictest confidence and in accordance with the Korean Medical Service Act. The investigator will maintain a personal subject identification list (subject numbers with the corresponding subject names) to enable records to be identified In this multicenter trial, in order to facilitate contact between investigators, the PI may share an investigator's name and contact information with other participating investigators upon request.

10.2 Compliance with Financial Disclosure Requirements

Financial Disclosure requirements are outlined in the US Food and Drug Administration Regulations, Financial Disclosure by Clinical Investigators (21 CFR Part 54). It is the PI's responsibility to determine, based on these regulations, whether a request for Financial Disclosure information is required. It is the investigator's/subinvestigator's responsibility to comply with any such request.

10.3 Compliance with Law, Audit and Debarment

By signing this protocol, the investigator agrees to conduct the trial in an efficient and diligent manner and in conformance with this protocol; generally accepted standards of Good Clinical Practice (e.g., International Conference on Harmonization of Technical Requirements for Registration of Pharmaceuticals for Human Use Good Clinical Practice: Consolidated Guideline and other generally accepted standards of good clinical practice); and all applicable federal, state and local laws, rules and regulations relating to the conduct of the clinical trial.

The investigator also agrees to allow monitoring, audits, IRB/ERC review and regulatory authority inspection of trial-related documents and procedures and provide for direct access to all trial-related source data and documents.

The investigator shall prepare and maintain complete and accurate trial documentation in compliance with Good Clinical Practice standards and applicable federal, state and local laws, rules and regulations; and, for each subject participating in the trial, provide all data, and, upon completion or termination of the clinical trial, submit any other reports to the PI as required by this protocol. Trial documentation will be promptly and fully disclosed to the PI by the investigator upon request and also shall be made available at the trial site upon request for inspection, copying, review and audit at reasonable times by representatives of the regulatory authorities. The investigator agrees to promptly take any reasonable steps that are requested by regulatory authorities as a result of an audit to cure deficiencies in the trial documentation and worksheets/case report forms.

The investigator must maintain copies of all documentation and records relating to the conduct of the trial in compliance with all applicable legal and regulatory requirements. This documentation includes, but is not limited to, the protocol, worksheets/case report forms, advertising for subject participation, adverse event reports, subject source data, correspondence with regulatory authorities and IRBs/ERCs, consent forms, investigator's curricula vitae, monitor visit logs, laboratory reference ranges, laboratory certification or quality control procedures and laboratory director curriculum vitae. It is anticipated that the retention period can be up to 15 years after protocol database lock.

10.4 Compliance with Trial Registration and Results Posting Requirements

Under the terms of the Food and Drug Administration Modernization Act (FDAMA) and the Food and Drug Administration Amendments Act (FDAAA), the PI of the trial is solely responsible for determining whether the trial and its results are subject to the requirements for submission to the Clinical Trials Data Bank, http://www.clinicaltrials.gov. Information posted will allow subjects to identify potentially appropriate trials for their disease conditions and pursue participation by calling a central contact number for further information on appropriate trial locations and trial site contact information.

10.5 Quality Management System

The investigators agrees to be responsible for implementing and maintaining a quality management system with written development procedures and functional area standard operating procedures (SOPs) to ensure that trials are conducted and data are generated, documented, and reported in compliance with the protocol, accepted standards of Good Clinical Practice, and all applicable federal, state, and local laws, rules and regulations relating

to the conduct of the clinical trial.

According to the IDMC (Independent Drug and Safety Monitoring Committee) guideline of KFDA, we will establish DSMC organization to assist and help performing this study.

Members

- Joong-Bae Ahn, M.D., Ph.D: chairman
- Hye Jin Choi, M.D., Ph.D: clinical data and safety monitoring
- In Kyong Jung, statistician: statistical advice

All those memebers' term is from the preparation time of this study to the expected ending time of the study

Committee activity

- The DSMC committee will hold the safety monitoring meeting, and perform regular reviews every 12 months. The final council will be hold within 6 months after the study end. Monitoring results will be officially recorded and reported to the IRB.

10.6 Data Management

The investigator or qualified designee is responsible for recording and verifying the accuracy of subject data.

11.0 APPENDICES

11.1 ECOG Performance Status

Grade	Description
0	Normal activity. Fully active, able to carry on all pre-disease performance without restriction.
1	Symptoms, but ambulatory. Restricted in physically strenuous activity, but ambulatory and able to carry out work of a light or sedentary nature (e.g., light housework, office work).
2	In bed <50% of the time. Ambulatory and capable of all self-care, but unable to carry out any work activities. Up and about more than 50% of waking hours.
3	In bed >50% of the time. Capable of only limited self-care, confined to bed or chair more than 50% of waking hours.
4	100% bedridden. Completely disabled. Cannot carry on any self-care. Totally confined to bed or chair.
5	Dead.
*As published in Am. J. Clin. Oncol.: *Oken, M.M., Creech, R.H., Tormey, D.C., Horton, J., Davis, T.E., McFadden, E.T., Carbone, P.P.: Toxicity And Response Criteria Of The Eastern Cooperative Oncology Group. Am J Clin Oncol 5:649-655, 1982.* The Eastern Cooperative Oncology Group, Robert Comis M.D., Group Chair.	

11.2 Common Terminology Criteria for Adverse Events V4.03 (CTCAE)

The descriptions and grading scales found in the revised NCI Common Terminology Criteria for Adverse Events (CTCAE) version 4.03 will be utilized for adverse event reporting. (http://ctep.cancer.gov/reporting/ctc.html)

11.3 Response Evaluation Criteria in Solid Tumors (RECIST) 1.1 Criteria for Evaluating Response in Solid Tumors

RECIST version 1.1* will be used in this study for assessment of tumor response. While either CT or MRI may be utilized, as per RECIST 1.1, CT is the preferred imaging technique in this study.

* As published in the European Journal of Cancer:

E.A. Eisenhauer, P. Therasse, J. Bogaerts, L.H. Schwartz, D. Sargent, R. Ford, J. Dancey, S. Arbuck, S. Gwyther, M. Mooney, L. Rubinstein, L. Shankar, L. Dodd, R. Kaplan, D. Lacombe, J. Verweij. New response evaluation criteria in solid tumors: Revised RECIST guideline (version 1.1). Eur J Cancer. 2009 Jan;45(2):228-47.

In addition, volumetric analysis will be explored by central review for response assessment.

11.4 Events of Clinical Interest Guidance Document

11.5 References

1. Leffers N, Gooden MJ, de Jong RA, et al. Prognostic significance of tumor-infiltrating T-lymphocytes in primary and metastatic lesions of advanced stage ovarian cancer. Cancer Immunol Immunother. 2009;58:449-59.
2. Lee HE, Chae SW, Lee YJ, et al. Prognostic implications of type and density of tumour-infiltrating lymphocytes in gastric cancer. Br J Cancer. 2008;99:1704-11.
3. Cancer Genome Atlas Research N. Comprehensive molecular characterization of gastric adenocarcinoma. Nature. 2014;513:202-9.
4. Muro K ea. Phase Ib Study of MK-3475 in Patients with Gastric Cancer. Ann Oncol. 2014;25.
5. Ooi A, Kobayashi M, Mai M, Nakanishi I. Amplification of c-erbB-2 in gastric cancer: detection in formalin-fixed, paraffin-embedded tissue by fluorescence in situ hybridization. Lab Invest. 1998;78:345-51.
6. Ougolkov A, Mai M, Takahashi Y, et al. Altered expression of beta-catenin and c-erbB-2 in early gastric cancer. J Exp Clin Cancer Res. 2000;19:349-55.
7. Bang YJ, Van Cutsem E, Feyereislova A, et al. Trastuzumab in combination with chemotherapy versus chemotherapy alone for treatment of HER2-positive advanced gastric or gastro-oesophageal junction cancer (ToGA): a phase 3, open-label, randomised controlled trial. Lancet. 2010;376:687-97.
8. Bellati F, Napoletano C, Ruscito I, Liberati M, Panici PB, Nuti M. Cellular adaptive immune system plays a crucial role in trastuzumab clinical efficacy. J Clin Oncol. 2010;28:e369-70; author reply e71.

9. Ferris RL, Jaffee EM, Ferrone S. Tumor antigen-targeted, monoclonal antibody-based immunotherapy: clinical response, cellular immunity, and immunoescape. J Clin Oncol. 2010;28:4390-9.
10. Bianchini G PA, Pickl M, et al. Response to neoadjuvant trastuzumab and chemotherapy in ER+ and ER- HER2-positive breast cancers; gene expression analysis. Proc Am Soc Clin Oncol. 2011;29:abstr 529.
11. Loi S, Michiels S, Salgado R, et al. Tumor infiltrating lymphocytes are prognostic in triple negative breast cancer and predictive for trastuzumab benefit in early breast cancer: results from the FinHER trial. Ann Oncol. 2014;25:1544-50.
12. L Gianni, G Bianchini, P Valagussa, et al. Adaptive immune system and immune checkpoints are associated with response to pertuzumab (P) and trastuzumab (H) in the NeoSphere study Thirty-Fifth Annual CTRC-AACR San Antonio Breast Cancer Symposium. San Antonio, TX, Dec 4-8, 2012.
13. Staaf J, Ringner M, Vallon-Christersson J, et al. Identification of subtypes in human epidermal growth factor receptor 2--positive breast cancer reveals a gene signature prognostic of outcome. J Clin Oncol. 2010;28:1813-20.
14. Stagg J, Loi S, Divisekera U, et al. Anti-ErbB-2 mAb therapy requires type I and II interferons and synergizes with anti-PD-1 or anti-CD137 mAb therapy. Proc Natl Acad Sci U S A. 2011;108:7142-7.
